# Isomerisation and Insertion Chemistry of Imidosilanes Enabled by Reversible Si(IV)/Si(II) Redox Shuttling

**DOI:** 10.1002/anie.202505872

**Published:** 2025-05-16

**Authors:** Jianqin Tang, Yuwen Wang, Agamemnon E. Crumpton, Caitilín McManus, Simon Aldridge

**Affiliations:** ^1^ Inorganic Chemistry Laboratory Department of Chemistry University of Oxford South Parks Road Oxford OX1 3QR UK

**Keywords:** Main group chemistry, Migration, Redox chemistry, Silicon, Silylene

## Abstract

Spontaneous redox shuttling at silicon is very rare, primarily reflecting the thermodynamic challenges associated with reduction processes for lighter *p*‐block elements. Here we show that the reactions of boryl‐substituted silylene {PhC(*^t^* BuN)_2_}Si{B(NDippCH)_2_} with an organo‐azide proceed through a Si(IV)‐Si(II)‐Si(IV) series of redox processes involving both oxidative and reductive ligand migration steps. Each of the isomeric compounds related through this reaction manifold is isolable (and can be structurally characterised by X‐ray crystallography), with the overall free energy profile being close to thermo‐neutral. Broader studies within group 14 imply that this redox shuttling is unique to silicon and that reductive ligand migration also plays a role in O‐atom insertion chemistry.

## Graphical abstract

We report rearrangement and functionalisation chemistry at silicon facilitated by reductive ligand migration and Si(II)/Si(IV) redox shuttling.

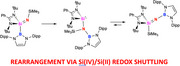

The activation of small molecules by main group compounds has become a highly topical challenge, driven both by fundamental discovery and by environmental imperatives centred around the avoidance of toxic, scarce and increasingly expensive noble metals. Such drivers have led to the development of *p*‐block systems with frontier orbital symmetries and energies appropriate to interact in a synergistic manner with molecules (such as H_2_, CO and alkenes), which are both σ donors and π acceptors).^[^ ^1^, ^2^ ^]^ Much of the derived small molecule activation chemistry reported to date has been stoichiometric, and the involvement of main group compounds in redox catalysis is rare.^[^ ^3^ ^]^ This in turn reflects the fact that for lighter *p*‐block elements, the group oxidation state (*n*) is significantly favoured thermodynamically (and reductive elimination is not favourable); on the other hand, for heavier *p*‐block elements, the *n*‐2 ('Inert Pair') oxidation state is often favoured, and oxidative addition is less easily effected. Examples of thermodynamically reversible processes are therefore relatively scarce.

Within Group 14 chemistry, Bielawski and co‐workers reported the use of an *N,N*′‐diamidocarbene to achieve the reversible activation of ammonia,^[^ ^4^ ^]^ and heavier analogues of germanium and tin have been implicated in reversible bond activation processes.^[^ ^5^, ^6^, ^7^, ^8^, ^9^ ^]^ In the case of silicon‐containing compounds, spontaneous reductive processes leading to the formation of Si(II) species from Si(IV) precursors are rare. The formation of IDipp·SiCl_2_ by the dehydrohalogenation of HSiCl_3_, for example, relies on the accompanying formation of [(IDipp)H]Cl in a formal oxidative process at divalent carbon, which helps drive the reduction at silicon.^[^ ^10^, ^11^, ^12^ ^]^ ‘Acceptor‐less’ processes occurring in the absence of strongly reducing co‐reagents can be enthalpically driven by the formation of a strong bond (e.g., Si─O) and/or entropically driven through the evolution of a volatile co‐product (Scheme 1).^[^ ^13^, ^14^, ^15^, ^16^ ^]^ Similar strategies have also been exploited in examples of *reversible* processes, e.g., the activation of alkenes or E─H bonds by silylene compounds under ambient conditions (Scheme 1).^[^ ^17^, ^18^, ^19^, ^20^ ^]^

**Scheme 1:**
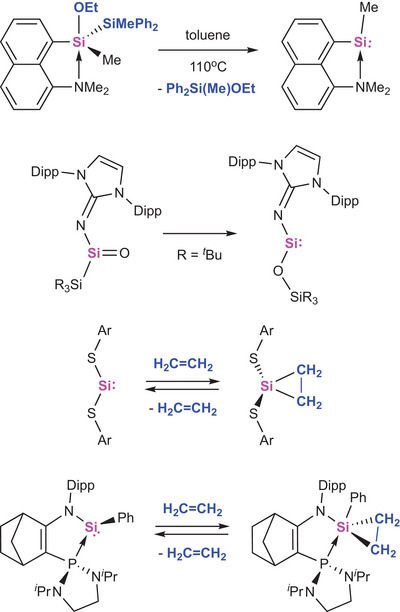
Spontaneous and/or reversible redox processes at silicon of relevance to the current study (Ar = ─C_6_H_3_Dipp_2_‐2,6; Dipp = 2,6‐C_6_H_3_ *^i^* Pr_2_).

We have recently been interested in developing the chemistry of silylene (and related heavier metallylene) systems stabilised by a combination of π‐donating amidinate/guanidinate and strongly σ‐donating boryl ligands.^[^ ^21^ ^]^ In this study, we report the synthesis of an (imido)silane derived from such a silylene and trimethylsilylazide. Most interestingly, we describe both its isomerisation and chemical transformation through reversible cycling between Si(IV) and Si(II) states, enabled by redox‐active ligand migration steps.

The reaction of silylene {PhC(*^t^* BuN)_2_}Si(boryl), (**1**‐Si; boryl = ─B(NDippCH)_2_) with trimethylsilyl azide at room temperature proceeds smoothly to generate (silylimido)silane {PhC(*^t^* BuN)_2_}Si(boryl)(NSiMe_3_) (**2**‐Si; Scheme 2), which can be isolated as a colourless crystalline solid in 65% yield after recrystallisation from pentane. We recently reported a similar synthetic approach to the silanone {PhC(*^t^* BuN)_2_}Si(boryl)(O) by exposing **1**‐Si to N_2_O.^[^ ^21^ ^]^ The identity of the (imido)silane product **2**‐Si has been unambiguously determined by multinuclear NMR spectroscopy, elemental microanalysis and X‐ray crystallography. The ^29^Si NMR signal (δ_Si_ = −49.3 ppm) is shifted noticeably upfield compared to precursor **1**‐Si (δ_Si_ = 57.2 ppm), consistent with formal oxidation from Si(II) to Si(IV) (cf. δ_Si_ = −33.9 ppm for {PhC(*^t^* BuN)_2_}Si(boryl)(O)).^[^ ^21^ ^]^ The ^11^B NMR resonances for **2**‐Si and its silanone analogue (δ_B_ = 23.7 and 24.1 ppm, respectively) are both shifted to high field compared to **1**‐Si (δ_B_ = 29.1 ppm).^[^ ^21^ ^]^

**Scheme 2:**
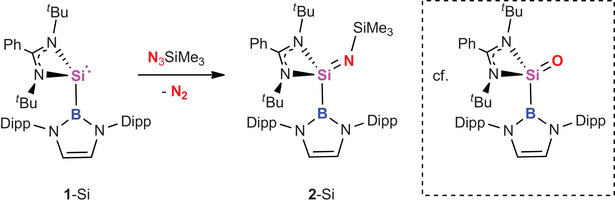
Oxidation of silylene **1**‐Si with trimethylsilylazide to yield (imido)silane **2**‐Si.

Single crystals of **2**‐Si could be grown from pentane solution, and the solid‐state structure determined by X‐ray crystallography (Figure 1, left). The Si─B bond length (2.015(1) Å) is statistically identical to that reported for the silanone (2.016(2) Å) and markedly shorter than that of **1**‐Si (2.066(1) Å), consistent with the smaller covalent radius of Si(IV) over Si(II).^[^ ^21^ ^]^ The Si═N bond (1.597(1) Å) is somewhat longer than the Si═O bond measured for {PhC(*^t^* BuN)_2_}Si(boryl)(O) (1.5406(9) Å)—as expected due to the larger covalent radius of nitrogen (0.71 vs. 0.66 Å).^[^ ^22^ ^]^

**Figure 1:**
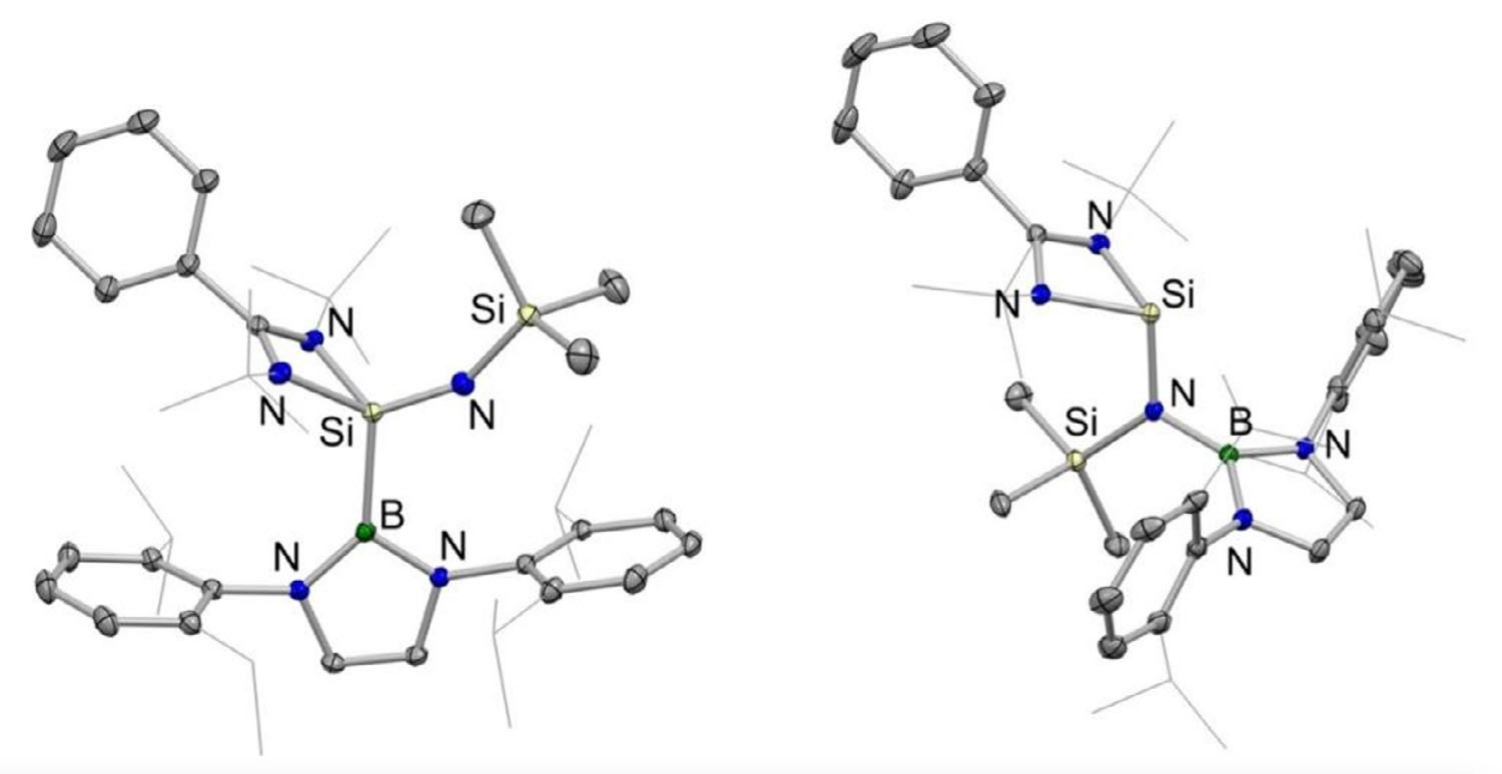
Molecular structures of **2**‐Si (left) and **3**‐Si (right) in the solid state as determined by X‐ray crystallography. Hydrogen atoms omitted and selected groups shown in wireframe format for clarity. Thermal ellipsoids are set at the 30% probability level. Key bond lengths (Å) and angles (°): (for **2**‐Si) Si─N_imide_ 1.597(1), Si─N_amidinate_ 1.872(1), 1.851(1), Si─B 2.015(1), Si─N─Si 152.7(1); (for **3**‐Si) Si─N_amide_ 1.772(1), Si─N_amidinate_ 1.918(1), 1.902(1).

Heating **2**‐Si at 110 °C in toluene over a period of 12 h leads to 90% conversion to a single new species, which is characterised by a ^29^Si NMR signal (δ_Si_ = −11.4 ppm) that is shifted downfield compared to the (imido)silane precursor (**2**‐Si: δ_Si_ = −49.3 ppm) and which is similar to the signals reported for amidinate‐supported silylenes (e.g., {PhC(*^t^* BuN)_2_}Si(NMe_2_): δ_Si_ = −2.6 ppm; {PhC(*^t^* BuN)_2_}Si{N(SiMe_3_)_2_}: δ_Si_ = −8.1 ppm).^[^ ^23^, ^24^ ^]^ The product of this thermal rearrangement (**3**‐Si) could be isolated as a pale green crystalline solid in 50% yield by recrystallisation from pentane, and its identity as a silylene confirmed by X‐ray crystallography (Scheme 3 and Figure 1, right). 1,2‐Migration of the Si‐bound boryl group to nitrogen leads to the formation of the sterically encumbered amido ligand —N(boryl)(SiMe_3_), which is bound to Si(1) through a relatively long Si─N bond (1.772(1) Å). This distance can be put into context by Si─N separations of 1.724(2) Å measured for the less crowded dimethylamido derivative {PhC(*^t^* BuN)_2_}Si(NMe_2_) and 1.769(7) Å for the more closely comparable bis(trimethylsilyl)amido‐silylene {PhC(*^t^* BuN)_2_}Si{N(SiMe_3_)_2_}.^[^ ^23^, ^24^ ^]^ Consistently, the methyl and methine ^1^H NMR signals for the 2,6‐diisopropylphenyl (Dipp) groups of **3**‐Si are broad, due to restricted boryl group rotation on the NMR timescale; these signals sharpen on raising the temperature to 45 °C.

The transformation of **2**‐Si into **3**‐Si represents a rare example of spontaneous conversion of Si(IV) to Si(II) under thermal conditions without the addition of a reducing agent or the evolution of a volatile co‐product.^[^ ^12^, ^13^, ^14^, ^15^ ^]^ Most closely related in the current context is the silyl‐silanone to oxy‐silylene conversion reported by Rieger and Inoue in 2017, driven by thermodynamically favourable Si─O (single) bond formation.^[^ ^16^ ^]^ The feasibility of the conversion of **2**‐Si to **3**‐Si is supported by DFT calculations which suggest that boryl migration is exergonic by 5.0 kcal mol^−1^ and incurs an activation barrier of ca. 30 kcal mol^−1^ (consistent with a reaction that needs heating at 110 °C for 12 h; see below).

**Scheme 3:**
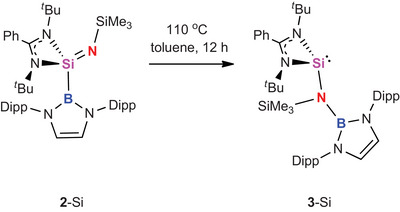
Thermal rearrangement of sila‐imine **2**‐Si to (borylamido) silylene 3‐Si.

Interestingly, more extended heating of **2**‐Si (or heating of **3**‐Si over a period of 36 h) leads to further onwards conversion to a species characterised by a ^29^Si signal in the region expected for an imidosilane derivative (δ_Si_ = −57.6 ppm cf. −49.3 ppm for **2**‐Si). This new compound is also characterised by a markedly upfield shifted signal associated with the SiMe_3_ group (δ_Si_ = −24.6 ppm, cf. −0.1 ppm for **3**‐Si). Single crystals of the product (**4**) suitable for single crystal X‐ray diffraction could be isolated from the reaction mixture, and confirm the formation of a (borylimido)silane via 1,2‐transfer of the trimethylsilyl group from N to Si (Scheme 4 and Figure 2). The conversion of **3**‐Si to **4** proves impossible to drive to completion in our hands; the fact that it reaches equilibrium at ca. 70% conversion is consistent with DFT calculations which imply that the free energy difference between **3**‐Si and **4** is very small (see below).

**Scheme 4:**
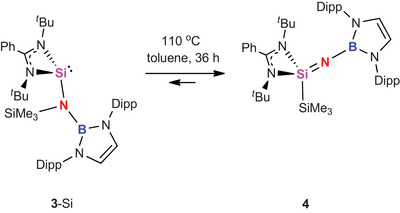
Regeneration of Si(IV) through N‐to‐Si migration of the silyl substituent under forcing conditions.

**Figure 2:**
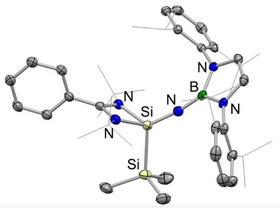
Molecular structure of **4** in the solid state as determined by X‐ray crystallography. Hydrogen atoms omitted and selected groups shown in wireframe format for clarity. Thermal ellipsoids are set at the 30% probability level. Key bond lengths (Å) and angles (°): Si─N_imide_ 1.581(4), B─N_imide_ 1.383(5), Si─N_amidinate_ 1.863(3), 1.837(4), Si─Si 2.356(1), Si─N─B 169.3(3).

In order to gain insight into the mechanism of the redox shuttling at silicon linking compounds **2**‐Si, **3**‐Si and **4**, DFT calculations were performed at the R2‐SCAN‐3c level of theory (Figure 3). The first step in the reaction pathway is the migration of the boryl group in **2**‐Si from silicon to nitrogen, which occurs with an activation barrier of 29.4 kcal mol^−1^ and is modestly exergonic (ΔG = −5.0 kcal mol^−1^). That the resulting (amido)silylene **3**‐Si is more stable than the isomeric (imido)silane is presumably—at least in part—due to the formation of a strong B─N bond. **3**‐Si is highly sterically congested (as reflected in the broad signals for the boryl Dipp substituents seen in its ^1^H NMR spectrum), and attempts to model an onwards isomerisation process which involves rotation about the Si─N bond (prior to N‐to‐Si migration of the SiMe_3_ substituent) incur a prohibitively high activation barrier (> 45 kcal mol^−1^). The lowest energy pathway which we have identified for this second step involves silyl ligand migration occurring concurrently with inversion at silicon through ‘flipping’ of the amidinate ligand. This results in the formation of (borylimido)silane **4**, via a process which is only slightly exergonic (ΔG = −1.7 kcal mol^−1^) and occurs via a high activation barrier of +37.1 kcal mol^−1^. While this barrier is high, it speaks to the reaction conditions required (110 °C, over 36 h) for the establishment of the equilibrium implied by the near thermoneutrality of the transformation.

**Figure 3:**
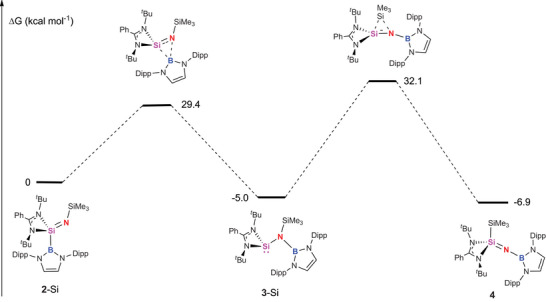
DFT calculated mechanism for the isomerisation of (imido)silanes **2**‐Si and **4** via silylene **3**‐Si (calculated at the R2‐SCAN‐3c method level of theory with solvation modelled with C‐PCM, toluene). Inset: alternative reaction pathways accessed via a transition state involving rotation about the Si─N bond in **3**‐Si incur much higher activation barriers, presumably on steric grounds.

In order to probe the wider relevance of this redox shuttling, the corresponding chemistry of stannylene **1**‐Sn was also investigated (Scheme 5). These results are consistent with the greater thermodynamic stability of the +2 oxidation state for tin over silicon. Accordingly, (silylimido)stannane **2**‐Sn can be accessed using Me_3_SiN_3_ via a similar synthetic approach to **2**‐Si and crystallised from minimal pentane over a period of several minutes. However, in contrast to its silicon congener, **2**‐Sn is transformed in a facile manner (12 h at room temperature) into tin(II) amide **3**‐Sn, via an Sn‐to‐N boryl substituent migration analogous to that seen for **2**‐Si/**3**‐Si.

**Scheme 5:**
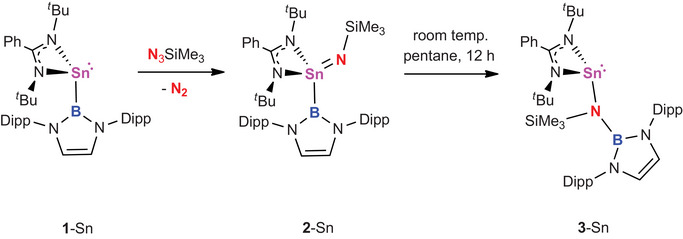
(Silylimido)stannane formation from **1**‐Sn and Me_3_SiN_3_ and subsequent reductive Sn‐to‐N boryl substituent migration to yield (amido)stannylene **3**‐Sn.

The molecular structures of **2**‐Sn and **3**‐Sn obtained crystallographically confirm the migration of the boryl substituent from tin to nitrogen (Figure 4). Structural features for these two compounds largely resemble their silicon counterparts, although the Sn─N─Si angle for **2**‐Sn (131.2(1)°) is significantly narrower than the Si─N─Si angle for **2**‐Si (152.7(1)°), presumably reflecting the smaller size of Si (over Sn) and the greater angular separation therefore required to minimise repulsive steric interactions with the silylimido substituent.

**Figure 4:**
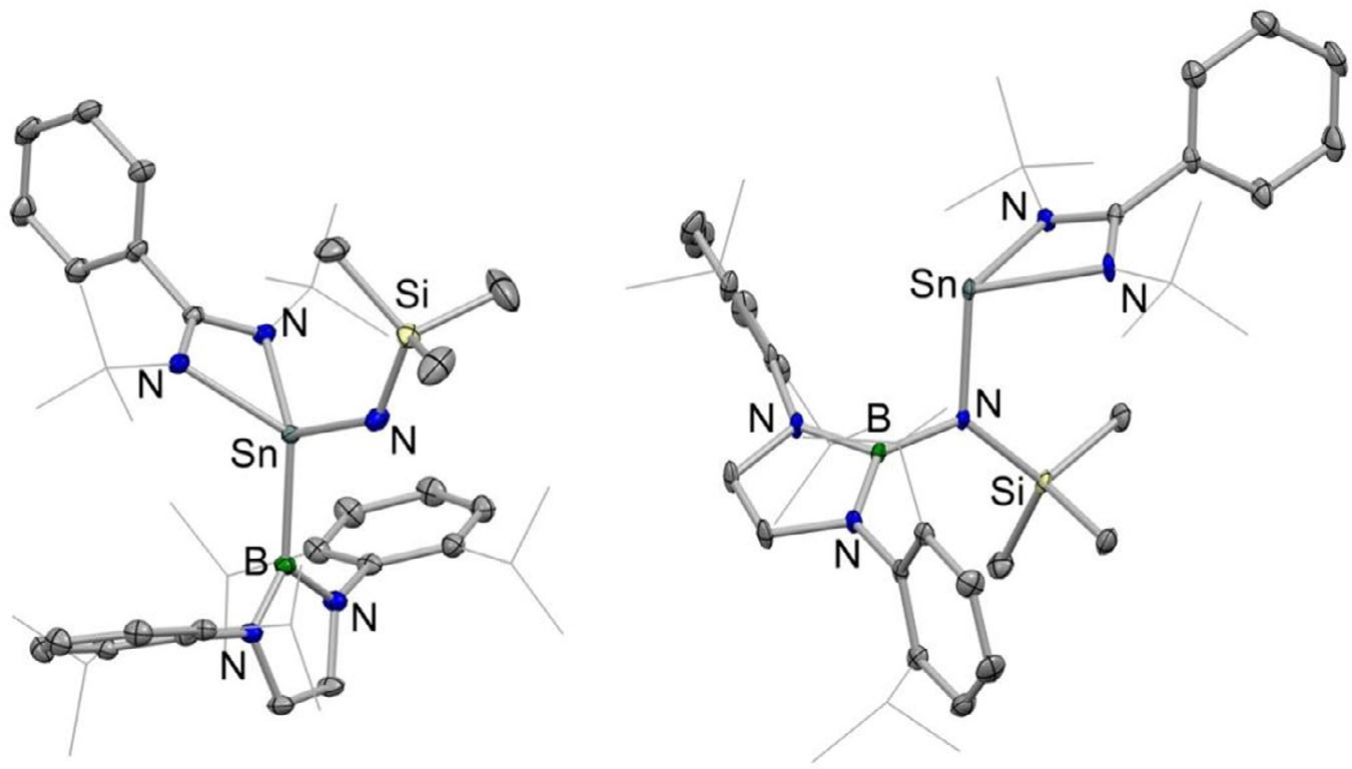
Molecular structures of **2**‐Sn (left) and **3**‐Sn (right) in the solid state as determined by X‐ray crystallography. Hydrogen atoms omitted and selected groups shown in wireframe format for clarity. Thermal ellipsoids are set at the 30% probability level. Key bond lengths (Å) and angles (°): (for **2**‐Sn) Sn─N_imide_ 1.914(1), Sn─N_amidinate_ 2.176(1), 2.185(1), Sn─B 2.250(2), Sn─N─Si 131.2(1); (for **3**‐Sn) Sn─N_amide_ 2.118(1), Sn─N_amidinate_ 2.230(1), 2.230(1).

No evidence for onward conversion of **3**‐Sn to a Sn(IV) borylimide species analogous to **4** was obtained experimentally, consistent with the enhanced stability of the E(II) oxidation state lower down group 14. Moreover, this observation is supported by DFT calculations which imply that the free energy of an Sn(IV) species analogous to **4** would be much higher than **3**‐Sn (ca. +46.1 kcal mol^−1^; Figure S25). Similar observations pertain for related (hypothetical) germanium systems (Figure S25), and the idea of shuttling between E(II) and E(IV) states in this ligand system is therefore unique to silicon.

The implications of ligand migration and redox reversibility in the context of *chemical reactivity* are illustrated by the reaction of **2**‐Si with N_2_O. This reaction results in formal O‐atom insertion into the Si─B bond to give boryloxy compound **5** (Scheme 6 and Figure 5).^[^ ^25^ ^]^ The reaction of **3**‐Si with N_2_O gives the same product, implying that mechanistically this process proceeds via reductive ligand migration to generate silylene **3**‐Si, followed by O‐atom transfer from N_2_O, and finally N‐to‐O migration of the boryl group driven thermodynamically by B─O bond formation (and kinetically facilitated by the highly polar Si═O bond). Similar N‐to‐O migration has precedent in the reaction of (boryl){(Me_3_Si)DippN}Si with N_2_O.^[^ ^26^ ^]^ The fact that the conversion of **2**‐Si to **5** requires heating to 110 °C, while the conversion of 3‐Si to **5** happens at room temperature, is consistent with the (forcing) conditions delineated above for the conversion of **2**‐Si to **3**‐Si.

**Scheme 6:**
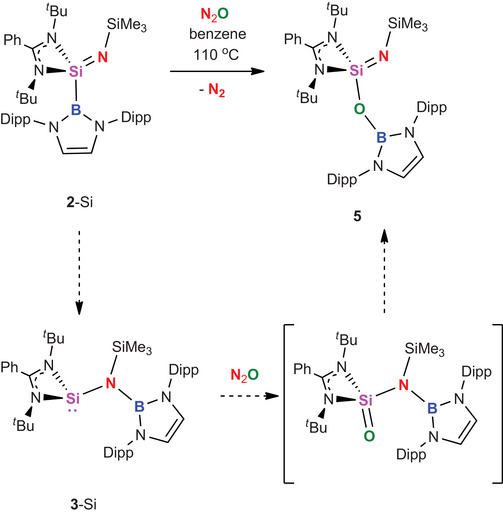
Formal O‐atom insertion into the Si‐B bond of **2**‐Si, facilitated by reductive ligand migration to give silylene intermediate **3**‐Si.

**Figure 5:**
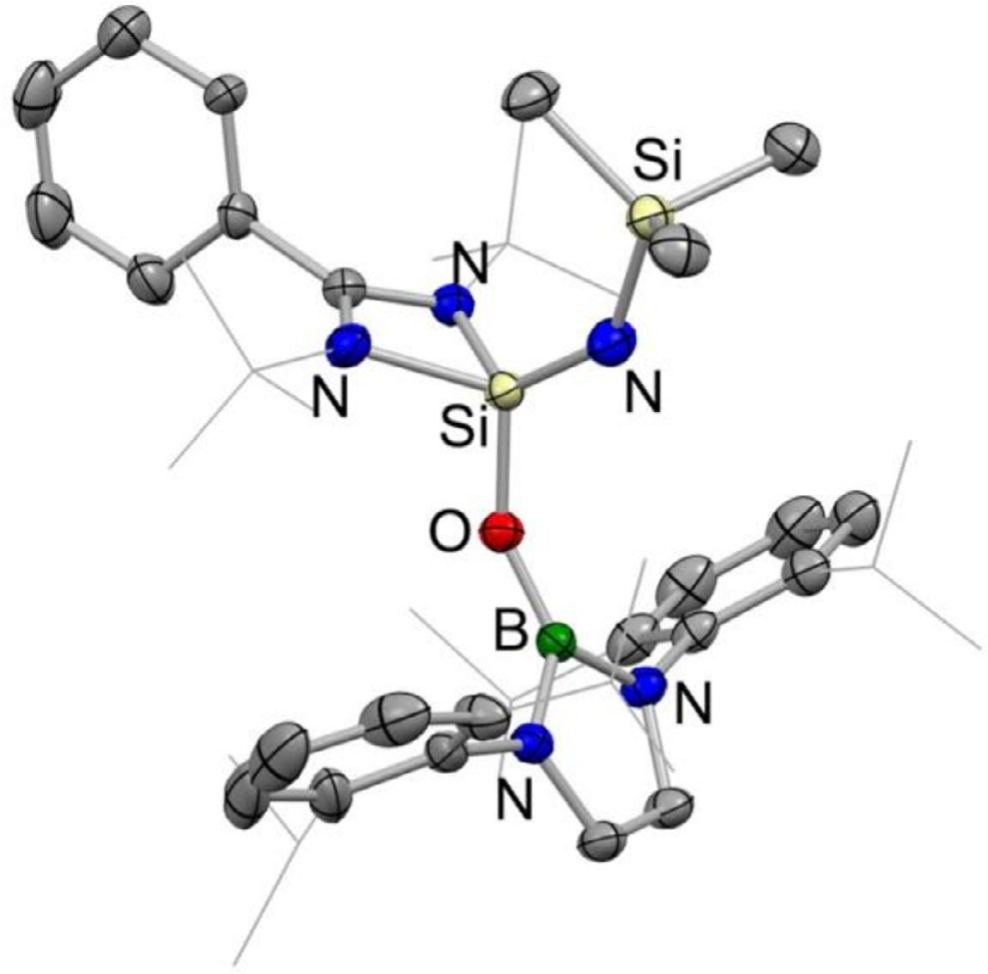
Molecular structure of **5** in the solid state as determined by X‐ray crystallography. Hydrogen atoms omitted and selected groups shown in wireframe format for clarity. Thermal ellipsoids set at the 30% probability level. Key bond lengths (Å) and angles (°): Si─N_imide_ 1.590(2), Si─N_amidinate_ 1.827(2), 1.829(2), Si─O 1.616(1), B─O 1.363(2), Si─N─Si 139.5(1), Si─O─B 149.1(1).

To conclude, in this work we have shown that the reaction of boryl‐substituted silylene {PhC(*^t^* BuN)_2_}Si(boryl) (**1**‐Si) with trimethylsilylazide accesses a reaction manifold connected by a series of Si(IV)‐Si(II)‐Si(IV) steps, involving formal oxidative and reductive ligand migration processes. Quantum chemical calculations indicate that the Si(IV) species (i.e., imidosilanes **2**‐Si and **4**) and the Si(II) species (amidosilylene **3**‐Si) are very similar in energetic terms (within 5 kcal mol^−1^). The stability of the Si(IV) species can be related to the strong σ‐donor properties of the silyl and boryl ligands, while the stabilization of **3**‐Si is related to the π‐donor capabilities of the amidinate scaffold and to the relief of steric crowding at silicon due to migration of the highly encumbered boryl group. Wider studies within group 14 imply that these redox shuttling capabilities are unique to silicon, with the related germanium and tin systems weighted thermodynamically towards the E(II) state. In a broader context, these findings highlight the potential for rational ligand design to enable reversible redox processes centred on main‐group element compounds.

## Supporting Information

The data that support the findings of this study are available in the supplementary material of this article.^[^ ^27^ ^]^ The supplementary material cites additional references.^[^ ^28^, ^29^, ^30^, ^31^, ^32^, ^33^, ^34^, ^35^, ^36^ ^]^

## Conflict of Interests

The authors declare no conflict of interest.

## Supporting information



Supporting Information S1

Supporting Information S2

## Data Availability

The data that support the findings of this study are available in the supplementary material of this article.
