# Supplementary material for: Isomerisation and Insertion Chemistry of Imidosilanes Enabled by Reversible Si(IV)/Si(II) Redox Shuttling
Source: Angew Chem Int Ed Engl. 2025 May 16;64(26):e202505872. doi: 10.1002/anie.202505872 (PMC12184292; doi:10.1002/anie.202505872)
Supplement: Supplementary file 1 — Supporting Information S1 [file ANIE-64-e202505872-s002.pdf]

Isomerization and insertion chemistry of imidosilanes enabled by reversible Si(IV)/Si(II) redox shuttling

Jianqin Tang, Yuwen Wang, Agamemnon E. Crumpton, Caitilín. McManus, Simon Aldridge*

Inorganic Chemistry Laboratory, Department of Chemistry, University of Oxford, South Parks Road, Oxford, OX1 3QR (UK)

simon.aldridge@chem.ox.ac.uk

Supporting Information (48 pages)

1. General considerations s2

2. Preparation of starting materials s2

3. Synthetic procedures and characterising data for new compounds s3

4. Representative NMR spectra for new compounds s9

5. Details of X-ray crystallography s23

6. Details of quantum chemical calculations s26

7. References for supporting information s48

1. General considerations

All experiments were carried out under an atmosphere of dry argon or dinitrogen using standard Schlenk line and dry-box techniques. Solvents were degassed by sparging with argon and dried by passing through a column of appropriate drying agent using a commercially available Braun SPS and stored over potassium mirror under argon in a Teflon valve ampoule. NMR spectra were recorded in d_6-benzene or d_8-toluene, with the solvent being dried over calcium hydride or potassium respectively, distilled, degassed by three freeze-pump-thaw-cycles and stored over 3 Å sieves. NMR samples were prepared under argon in 5 mm Wilmad 507-PP tubes fitted with J. Young Teflon valves. ^{1}H and ^{13}C{^{1}H} NMR spectra were measured on Bruker Avance III HD nanobay 400 MHz, or Bruker Avance III 500 MHz, and referenced internally to residual protio-solvent (^{1}H) or solvent (^{13}C) resonances and are reported relative to tetramethylsilane (δ = 0 ppm). ^{11}B, ^{29}Si and ^{119}Sn spectra were referenced to external $BF_3{\cdot}Et_2O$, $SiMe_4$ and $SnMe_4$, respectively. Assignments were confirmed using two-dimensional ^{1}H-^{1}H, ^{13}C-^{1}H, ^{1}H-^{29}Si NMR correlation experiments. Chemical shifts are quoted in δ (ppm) and coupling constants in Hz. The reported yields are the yields obtained after crystallisation and subsequent measurement of the material by single crystal-ray diffraction if not stated otherwise. Elemental analyses were carried out by London Metropolitan University or Elemental Microanalysis Ltd, Okehampton, Devon, UK.

2. Preparation of starting materials

1-Si and **1**-Sn were prepared according to methods reported in the primary literature.[s1]

3. Synthetic procedures and characterising data for new compounds

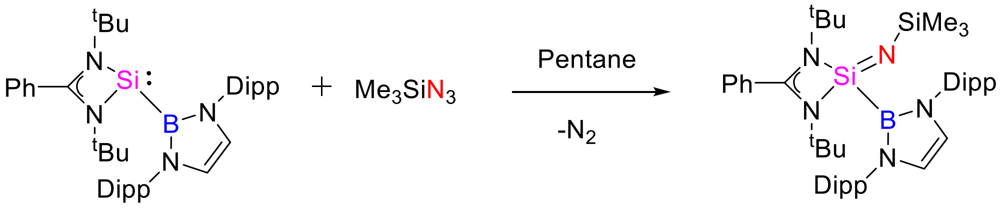

1-Si **2**-Si

{PhC(^{t}BuN)$_2$}{(HCDippN)$_2$B}SiN(SiMe$_3$), **2**-Si

1-Si (200 mg, 0.31 mmol) was dissolved in pentane (30 mL) and Me_3SiN_3 (178.3 mg, 1.55 mmol) added dropwise, leading to bubbling and a colour change from yellow to colourless. After 1 h the solution was concentrated to a third of its original volume, and colourless crystals of **2**-Si obtained on standing at -30 °C which were suitable for X-ray crystallography. These were isolated by filtration and washed with cold pentane (2 x 5 mL).

Yield: 140 mg (0.19 mmol, 64.5 %).

Elemental microanalysis: calc. for $C_{44}H_{68}BN_5Si_2$ (%): C 72.00 H 9.34 N 9.54; meas. C 72.37 H 9.07 N 8.97.

^{1}H NMR (500 MHz, C_6D_6, 298 K): δ_H 0.26 (s, 9H, $SiMe_3$), 1.04 (s, 18H, ^{t}Bu), 1.23 (d, $^3J_{H,H}$ = 6.7 Hz, 12H, CH_3 of Dipp), 1.48 (d, $^3J_{H,H}$ = 6.9 Hz, 12H, CH_3 of Dipp), 3.34 (sept, $^3J_{H,H}$ = 6.8 Hz, 4H, C<u>H</u>(CH_3)$_2$ of Dipp), 6.28 (s, 2H, CH of boryl), 6.83, 6.92, 7.20 (m, 5H, ArH of Ph), 7.27-7.34 (m, 6H, ArH of Dipp).

^{11}B{^{1}H} NMR (160 MHz, C_6D_6, 298 K): δ_B 24.5.

^{13}C{^{1}H} NMR (126 MHz, C_6D_6, 298 K): δ_C 6.5 ($SiMe_3$), 23.7, 26.7 (CH_3 of Dipp), 28.8 (<u>C</u>H(CH_3)$_2$ of Dipp), 31.8 (CH_3 of ^{t}Bu), 53.8 (<u>C</u>CH_3 of ^{t}Bu), 123.4 (CH of boryl), 123.6 (m-Ar of Dipp), 127.4 (p-Ar of Dipp), 127.5, 128.0, 128.4, 128.7, 130.0, 132.3 (Ph), 142.4 (CN of Dipp), 145.9 (o-Ar of Dipp), 174.8 (NCN of backbone).

^{29}Si{^{1}H} NMR (99 MHz, C_6D_6, 298 K): δ_{Si} -23.3 (s, $SiMe_3$), -49.3 (br, Si=N).

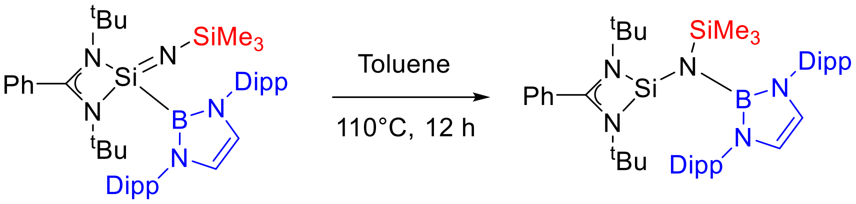

2-Si **3**-Si

{PhC(^{t}BuN)$_2$}SiN{B(NDippCH)$_2$}(SiMe$_3$), **3**-Si

2-Si (200 mg, 0.27 mmol) was dissolved in toluene (10 mL) and stirred for 12 h at 110 °C. The reaction mixture was concentrated (to ca. 1 mL) and yellow crystals of **3**-Si obtained on standing at room temperature which were suitable for X-ray crystallography. These were isolated by filtration, washed twice with cold pentane (2 x 0.5 mL) and dried under vacuum.

Yield: 100 mg (0.14 mmol, 50.0 %).

Elemental microanalysis: calc. for $C_{44}H_{68}BN_5Si_2$ (%): C 72.00 H 9.34 N 9.54; meas. C 71.15 H 9.08 N 9.54.

^{1}H NMR (500 MHz, C_6D_6, 298 K): δ_H 0.33 (s, 9H, SiMe$_3$), 0.88 (s, 18H, ^{t}Bu), 1.33-1.74 (br, 24H, CH$_3$ of Dipp), 3.89 (br, 4H, C$\underline{H}$(CH$_3$)$_2$ of Dipp), 6.16 (s, 2H, CH of boryl), 6.87, 7.13 (m, 5H, ArH of Ph), 7.26 (br, 6H, ArH of Dipp).

^{11}B{^{1}H} NMR (160 MHz, C_6D_6, 298 K): δ_B 26.1.

^{13}C{^{1}H} NMR (126 MHz, C_6D_6, 298 K): δ_C 6.2 (SiMe$_3$), 23.1, 27.1 (CH$_3$ of Dipp), 28.4, 29.6 ($\underline{C}$H(CH$_3$)$_2$ of Dipp), 32.2 (CH$_3$ of ^{t}Bu), 53.7 ($\underline{C}$CH$_3$ of ^{t}Bu), 119.9 (CH of boryl), 123.9 (m-Ar of Dipp), 127.4 (p-Ar of Dipp), 127.6, 128.0, 128.4, 129.1, 130.7, 135.2 (Ph), 141.5 (CN of Dipp), 147.0, 148.7 (o-Ar of Dipp), 159.9 (NCN of backbone).

^{29}Si{^{1}H} NMR (99 MHz, C_6D_6, 298 K): δ_{Si} -0.1 (s, SiMe$_3$), -11.4 (s, silylene).

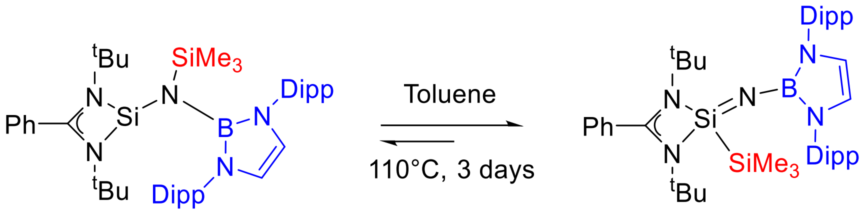

3-Si **4** (70%)

{PhC(^{t}BuN)$_2$}(Me$_3$Si)SiN{B(NDippCH)$_2$}, **4**

3-Si (100 mg, 0.14 mmol) was dissolved in toluene (5 mL) and stirred for 3 d at 110 °C. Volatiles were removed under vacuum, and the residue extracted with pentane (10 mL). The resulting solution was concentrated to half of its original volume and stored at – 30 °C, leading to the formation of light green crystals of **4** suitable for X-ray crystallography. These were isolated by filtration and washed with cold pentane (2 x 1 mL).

Yield: 24 mg (0.03 mmol, 24.0 %).

^{1}H NMR (400 MHz, C_6D_6, 298 K): δ_H 0.22 (s, 9H, SiMe$_3$), 0.78 (s, 18H, ^{t}Bu), 1.45 (d, $^3J_{H,H}$ = 6.7 Hz, 12H, CH$_3$ of Dipp), 1.53 (d, $^3J_{H,H}$ = 6.7 Hz, 12H, CH$_3$ of Dipp), 3.86 (sept, $^3J_{H,H}$ = 6.7 Hz, 4H, C<u>H</u>(CH$_3$)$_2$ of Dipp), 6.21 (s, 2H, CH of boryl), 6.79, 6.90, 7.20 (m, 5H, ArH of Ph), 7.27 (m, 6H, ArH of Dipp).

^{11}B{^{1}H} NMR (128 MHz, C_6D_6, 298 K): δ_B 22.0.

^{13}C{^{1}H} NMR (126 MHz, C_6D_6, 298 K): δ_C 0.1 (TMS), 24.5, 25.1 (CH$_3$ of Dipp), 28.7 (<u>C</u>H(CH$_3$)$_2$ of Dipp), 31.6 (CH$_3$ of ^{t}Bu), 53.4 (<u>C</u>CH$_3$ of ^{t}Bu), 117.2 (CH of boryl), 123.2 (m-Ar of Dipp), 125.8 (p-Ar of Dipp), 128.0, 128.4, 128.5, 128.6, 130.1, 131.6 (Ph), 143.9 (CN of Dipp), 147.5 (o-Ar of Dipp), 171.5 (NCN of backbone).

^{29}Si{^{1}H} NMR (99 MHz, C_6D_6, 298 K): δ_{Si} -24.6 (s, TMS), -57.6 (s, Si=N).

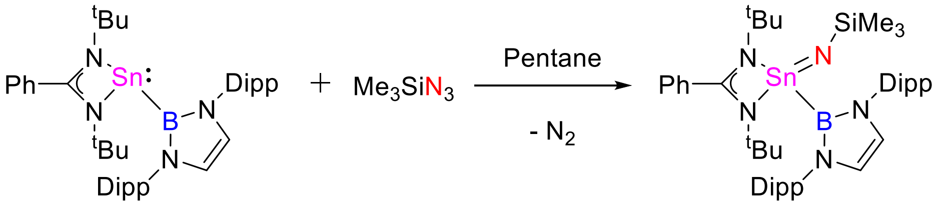

1-Sn **2**-Sn

{PhC(^{t}BuN)$_2$}{(HCDippN)$_2$B}SnN(SiMe$_3$), **2**-Sn

1-Sn (200 mg, 0.24 mmol) was dissolved in pentane (10 mL) and Me_3SiN_3 (138.1 mg, 1.20 mmol) added dropwise, leading to bubbling and a colour change from orange to colourless. Colourless crystals of **2**-Sn suitable for X-ray crystallography were obtained on standing at room temperature. These were filtered and washed with cold pentane (2 x 5 mL).

The solubility of **2**-Sn is low in toluene at –30 °C and it is not stable in THF at room temperature.

Yield: 100 mg (0.12 mmol, 50.0 %).

Elemental microanalysis: calc. for $C_{44}H_{68}BN_5SiSn$ (%): C 64.08 H 8.31 N 8.49; meas. C 64.08 H 8.28 N 7.89.

^{1}H NMR (400 MHz, C_6D_6, 298 K): δ_H 0.63 (s, 9H, SiMe$_3$), 0.69 (s, 18H, ^{t}Bu), 1.21 (d, $^3J_{H,H}$ = 6.7 Hz, 12H, CH_3 of Dipp), 1.51 (d, $^3J_{H,H}$ = 6.8 Hz, 12H, CH_3 of Dipp), 3.46 (sept, $^3J_{H,H}$ = 6.7 Hz, 4H, C$\underline{H}$(CH_3)$_2$ of Dipp), 6.34 (s, 2H, CH of boryl), 6.88, 6.92, 6.96, 7.07, 7.22, 7.36 (m, 11H, ArH).

^{11}B{^{1}H} NMR (128 MHz, C_6D_6, 298 K): δ_B 30.8.

^{13}C{^{1}H} NMR (126 MHz, d$_8$-toluene, 223 K): δ_C 8.1 (SiMe$_3$), 24.8, 26.2 (CH_3 of Dipp), 29.2 ($\underline{C}$H(CH_3)$_2$ of Dipp), 32.7 (CH_3 of ^{t}Bu), 53.4 ($\underline{C}CH_3$ of ^{t}Bu), 124.5 (CH of boryl), 146.8 (o-Ar of Dipp). * Other Ar peaks obscured by d$_8$-toluene signals; N$\underline{C}$N signal of backbone not observed because of low solubility

^{119}Sn{^{1}H} NMR: not observed.

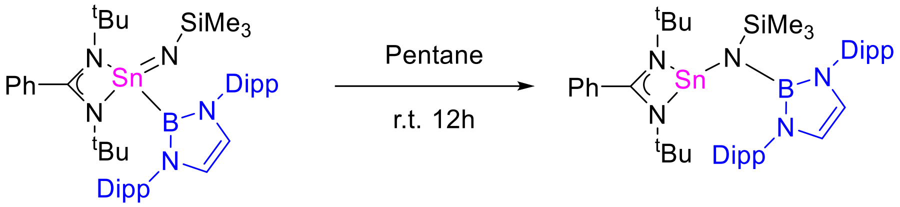

2-Sn **3**-Sn

{PhC(^{t}BuN)$_2$}SnN{B(NDippCH)$_2$}(SiMe$_3$), **3**-Sn.

A solution of **2**-Sn (100 mg, 0.12 mmol) in pentane (20 mL) was stirred for 12 h at room temperature. The reaction mixture was slowly concentrated (to ca. 0.5 mL) and colourless crystals of **3**-Sn were obtained on standing at room temperature which were suitable for X-ray crystallography. These were isolated by filtration and washed with cold pentane (2 x 0.5 mL).

Yield: 43 mg (0.05 mmol, 43.0 %).

Elemental microanalysis: calc. for $C_{44}H_{68}BN_5SiSn$ (%): C 64.08 H 8.31 N 8.49; meas. C 64.09 H 8.09 N 8.15.

^{1}H NMR (500 MHz, C_6D_6, 298 K): δ_H 0.28 (s, 9H, SiMe$_3$), 0.84 (s, 18H, ^{t}Bu), 1.31 (d, $^3J_{H,H}$ = 6.5 Hz, 12H, CH$_3$ of Dipp), 1.50 (br, 12H, CH$_3$ of Dipp), 3.77 (br, 4H, C<u>H</u>(CH$_3$)$_2$ of Dipp), 6.16 (s, 2H, CH of boryl), 6.89, 6.95, 7.19 (m, 5H, ArH of Ph), 7.24 (m, 6H, ArH of Dipp).* δ_H 0.30 (silicone grease).

^{11}B{^{1}H} NMR (160 MHz, C_6D_6, 298 K): δ_B 25.4.

^{13}C{^{1}H} NMR (126 MHz, C_6D_6, 298 K): δ_C 5.2 (SiMe$_3$), 23.4, 26.4 (CH$_3$ of Dipp), 28.7 (br, <u>C</u>H(CH$_3$)$_2$ of Dipp), 33.0 (CH$_3$ of ^{t}Bu), 52.6 (<u>C</u>CH$_3$ of ^{t}Bu), 119.6 (CH of boryl), 123.8 (br, m-Ar of Dipp), 127.4 (p-Ar of Dipp), 126.8, 127.3, 128.4, 128.9, 129.5, 138.6 (Ph), 141.5 (CN of Dipp), 147.1 (o-Ar of Dipp), 168.3 (NCN of backbone).

^{119}Sn{^{1}H} NMR (187 MHz, C_6D_6, 298 K): δ_{Sn} -50.0 (br).

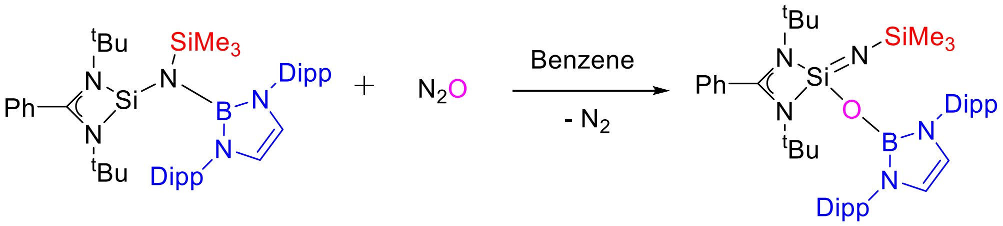

3-Si **5**

{PhC(^{t}BuN)$_2$}{(HCDippN)$_2$BO}SiN(SiMe$_3$), **5**. A solution of **3**-Si (20 mg 0.03 mmol) in benzene and degassed by the freeze-pump-thaw before N_2O was admitted (at ca. 1 atm pressure), leading to a colour change from yellow to colourless. Volatiles were removed under vacuum, and pentane (0.5 mL) added. Crystals of **5** were obtained on standing at room temperature which were suitable for X-ray crystallography.

Yield: 5 mg (0.007 mmol, 24.5 %).

Elemental microanalysis: calc. for $C_{44}H_{68}BN_5OSi_2$ (%): C 70.46 H 9.14 N 9.34; meas. C 70.52 H 9.18 N 9.17.

^{1}H NMR (500 MHz, C_6D_6, 298 K): δ_H 0.54 (s, 9H, SiMe$_3$), 0.71 (s, 18H, ^{t}Bu), 1.31 (d, $^3J_{H,H}$ = 6.8 Hz, 12H, CH$_3$ of Dipp), 1.57 (d, $^3J_{H,H}$ = 6.8 Hz, 12H, CH$_3$ of Dipp), 3.61 (sept, $^3J_{H,H}$ = 6.8 Hz, 4H, C$\underline{H}$(CH$_3$)$_2$ of Dipp), 6.04 (s, 2H, CH of boryl), 6.78, 6.84, 6.93 (m, 5H, ArH of Ph), 7.19 (m, 6H, ArH of Dipp).

^{11}B{^{1}H} NMR (160 MHz, C_6D_6, 298 K): δ_B 21.2.

^{13}C{^{1}H} NMR (126 MHz, C_6D_6, 298 K): δ_C 6.3 (SiMe$_3$), 24.6, 25.2 (CH$_3$ of Dipp), 28.8 ($\underline{C}$H(CH$_3$)$_2$ of Dipp), 30.8 (CH$_3$ of ^{t}Bu), 53.7 ($\underline{C}$CH$_3$ of ^{t}Bu), 117.6 (CH of boryl), 123.8 (m-Ar of Dipp), 127.5 (p-Ar of Dipp), 128.0, 128.2, 128.4, 130.2, 130.7(Ph), 139.6 (CN of Dipp), 147.5 (o-Ar of Dipp), 175.9 (NCN of backbone).

^{29}Si{^{1}H} NMR (99 MHz, C_6D_6, 298 K): δ_{Si} -21.7 (s, SiMe$_3$), -82.7 (s, Si=N).

4. Representative NMR spectra for new compounds

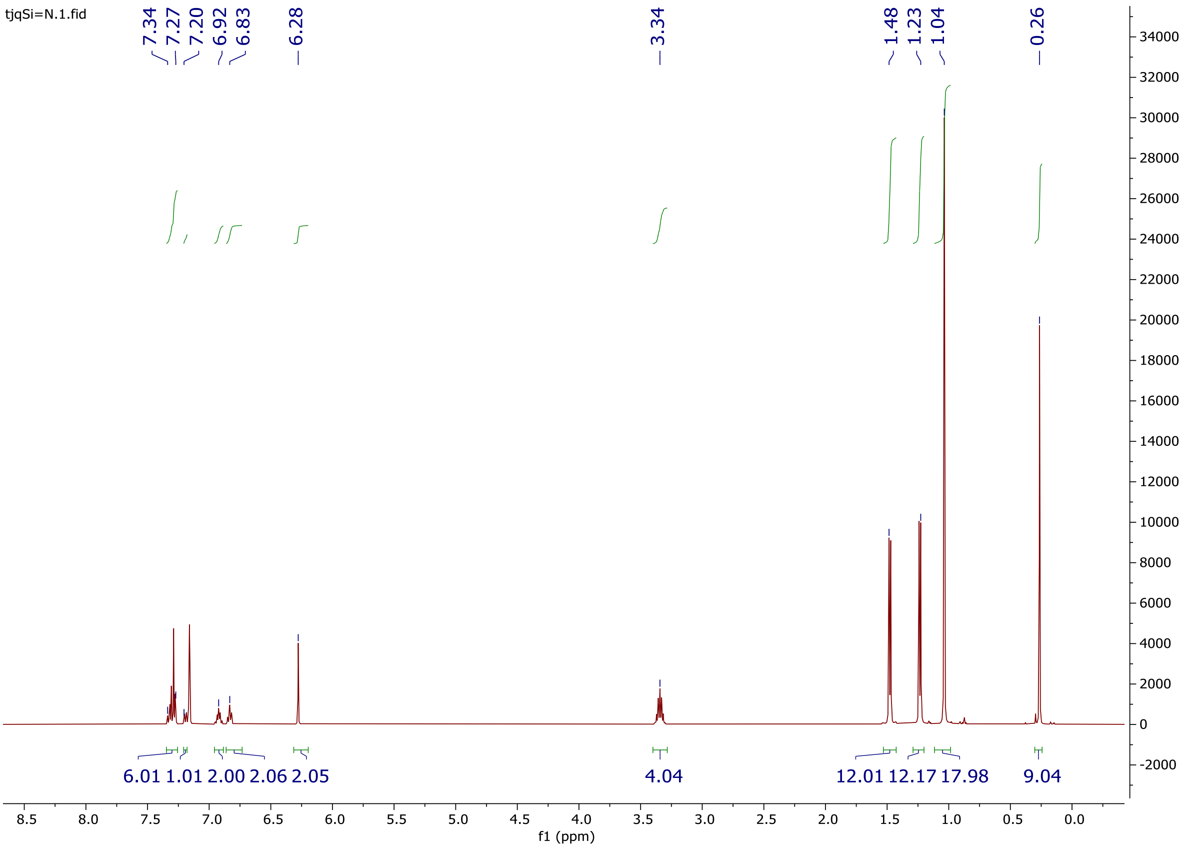

Figure S1: ^{1}H NMR spectrum of **2**-Si in C_6D_6 at room temperature.

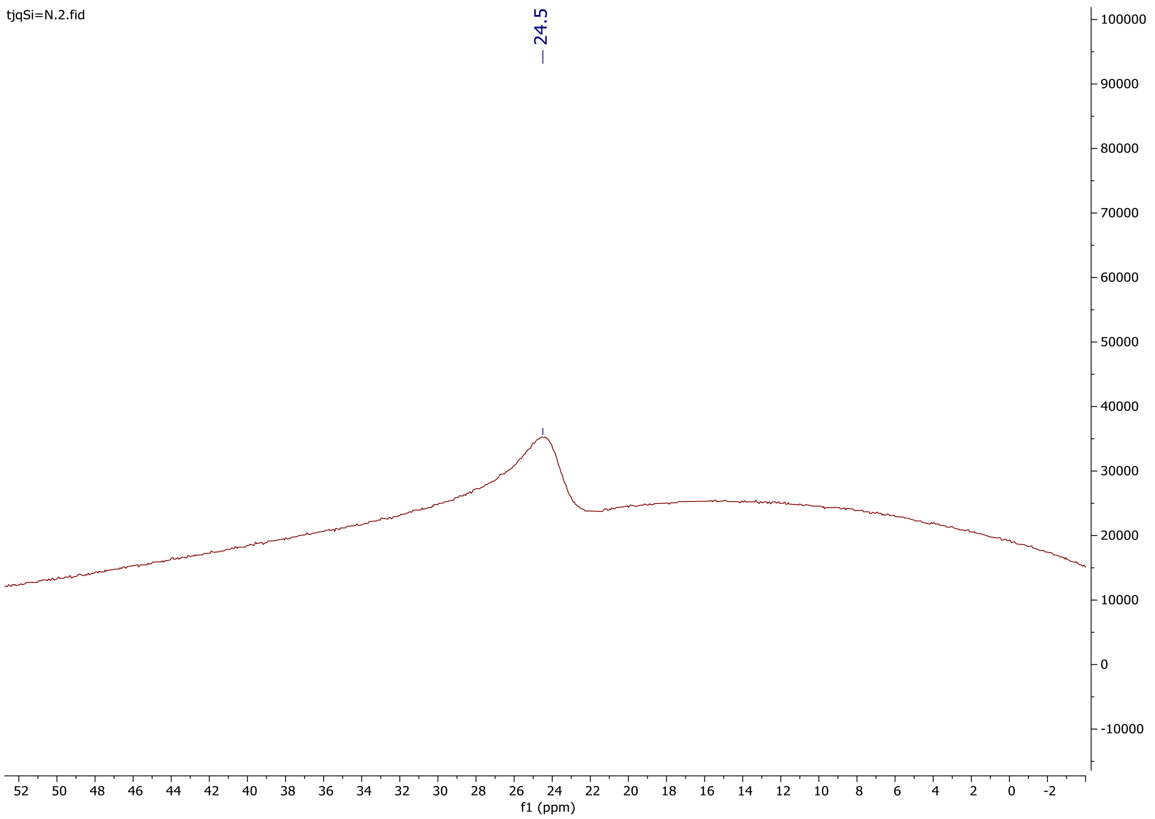

Figure S2: $^{11}B\{^{1}H\}$ NMR spectrum of **2**-Si in C_6D_6 at room temperature.

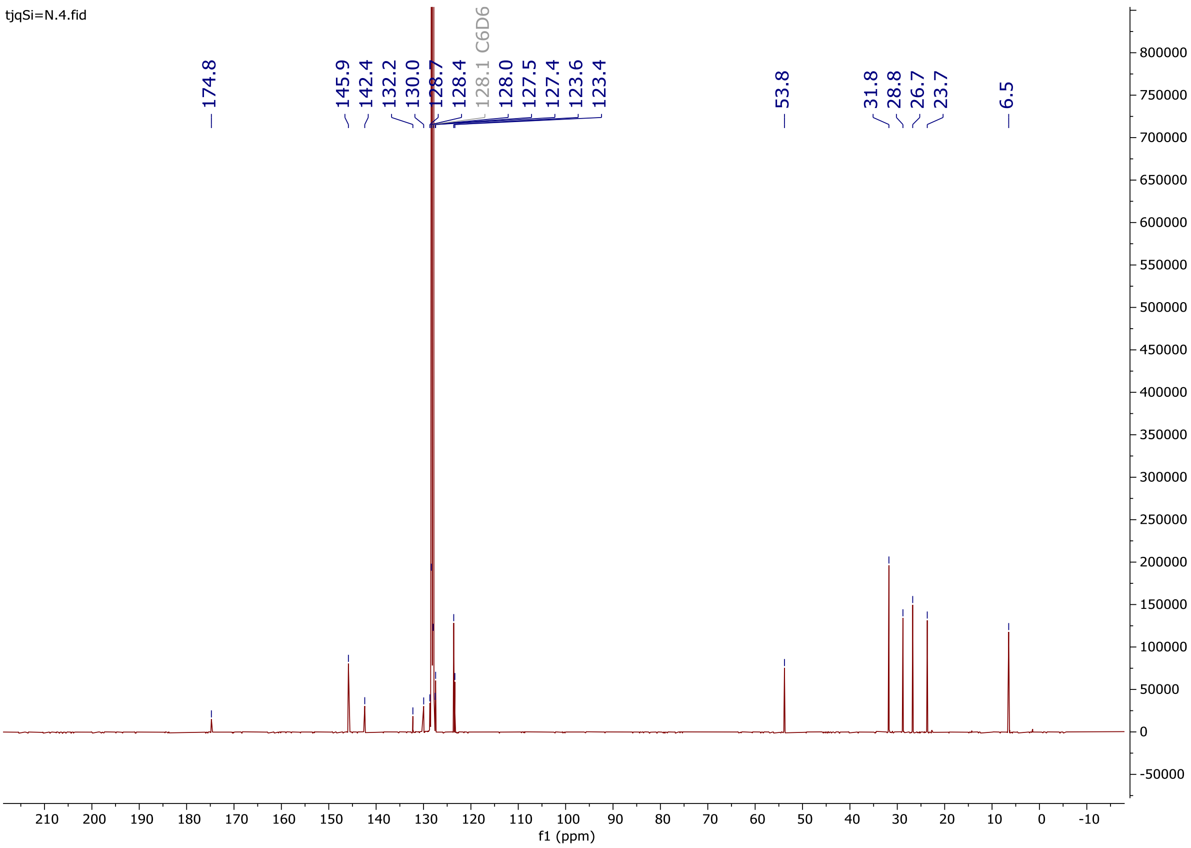

Figure S3: $^{13}C\{^{1}H\}$ NMR spectrum of **2**-Si in C_6D_6 at room temperature.

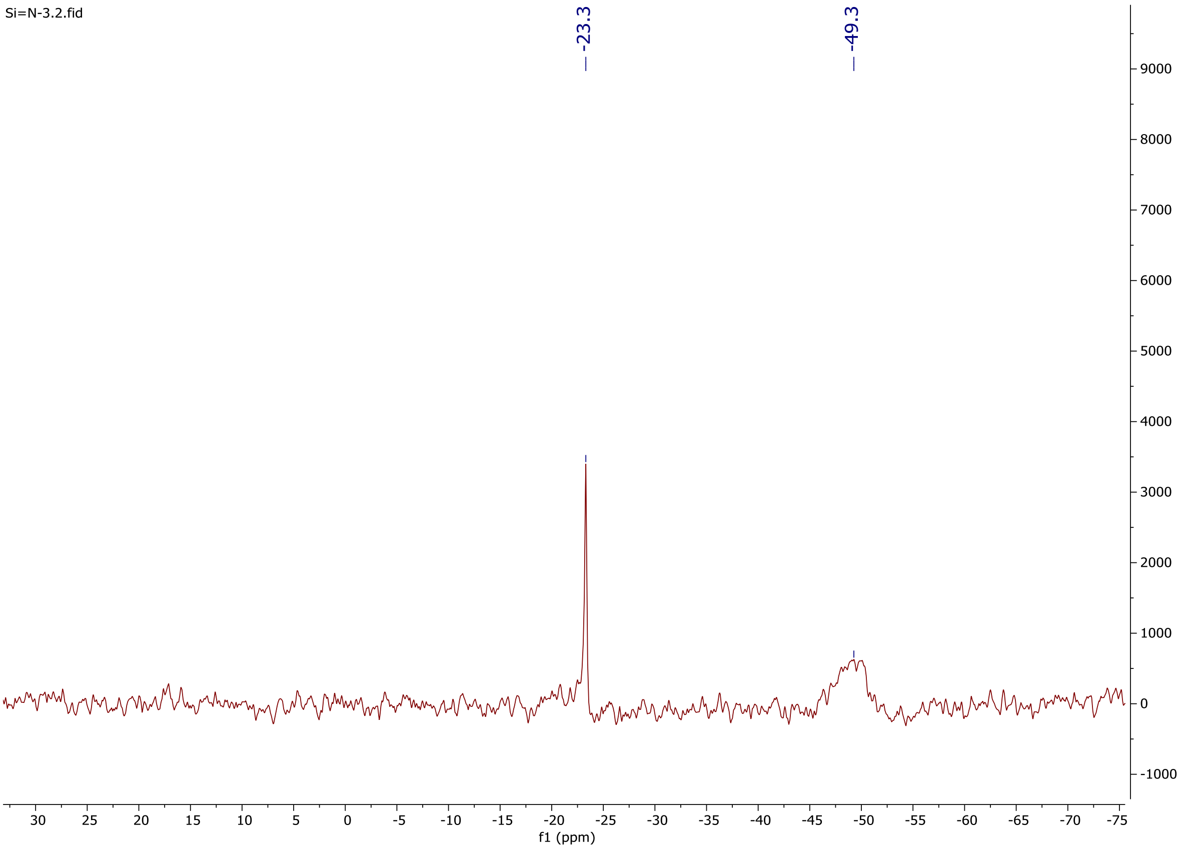

Figure S4: $^{29}Si\{^{1}H\}$ NMR spectrum of **2**-Si in C_6D_6 at room temperature.

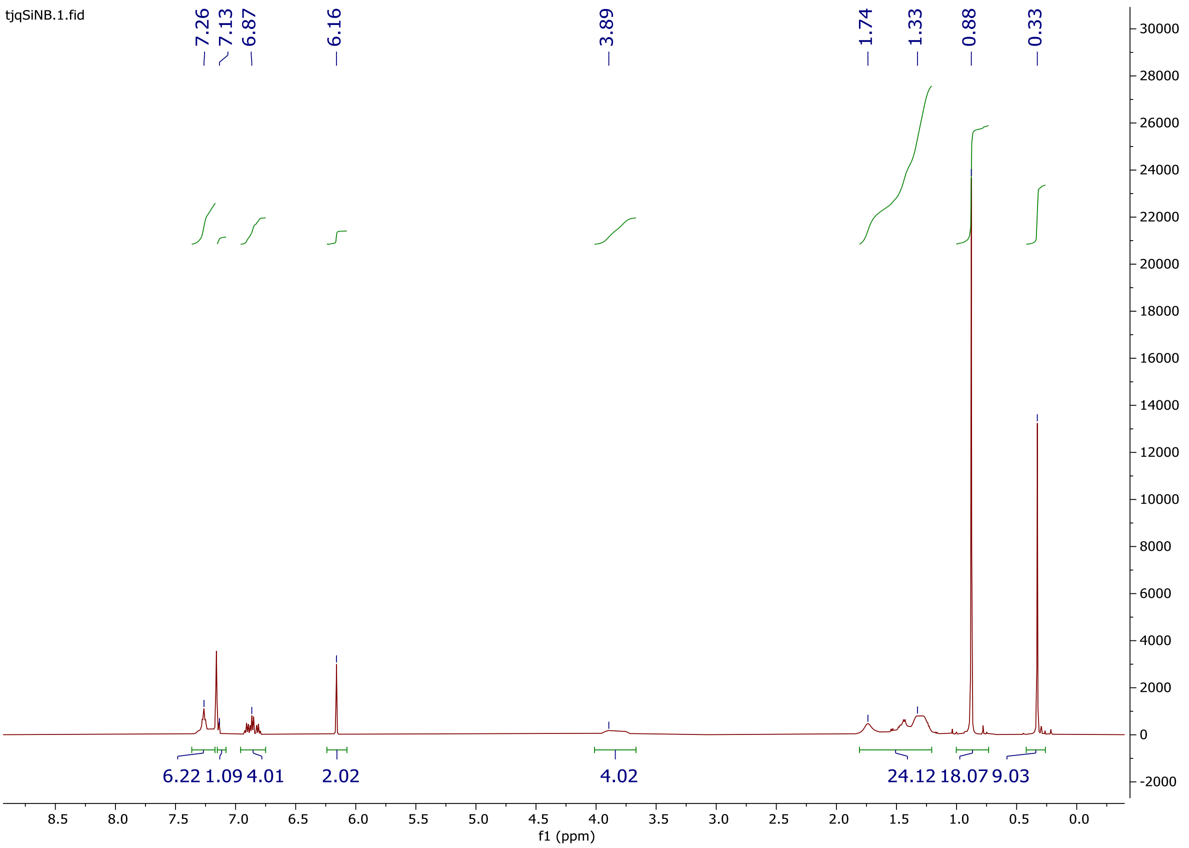

Figure S5: ^1H NMR spectrum of **3**-Si in C_6D_6 at room temperature.

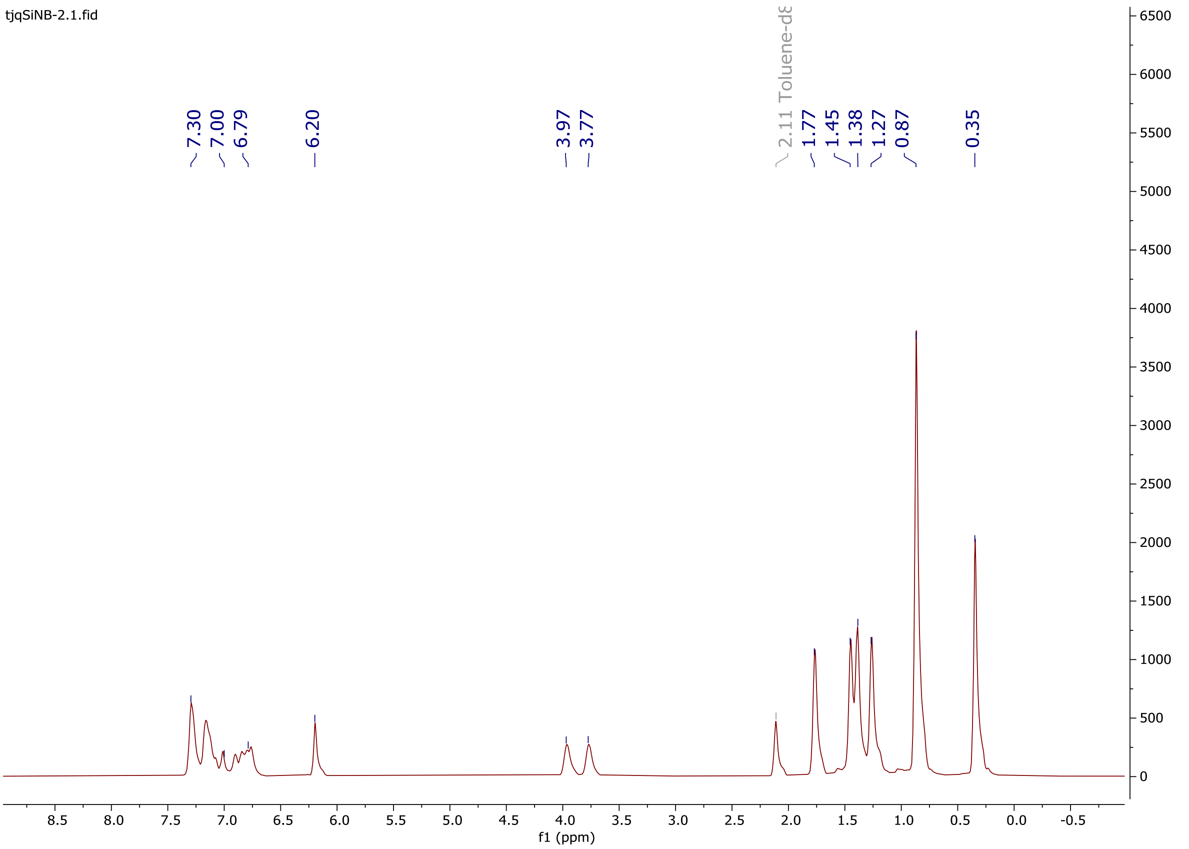

Figure S6: ^1H NMR spectrum of **3**-Si in d_8-toluene at 243 K.

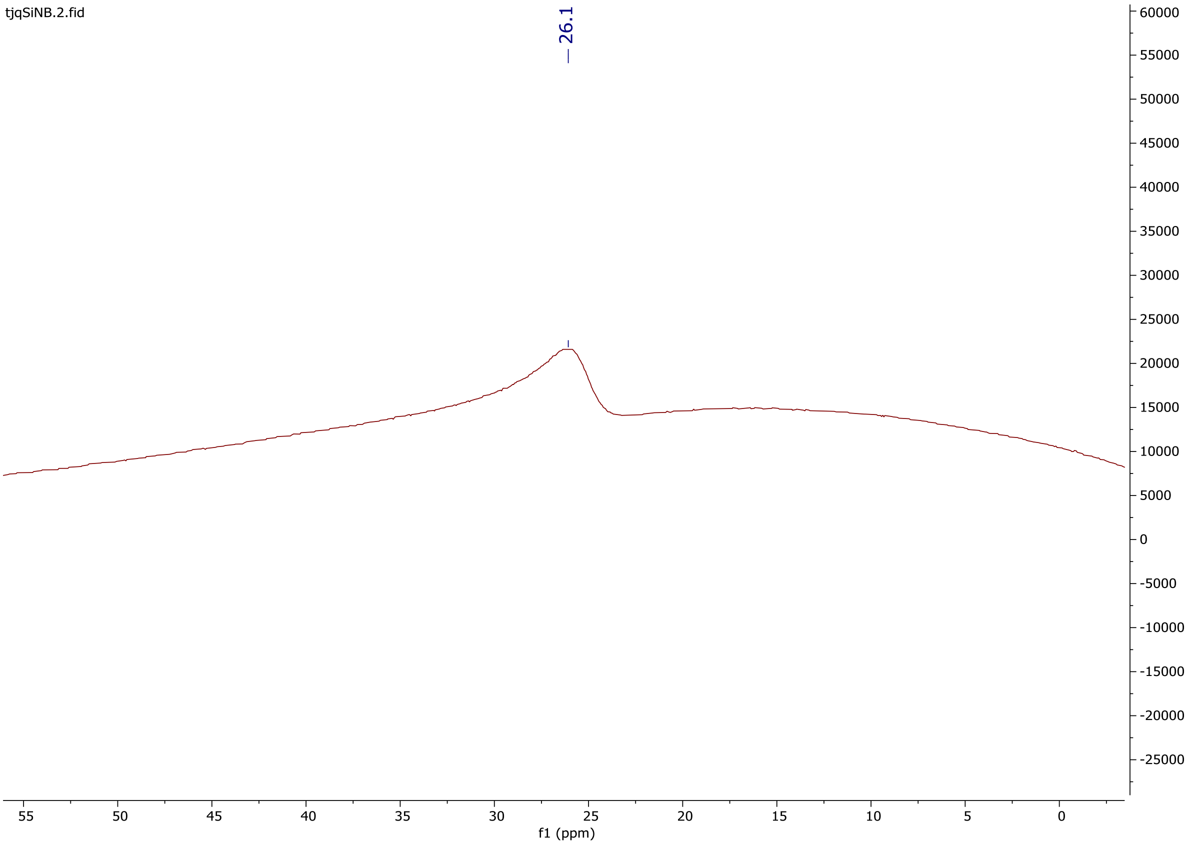

Figure S7: $^{11}B\{^1H\}$ NMR spectrum of **3**-Si in C_6D_6 at room temperature.

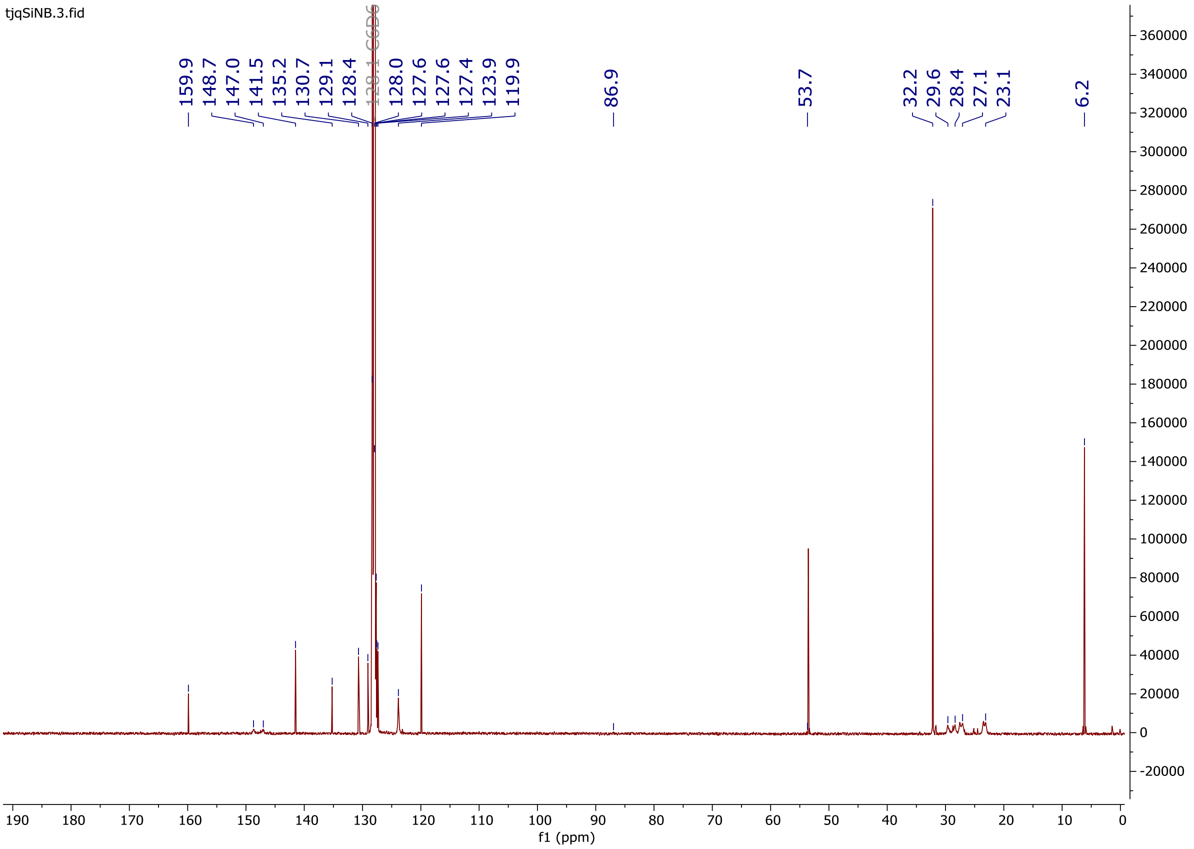

Figure S8: $^{13}C\{^1H\}$ NMR spectrum of **3**-Si in C_6D_6 at room temperature.

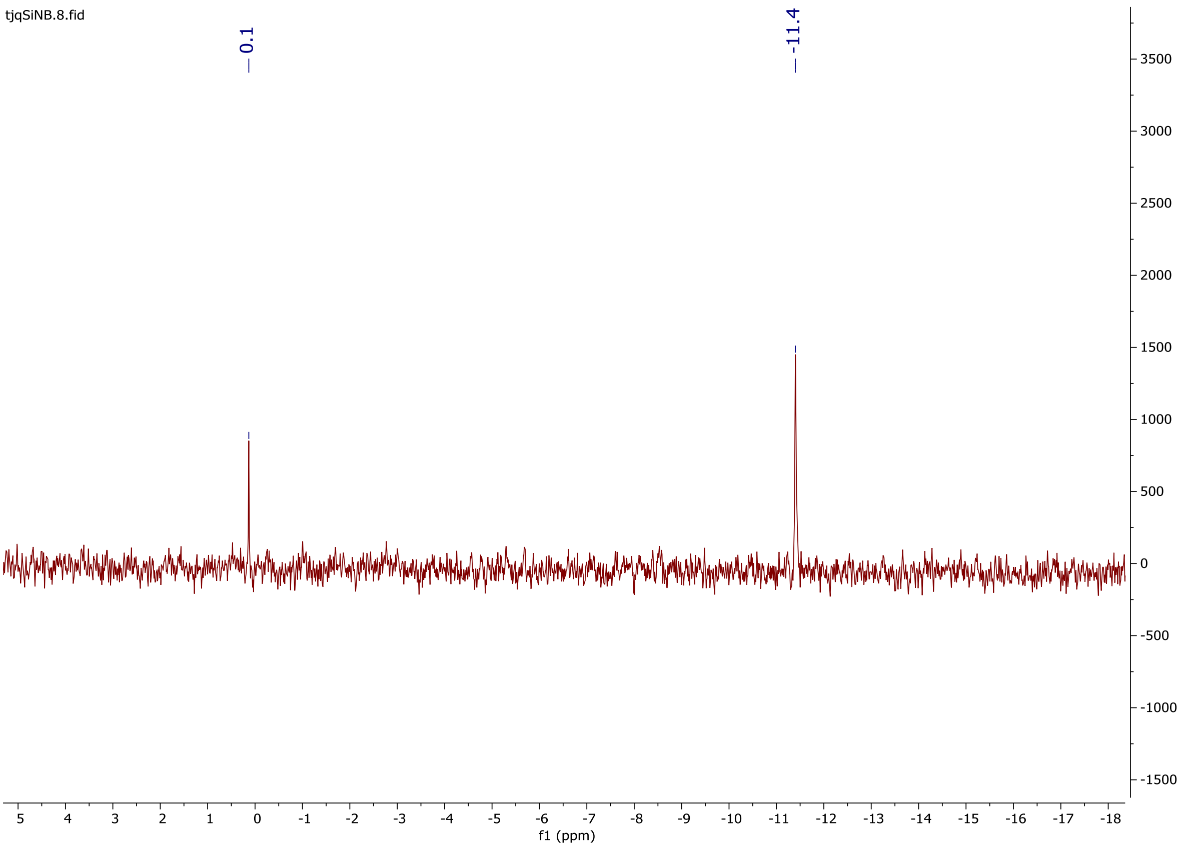

Figure S9: $^{29}Si\{^{1}H\}$ NMR spectrum of **3**-Si in C_6D_6 at room temperature.

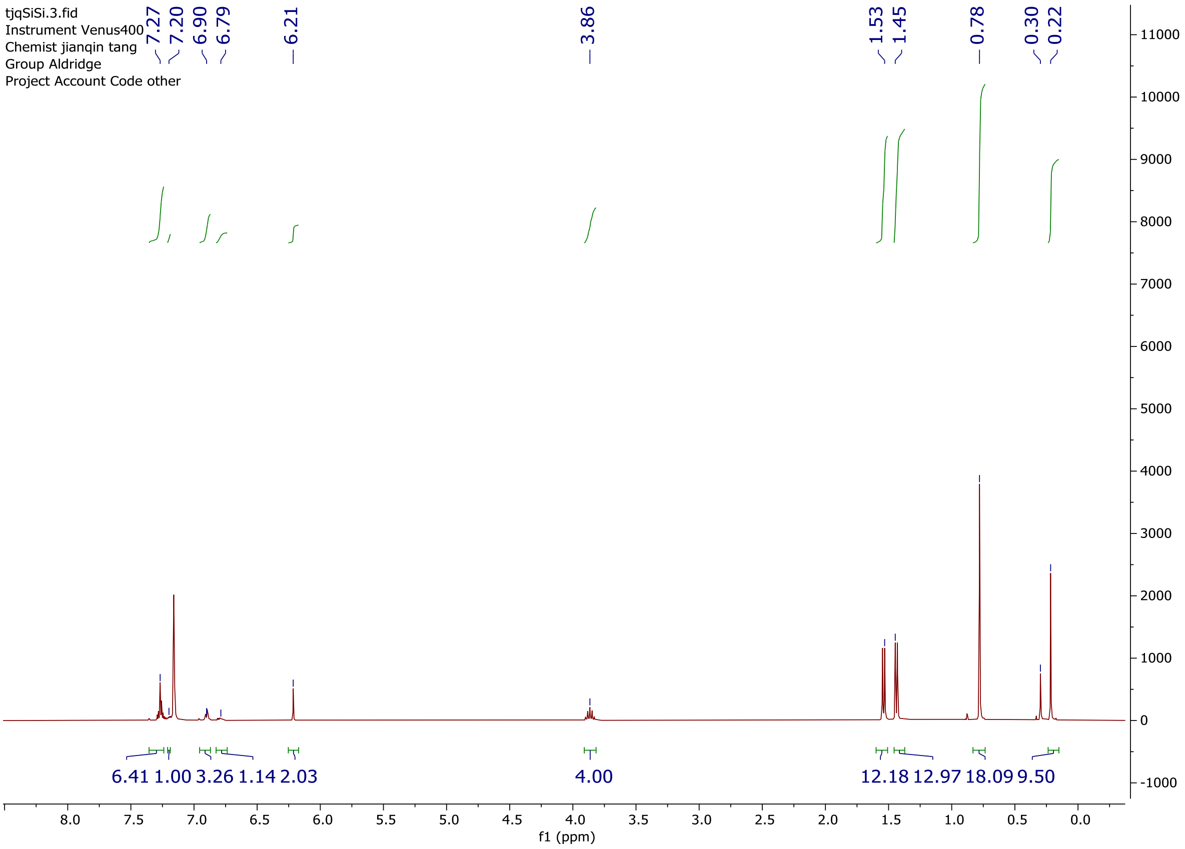

Figure S10: ^{1}H NMR spectrum of **4** in C_6D_6 at room temperature.

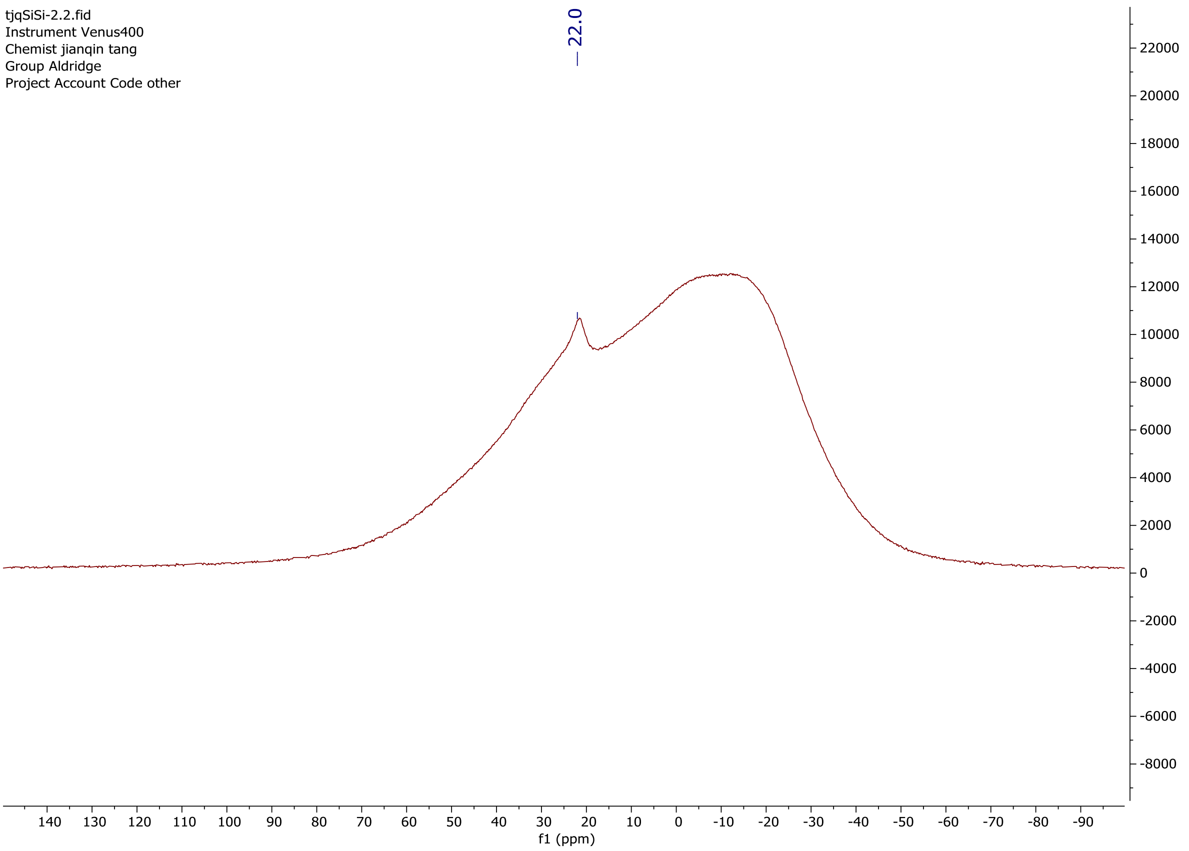

Figure S11: $^{11}B\{^{1}H\}$ NMR spectrum of **4** in C_6D_6 at room temperature.

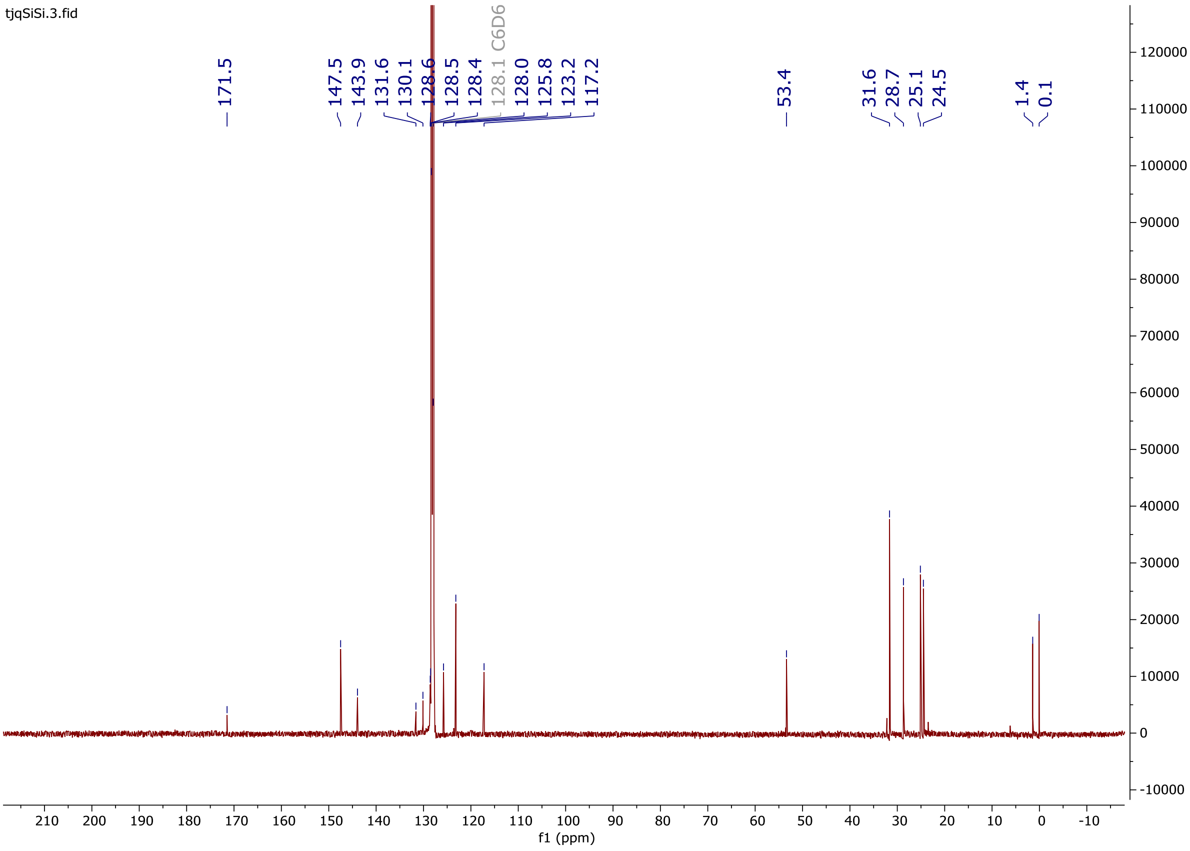

Figure 12: $^{13}C\{^{1}H\}$ NMR spectrum of **4** in C_6D_6 at room temperature.

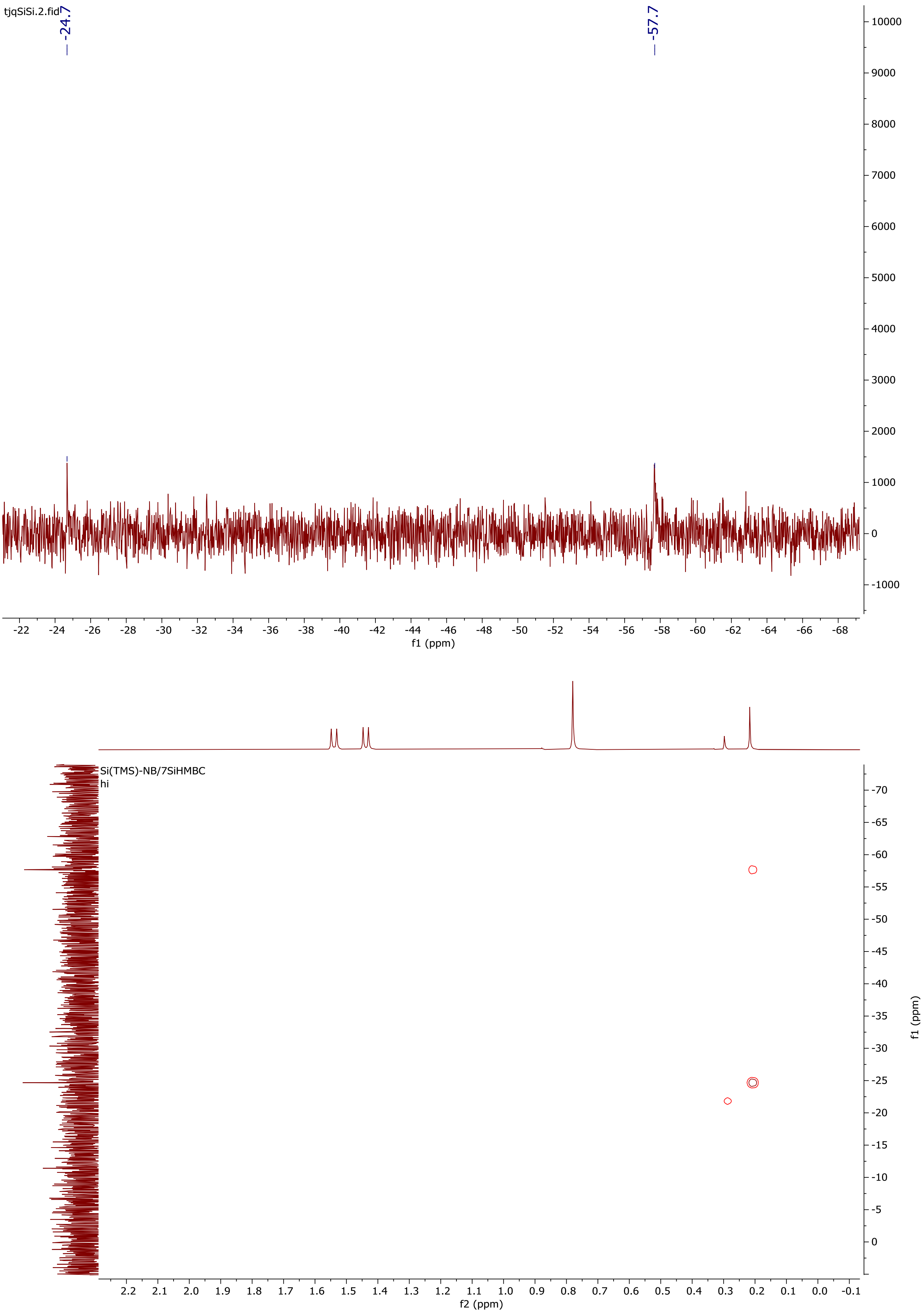

Figure S13: (upper)$^{29}Si\{^{1}H\}$ NMR spectrum of **4** in C_6D_6 at room temperature; (lower) $^{1}H/^{29}Si$ HMBC NMR spectrum of **4** in C_6D_6 at room temperature.

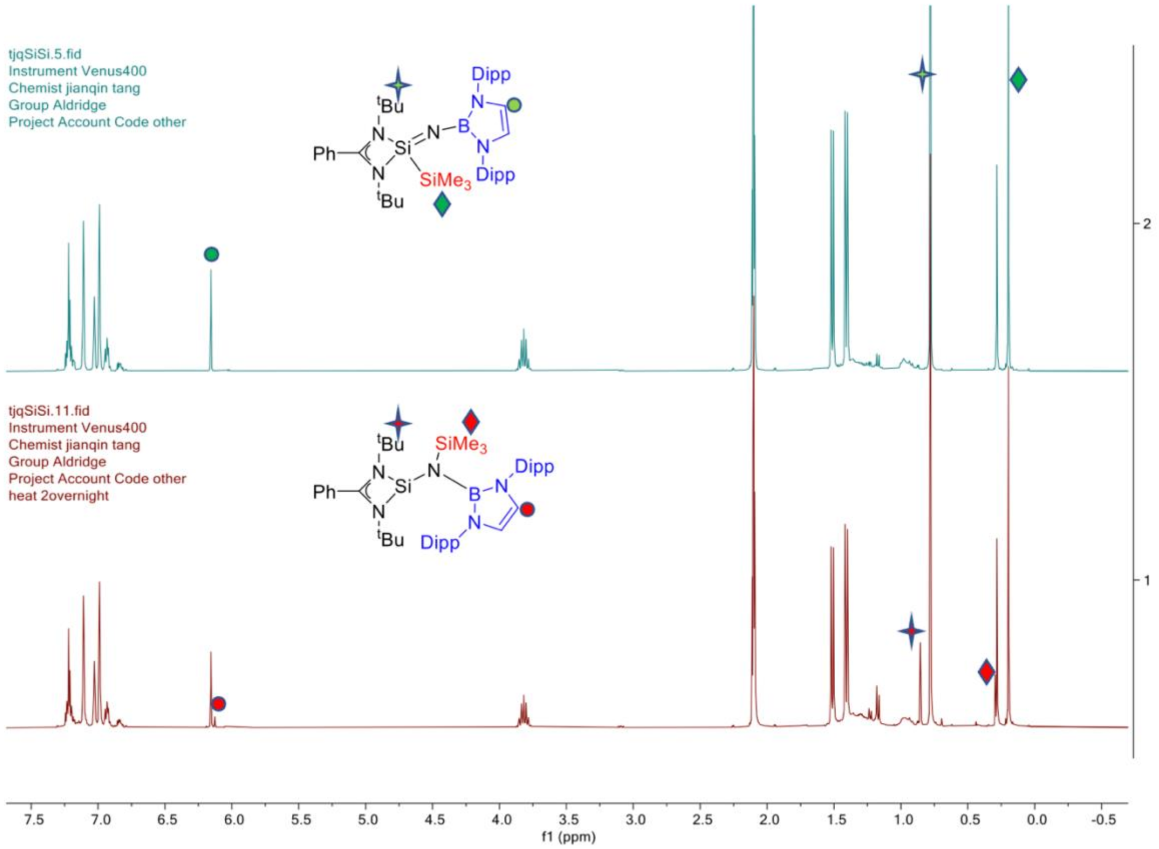

Figure S14: ^{1}H NMR spectra showing the re-equilibration of **4** (upper) into a mixture containing **4** and **3**-Si on heating in C_7D_8 (12 h, 110°C).

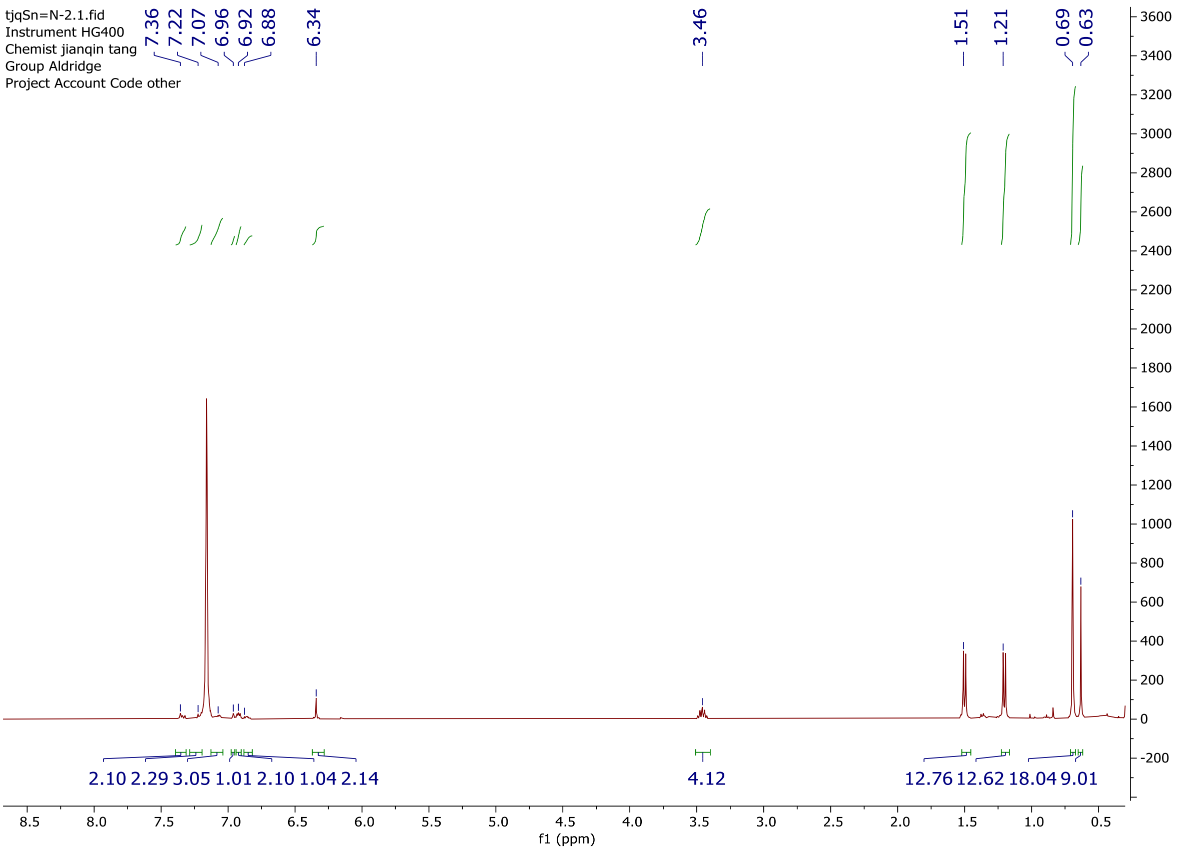

Figure S15: ^{1}H NMR spectrum of **2**-Sn in C_6D_6 at room temperature.

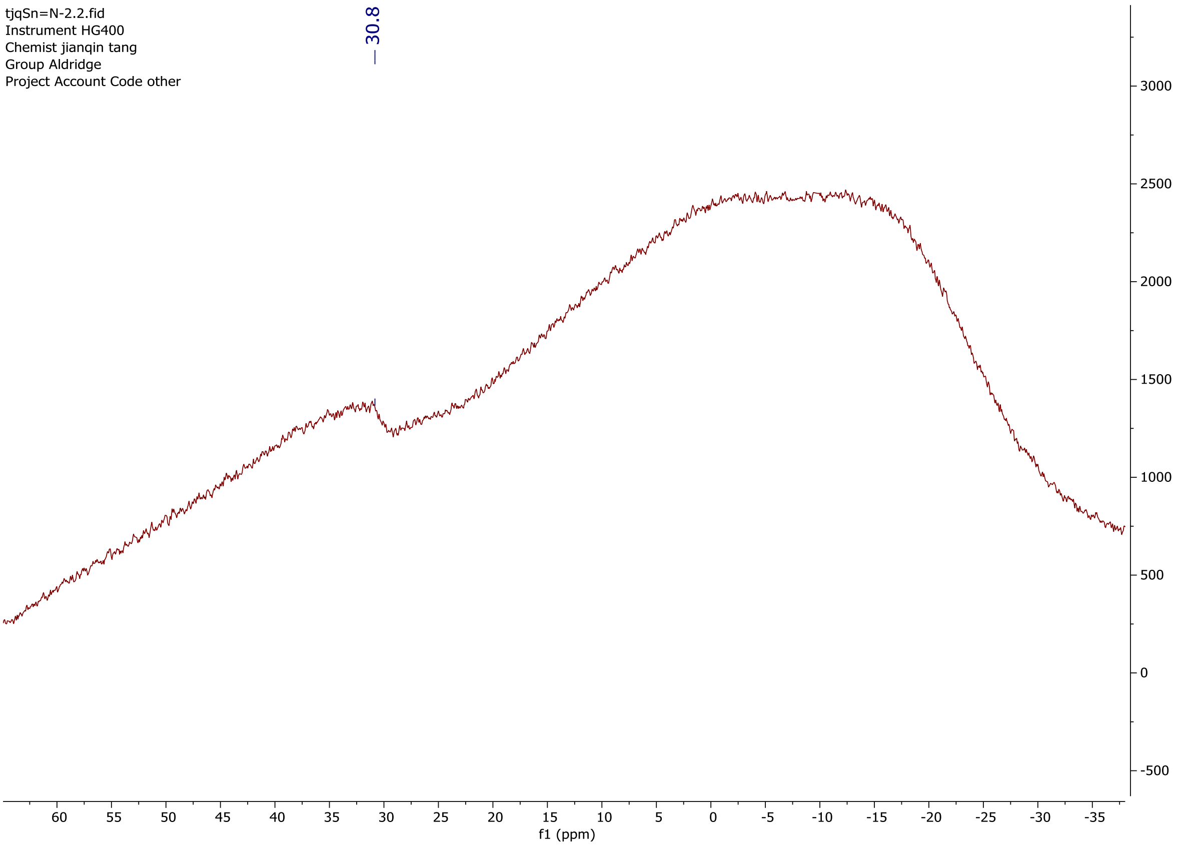

Figure S16: $^{11}B\{^{1}H\}$ NMR spectrum of **2**-Sn in C_6D_6 at room temperature.

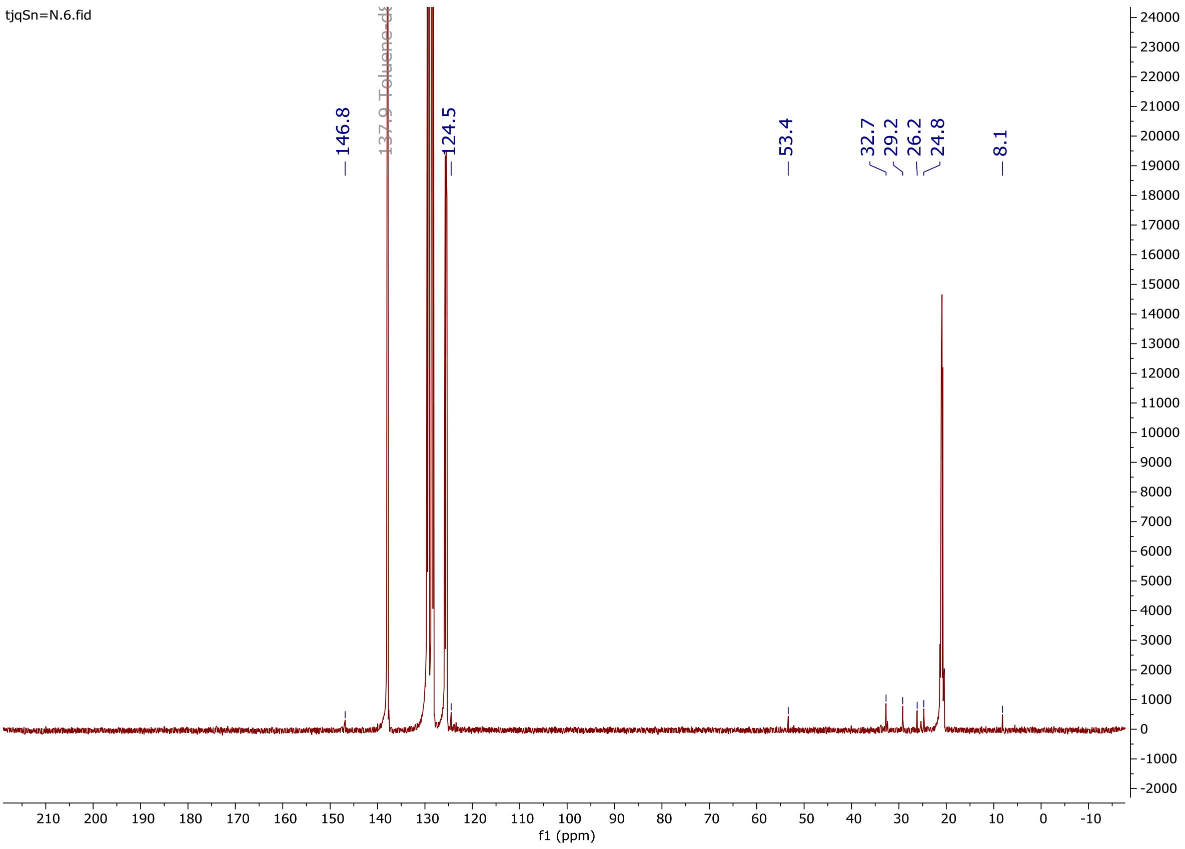

Figure S17: $^{13}C\{^{1}H\}$ NMR spectrum of **2**-Sn in d_8-toluene at 243 K.

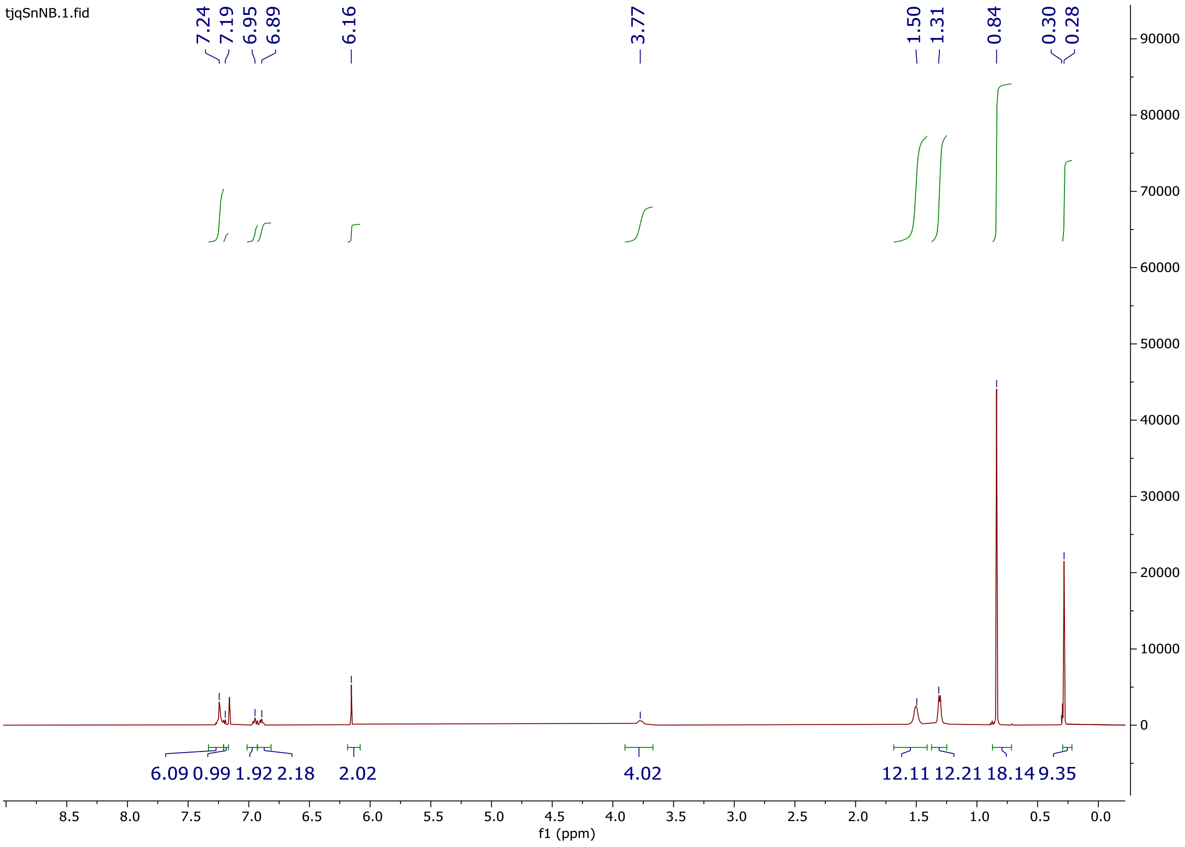

Figure S18: ^{1}H NMR spectrum of **3**-Sn in C_6D_6 at room temperature.

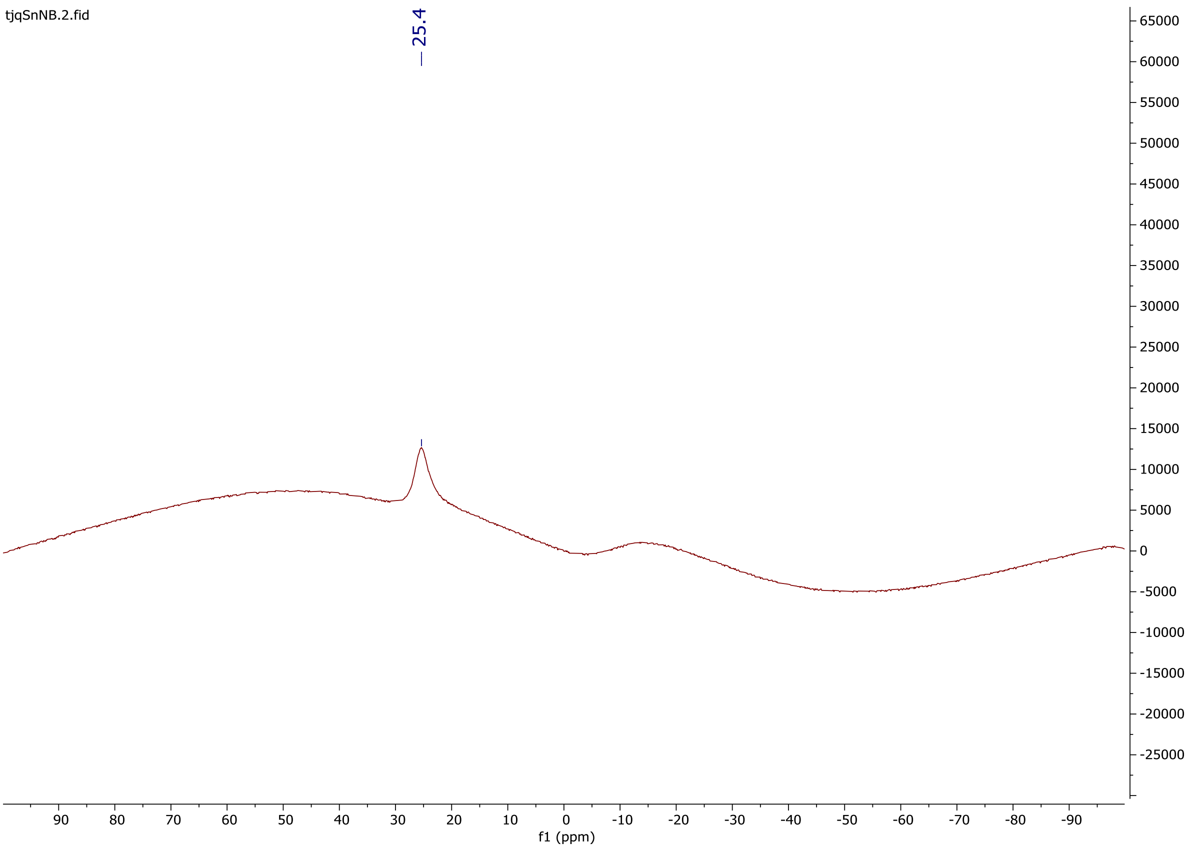

Figure S19: $^{11}B\{^1H\}$ NMR spectrum of **3**-Sn in C_6D_6 at room temperature.

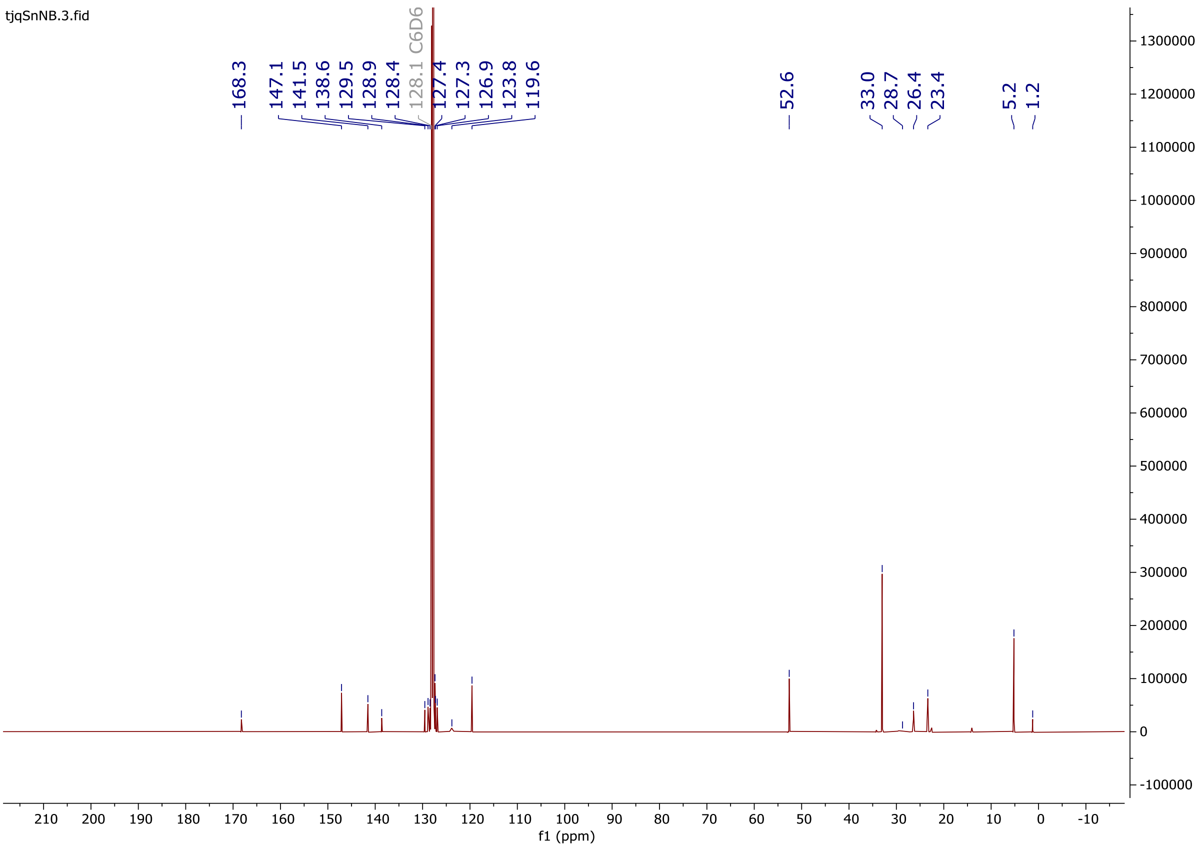

Figure S20: $^{13}C\{^1H\}$ NMR spectrum of **3**-Sn in C_6D_6 at room temperature.

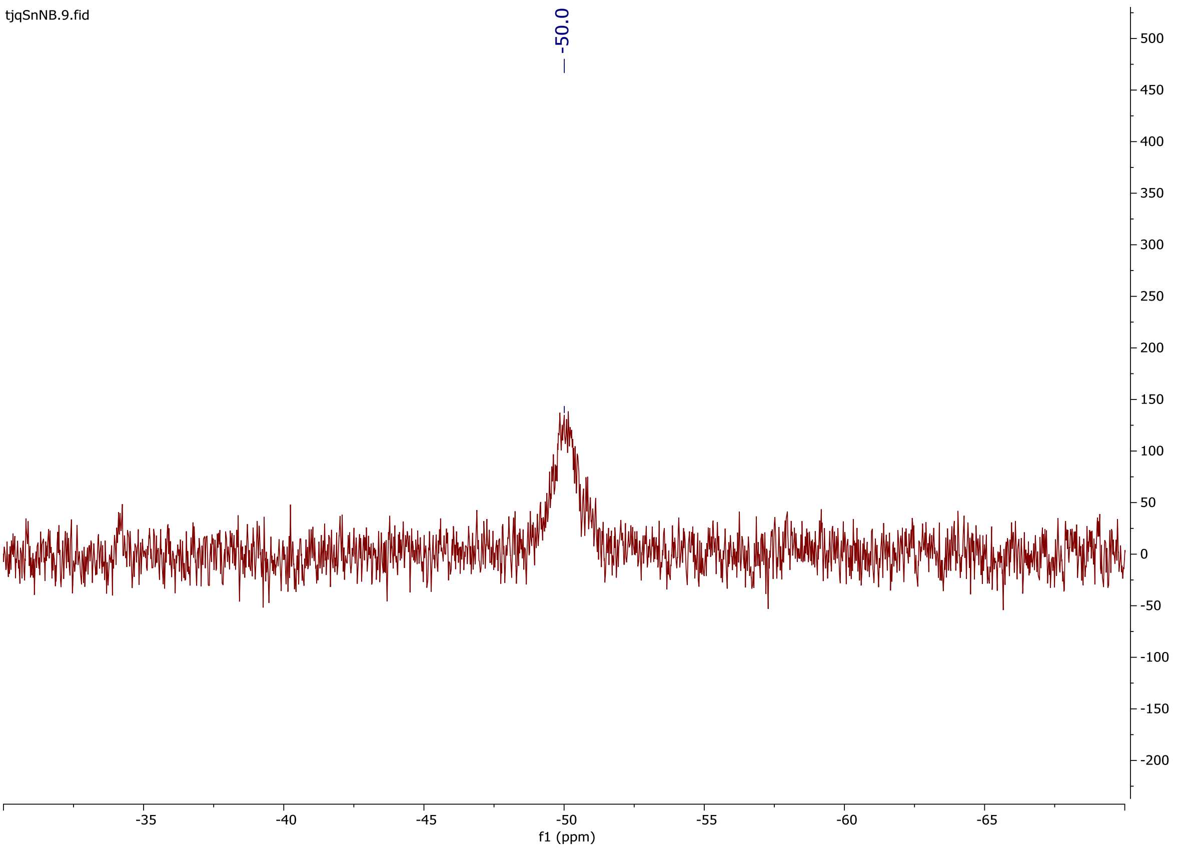

Figure S21: $^{119}Sn\{^{1}H\}$ NMR spectrum of **3**-Sn in C_6D_6 at room temperature.

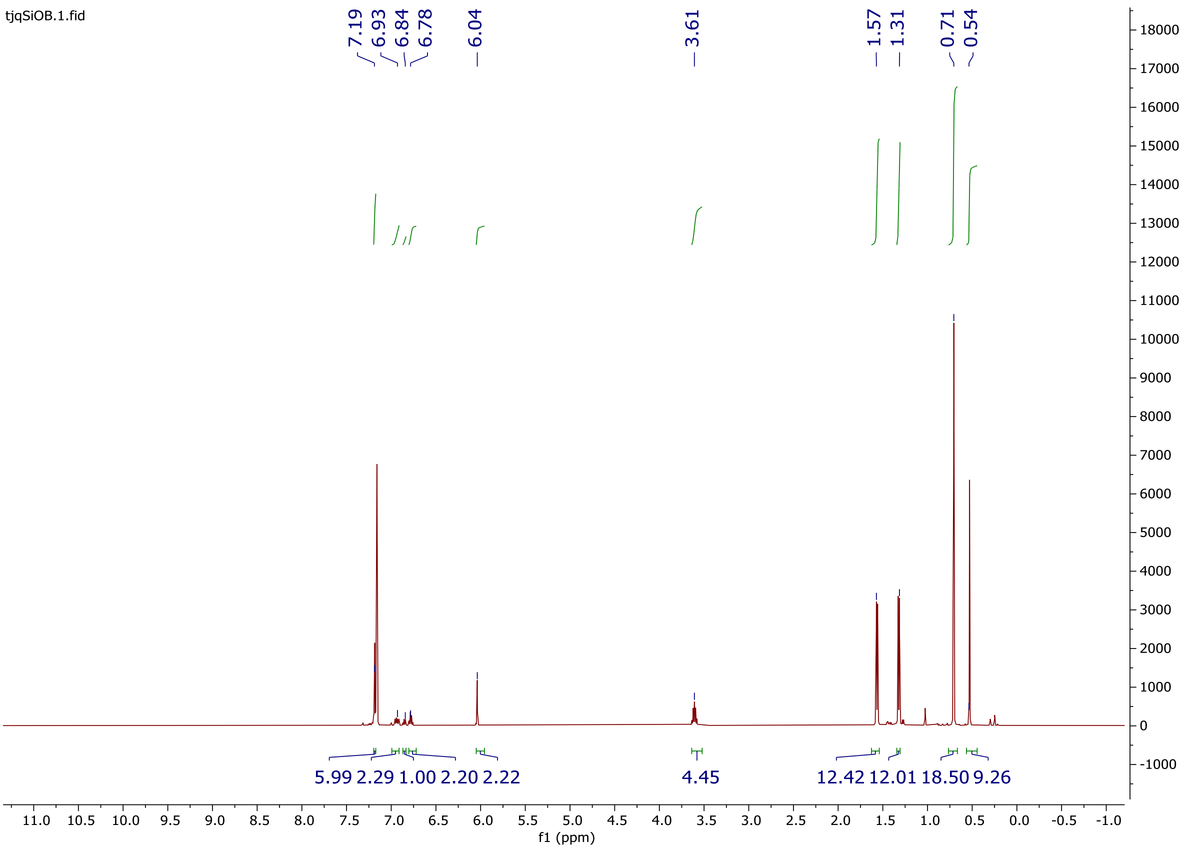

Figure S22: ^{1}H NMR spectrum of **5** in C_6D_6 at room temperature.

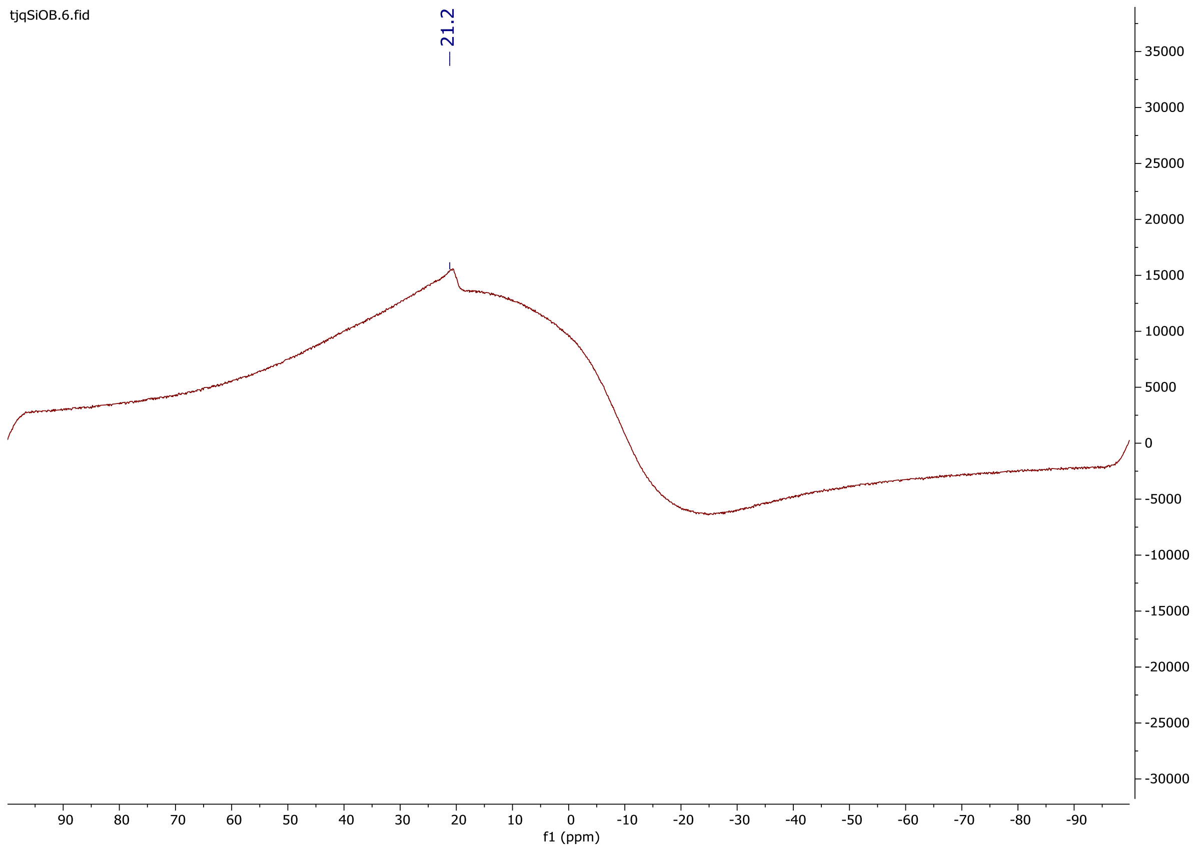

Figure S23: $^{11}B\{^{1}H\}$ NMR spectrum of **5** in C_6D_6 at room temperature.

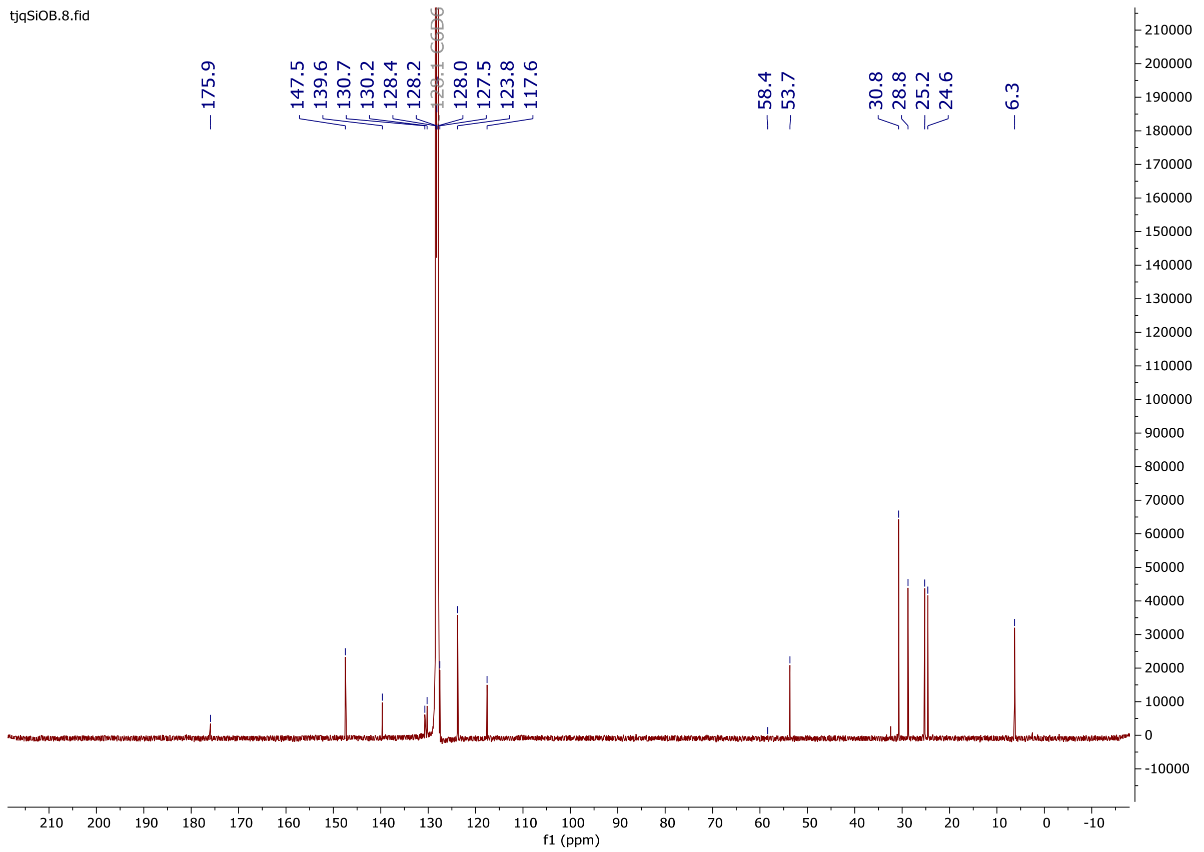

Figure S24: $^{13}C\{^{1}H\}$ NMR spectrum of **5** in C_6D_6 at room temperature.

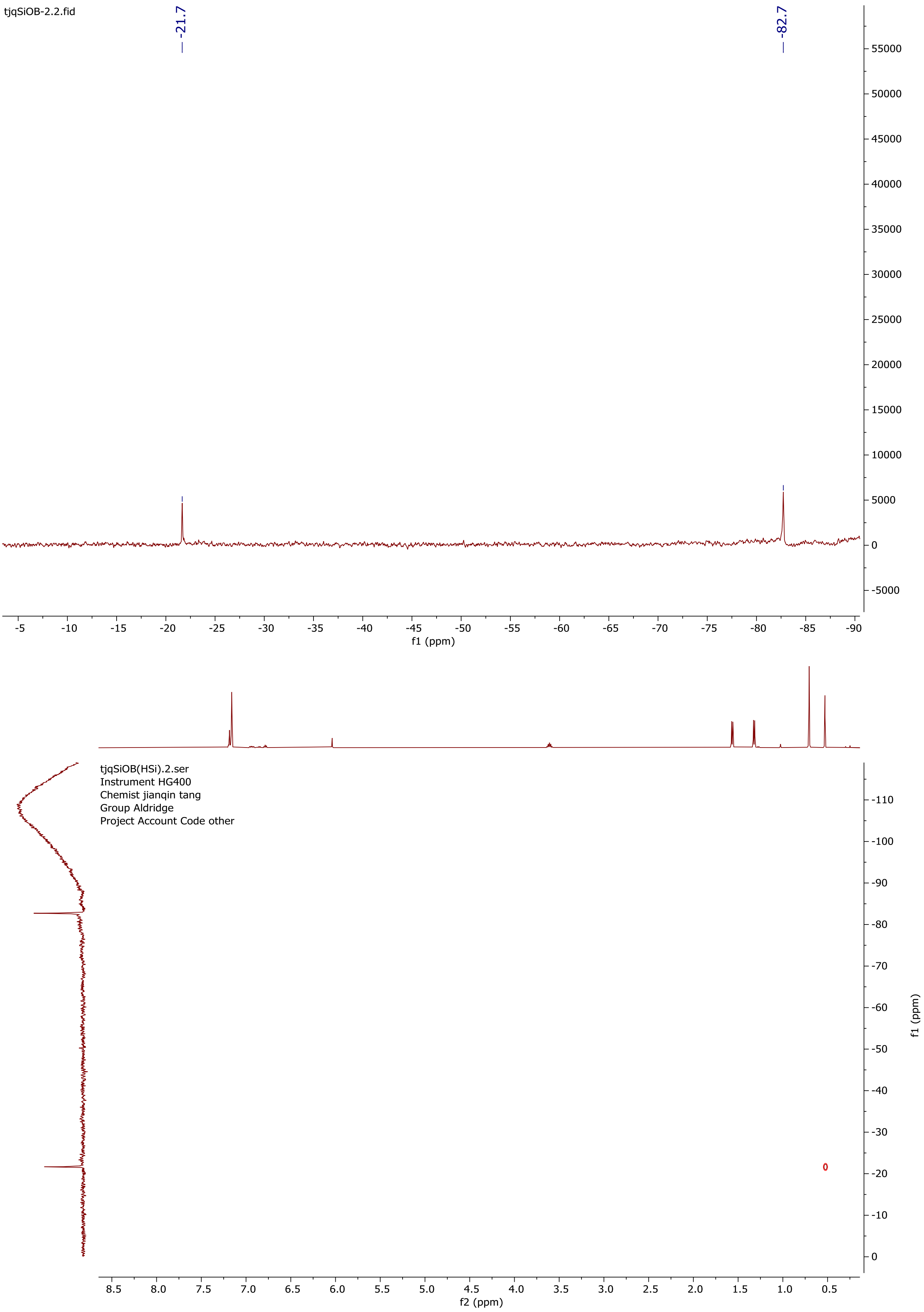

Figure S25: (upper) $^{29}Si\{^{1}H\}$ NMR spectrum of **5** in C_6D_6 at room temperature; (lower) $^{1}H/^{29}Si$ HMBC NMR spectrum of **5** in C_6D_6 at room temperature.

5. Details of X-ray crystallography

Table S1: Selected crystallographic data

Identification code	**2-**Si	**3**-Si
Empirical formula	$C_{44}H_{68}BN_5Si_2$	$C_{44}H_{68}BN_5Si_2$
Formula weight	734.02	734.02
Temperature/K	150.01(11)	150.00(10)
Crystal system	monoclinic	triclinic
Space group	$P2_1/c$	P-1
a/Å	14.19530(10)	12.6777(2)
b/Å	18.78600(10)	13.2202(3)
c/Å	17.02960(10)	16.6109(3)
α/°	90	90.047(2)
β/°	98.2980(10)	110.485(2)
γ/°	90	117.338(2)
Volume/Å^3	4493.79(5)	2272.62(9)
Z	4	2
ρ_{calc}g/cm^3	1.085	1.073
μ/mm^{-1}	0.965	0.955
F(000)	1600	800
Crystal size/mm^3	0.25 × 0.2 × 0.2	0.27 × 0.21 × 0.18
Radiation	CuKα (λ = 1.54184)	Cu Kα (λ = 1.54184)
2Θ range for data collection/°	6.292 to 152.314	7.674 to 153.936
Index ranges	-17 ≤ h ≤ 17, -23 ≤ k ≤ 21, -21 ≤ l ≤ 21	-15 ≤ h ≤ 15, -16 ≤ k ≤ 16, -20 ≤ l ≤ 17
Reflections collected	46619	55072
Independent reflections	9334 [R_{int} = 0.0335, R_{sigma} = 0.0232]	9449 [R_{int} = 0.0414, R_{sigma} = 0.0220]
Data/restraints/parameters	9334/0/486	9449/0/486
Goodness-of-fit on F^2	1.035	1.032
Final R indexes [I>=2σ (I)]	R_1 = 0.0363, wR_2 = 0.0950	R_1 = 0.0377, wR_2 = 0.1009
Final R indexes [all data]	R_1 = 0.0421, wR_2 = 0.0999	R_1 = 0.0438, wR_2 = 0.1072
Largest diff. peak/hole / e Å^{-3}	0.27/-0.29	0.41/-0.27
CCDC ref	2429639	2429643

Identification code	**4**	**5**
Empirical formula	$C_{44}H_{68}BN_5Si_2$	$C_{44}H_{68}BN_5OSi_2$
Formula weight	734.02	750.02
Temperature/K	150.00(10)	150.01(11)
Crystal system	monoclinic	monoclinic
Space group	$P2_1/c$	$P2_1/c$
a/Å	10.6428(4)	17.9575(2)
b/Å	39.9620(9)	10.98470(10)
c/Å	11.8396(5)	23.0147(3)
α/°	90	90
β/°	115.634(5)	91.2430(10)
γ/°	90	90
Volume/Å^3	4539.9(3)	4538.76(9)
Z	4	4
ρ_{calc}g/cm^3	1.074	1.098
μ/mm^{-1}	0.956	0.983
F(000)	1600	1632
Crystal size/mm^3	0.4 × 0.4 × 0.4	0.1 × 0.1 × 0.1
Radiation	Cu Kα (λ = 1.54184)	Cu Kα (λ = 1.54184)
2Θ range for data collection/°	8.574 to 153.402	7.684 to 152.958
Index ranges	-13 ≤ h ≤ 13, -47 ≤ k ≤ 50, -14 ≤ l ≤ 14	-22 ≤ h ≤ 22, -13 ≤ k ≤ 13, -28 ≤ l ≤ 28
Reflections collected	22532	14696
Independent reflections	22532 [R_{int} = ?, R_{sigma} = 0.0384]	14696 [R_{int} = ?, R_{sigma} = 0.0110]
Data/restraints/parameters	22532/1/487	14696/17/572
Goodness-of-fit on F^2	1.049	1.058
Final R indexes [I>=2σ (I)]	R_1 = 0.0687, wR_2 = 0.2049	R_1 = 0.0529, wR_2 = 0.1575
Final R indexes [all data]	R_1 = 0.0918, wR_2 = 0.2148	R_1 = 0.0585, wR_2 = 0.1620
Largest diff. peak/hole / e Å^{-3}	0.70/-0.50	0.36/-0.25
CCDC ref	2429641	2429640

Identification code	**2**-Sn	**3**-Sn
Empirical formula	$C_{44}H_{68}BN_5SiSn$	$C_{44}H_{68}BN_5SiSn$
Formula weight	824.62	824.62
Temperature/K	150.0(2)	150.01(10)
Crystal system	monoclinic	monoclinic
Space group	$P2_1/n$	$P2_1/n$
a/Å	11.93521(6)	10.15370(10)
b/Å	18.56922(7)	20.1864(2)
c/Å	21.25962(8)	21.9443(3)
α/°	90	90
β/°	97.6432(4)	93.3950(10)
γ/°	90	90
Volume/Å^3	4669.86(3)	4489.96(9)
Z	4	4
ρ_{calc}g/cm^3	1.173	1.22
μ/mm^{-1}	4.838	5.031
F(000)	1744	1744
Crystal size/mm^3	0.415 × 0.13 × 0.113	0.4 × 0.4 × 0.4
Radiation	Cu Kα (λ = 1.54184)	Cu Kα (λ = 1.54184)
2Θ range for data collection/°	6.344 to 152.658	8.072 to 152.76
Index ranges	-15 ≤ h ≤ 15, -23 ≤ k ≤ 23, -26 ≤ l ≤ 26	-10 ≤ h ≤ 12, -22 ≤ k ≤ 25, -27 ≤ l ≤ 27
Reflections collected	126615	50920
Independent reflections	9729 [R_{int} = 0.0420, R_{sigma} = 0.0144]	9354 [R_{int} = 0.0374, R_{sigma} = 0.0222]
Data/restraints/parameters	9729/342/508	9354/0/487
Goodness-of-fit on F^2	1.054	1.049
Final R indexes [I>=2σ (I)]	R_1 = 0.0218, wR_2 = 0.0560	R_1 = 0.0299, wR_2 = 0.0777
Final R indexes [all data]	R_1 = 0.0238, wR_2 = 0.0578	R_1 = 0.0307, wR_2 = 0.0786
Largest diff. peak/hole / e Å^{-3}	0.50/-0.65	1.20/-1.28
CCDC ref	2429642	2429644

6. Details of quantum chemical calculations

All computational work reported here was performed using ORCA (Revision 5.0.4).[s2] The meta-generalized-gradient approximation (mGGA) functional R2-SCAN[s3] was employed in conjunction with the Def2-TZVPPm[S4] basis set with the D4 dispersion correction,[s5] and employing the geometrical counterpoise correction gCPs5 (together known as the R2SCAN-3c method),[S4] and solvent of toluene with the conductor-like polarizable continuum model (C-PCM).[s6] The nature of the stationary points (minima) was confirmed by full frequency calculations, and are characterized by zero imaginary frequencies.

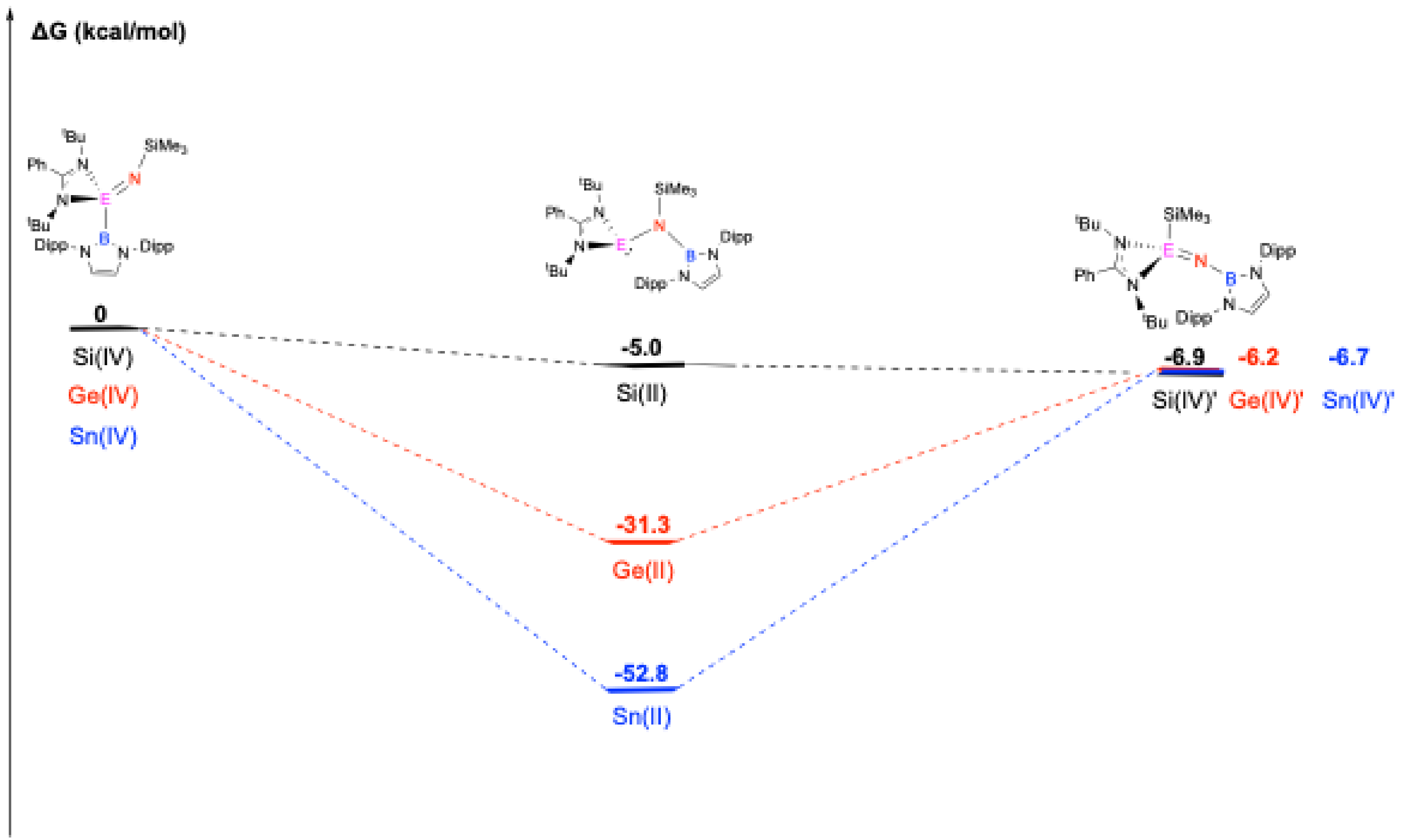

Figure S26. Comparison of the Energies of E(IV)/E(II)/E(IV)’ (E= Si, Ge, Sn)

Coordinates for optimised geometries

2-Si

Si	9.83501621704433	7.12726556504995	4.74790829251278
Si	10.43217363252974	4.03161984343987	4.90570745396059
N	12.49754699288580	8.56886290414215	4.85877760022103
N	10.94126710082566	10.12358072539132	4.27134125018823
N	8.26372574731707	7.23486032605071	3.73594650704918
N	8.35484548449214	7.46151791853296	5.87342381908173
N	10.59631169348783	5.71021406564369	4.88116407674901
C	13.68941154223871	6.94448191380504	6.26526429018277
C	12.19877241318533	10.72984554512525	4.36162403909347
H	12.32700820250180	11.78871554024092	4.18828934482097
C	13.12056226993338	9.80591404735129	4.71284463925158
H	14.18330225748648	9.92799226696506	4.87101434264510
C	9.87439039073017	10.89824332360343	3.70722456262667
C	13.33002499781560	7.40708863601330	4.99085457461926
C	13.19565334425471	7.62165296054540	7.52805133037900
H	12.33297263090007	8.24119365780301	7.25677143213782
C	7.51871511874639	7.41991801173403	4.82959163275458
C	8.84696929039212	11.40178848847401	4.53388561275908
C	14.54942345550083	5.84763315802731	6.34921907171168
H	14.83903291039032	5.46741013189761	7.32473216654097
C	9.93414670640093	11.21545284005982	2.33505271738242
C	15.04126073679670	5.23412022378910	5.20466646541650
H	15.70663583656434	4.37911611394150	5.28949748413952
C	8.92529094033470	12.01798862848621	1.79445001419040
H	8.95443507354901	12.27299574835303	0.73881133395224
C	14.68415448402088	5.71566603296743	3.95223541675840
H	5.07823544558615	5.23410811509309	3.06106322056431
C	8.09937206562864	7.30223489856353	7.31933592293757
C	14.27738024309145	8.55440915568684	8.09662542309528
H	15.17021891555880	7.98067699948114	8.37231196588544
H	13.90969526842774	9.06697512569094	8.99311470213373
H	14.57417200322536	9.31221930552199	7.36450419241939
C	13.82708253228597	6.80913443467681	3.82049453382666
C	8.84880378626481	11.15924399092447	6.03626281521986
H	8.97767500202452	10.07910178129663	6.18556332818335
C	6.04172972023650	7.55077018539474	4.87533111754322
C	7.89220173417037	7.20532503879776	2.30980727146925
C	12.74387073000666	6.61686336585293	8.59477847084258
H	12.04161000363244	5.88562205493570	8.18113449419906
H	12.25198795377706	7.14287800390202	9.42047238175020
H	13.59346792965808	6.06846999566946	9.01595426314946
C	7.84976685718284	12.17679340386670	3.93703722044350
H	7.03864675024194	12.56961534295926	4.54002986449793
C	13.47603686358583	7.32480200025827	2.43747366294538
H	12.68861376547661	8.07784471749127	2.54739719705092
C	11.05876088100230	10.74388328254542	1.42784141765120
H	11.64208467477715	9.99103940373179	1.96825564999875
C	7.88464444500176	12.48261199845185	2.58113600491659
H	7.10116152929557	13.09562230315489	2.14400449611654
C	10.54348481809642	10.09668812223802	0.13443007298755
H	9.82593356665253	9.29872976216928	0.33837456096133
H	11.38392898920079	9.66768141291081	-0.42254102573414
H	10.05860419589857	10.83127025630928	-0.51768900917053
B	11.07724393918379	8.71121927848872	4.59269698733478
C	7.55380197519138	11.58789788122178	6.73045725498474
H	7.60118864760067	11.30948828870796	7.78812037045309
H	6.66305379856915	11.12104986308768	6.29811893396012
H	7.42462089015607	12.67578133739440	6.68838832884788
C	5.45472692907827	8.80980281283011	4.75141709078170
H	6.08350433321949	9.68232859351974	4.59274050644153
C	10.02219594012546	11.87794103817646	6.72758410391372
H	9.96563748481395	12.95688876881234	6.54035187515412
H	10.99268385914005	11.51736754468197	6.38375856908140
H	9.96621637098847	11.71910331416531	7.81050705150490
C	5.24490894226678	6.42028310085315	5.06275237774260

H	5.70744451228657	5.43968043498826	5.13694403302944
C	9.17654191441242	6.81497839586185	1.57082252949466
H	9.98307708365320	7.52611086905987	1.78595239768844
H	9.00284141801608	6.80615839461109	0.49050604684766
H	9.51157208569285	5.82056204439808	1.88428845666321
C	7.40693960354092	8.58296792932610	1.84256008558376
H	6.44235579818829	8.83229976117915	2.29375987237728
H	7.28025388443984	8.58475097479913	0.75412869299302
H	8.12786959954660	9.35916510901237	2.11162396915482
C	7.92161070266849	5.81292452905285	7.64900481660304
H	7.03324780237919	5.40772868073992	7.15430997893954
H	7.80013847380032	5.68281533071202	8.73005368654905
H	8.79554609754862	5.24381684493955	7.32072362030305
C	11.11583989871530	3.31792544080463	6.52151326656376
H	12.16176227533622	3.62706853158414	6.64456074996096
H	11.08305530627354	2.22137262048155	6.52485743989226
H	10.56140309325849	3.67432919045867	7.39792989008079
C	9.35454523850496	7.81909233884190	8.03017960156105
H	10.23971781944508	7.29416359696110	7.65963093571824
H	9.27507727080212	7.63639149512899	9.10623090945214
H	9.49227638395802	8.89371115767751	7.86990516021600
C	11.42909608230217	3.21883294413080	3.51623458074991
H	11.03824885349623	3.49561721471811	2.52927920479185
H	11.39821628796276	2.12468988944146	3.59366882910255
H	12.47869065661416	3.53430141697641	3.55979766911918
C	6.87774371636694	8.08831381708356	7.80556154823593
H	6.91942249383861	9.12632816319024	7.46895209013290
H	6.87175850295934	8.08003784582231	8.90066643219799
H	5.93891485927054	7.64822664080839	7.46142196682437
C	6.81437906058762	6.15055675702118	2.02510106156496
H	7.10828947747890	5.17521101410064	2.42468367469321
H	6.68521242636789	6.05665400756617	0.94155249649945
H	5.84960733824693	6.43006677253457	2.45564326821244
C	3.86317506211912	6.55564115578756	5.14008717297407
H	3.24401983019143	5.67534245715342	5.28570725384663
C	11.99778354048136	11.90926993391708	1.07359220405523
H	11.46174282514990	12.66865362935640	0.49227496909579
H	12.83622434482091	11.54707827603946	0.46801844852292
H	12.40429684776851	12.39259912029650	1.96611634296610
C	4.07231275723179	8.93911372232488	4.83012491790277
H	3.61742801473272	9.92082018285606	4.73502913802768
C	14.68719724612630	8.01079927276237	1.78897472587774
H	15.50599110650188	7.29665928098661	1.64218314842767
H	15.05839565146681	8.82919063763455	2.41492683843895
H	14.41530464357122	8.42201865704885	0.80959453528610
C	12.92164943422815	6.21869717082773	1.53228488672027
H	12.58824766750234	6.64566267417797	0.57902192006217
H	12.07160040585266	5.72058864052421	2.00716100211566
H	13.68011727617487	5.45999807514567	1.30929037332001
C	8.65342773817836	3.39589470532953	4.69538323939299
H	7.97534720139012	3.83293483163415	5.43729715894249
H	8.60159022959740	2.30387571456448	4.79129841619892
H	8.27409913417873	3.65877124907560	3.70046446307976
C	3.27570567376553	7.81401021953943	5.02938101336645
H	2.19626410036353	7.91678534205583	5.09283173237194

TS1

Si	-0.10006004931938	5.90547007626353	3.93073004378097
Si	-1.39653145740663	7.84260837529335	1.72696770668656
N	-2.23302391381736	7.21697257841236	5.90925415386249
N	1.56984172886524	5.25918894782547	3.37849304317232
N	-0.26160093028883	4.11679665048347	3.35931708214660
N	-0.23218367863164	8.37184022219764	5.84658184042633
N	-0.87806998799576	7.19886997574348	3.24109997571284
C	2.88418318958122	5.78704712981385	2.93149868901823
C	1.06392620902866	4.02991630497720	3.19101130697515
C	-2.23938773849126	8.25575953238182	6.86264234960227
H	-3.10255544317445	8.43113543039928	7.49100923882171

C	1.85621746778823	2.78795525693590	2.99929661770623
C	-3.21148834528618	6.18980486366772	6.07625207890447
C	1.14203630028940	8.74892933324820	5.86198341695286
C	1.57763010814816	9.83125681573584	5.06963805961633
C	2.03545908946286	8.08014445934093	6.72119974802094
C	-1.07275016883688	8.92543028088046	6.82727463867250
H	-0.76235817869566	9.77539428585545	7.41989746970455
C	-2.40684258287263	9.41761610829687	1.95829318091000
H	-1.80002786424625	10.22291546487301	2.38742356136927
H	-3.26724029990150	9.26758745064557	2.61741345562731
H	-2.78261564249392	9.76628164785696	0.98850612786074
C	-4.34419108039282	6.15274042135904	5.23671865020964
C	-1.28275935636521	3.04318849808257	3.36921650088790
C	-3.06820370191797	5.26275414444731	7.12721822371049
C	-4.52812082333296	7.19366752151422	4.15103399559444
H	-3.53173143849874	7.38783677941952	3.73718079001822
C	-1.89219079881489	5.30275523657370	8.08291911711262
H	-1.32745708296833	6.21427381024434	7.87367361887026
C	2.83777210386819	7.29814634454797	3.14436996590995
H	1.99006060462755	7.74172495088844	2.61354352996613
H	3.76174536747867	7.75043695479108	2.77171971887386
H	2.74448885038858	7.53870124614439	4.20541643026970
C	3.36364477356406	8.51303269028331	6.77800453729738
H	4.06358165301762	8.00617848742539	7.43800955345772
C	2.90761630306690	10.24182189331701	5.17187689743037
H	3.26044551547522	11.07552475073269	4.57217113750092
B	-0.92648996840574	7.23290994424114	5.20504997923233
C	4.05346343352414	5.20490957928922	3.73538403082628
H	3.91829479827690	5.38712974101085	4.80538399941924
H	4.97954786560159	5.69762833172918	3.41991584842879
H	4.16946156217918	4.13090674119998	3.56959686814867
C	0.62264371257114	10.52569318695297	4.11896231759114
H	-0.10683836357302	9.76627619376943	3.81436741820127
C	0.03778045518891	8.27673315705424	0.57572355644679
H	0.70437026214278	9.02886260100726	1.00834284247618
H	-0.35247314108830	8.67855792677365	-0.36808912549986
H	0.63586030835611	7.39043764221846	0.33569204688235
C	1.99758386118634	2.18567123660502	1.74930293428824
H	1.53336643875461	2.63392166546105	0.87572723678720
C	3.79668528237702	9.59064714111046	6.01910924953942
H	4.82980854905168	9.92227321331287	6.08230818039726
C	1.59295077825906	6.92762610901226	7.60033903054501
H	0.54512405445061	6.72405030815615	7.36771917218394
C	-2.42866612396524	6.57437246898550	0.77995304307774
H	-1.81552860336801	5.69383836872928	0.55020523382125
H	-2.79501181051445	6.98465390321456	-0.16917901033406
H	-3.29246746689433	6.23644697585041	1.36024384527882
C	-1.42929609962035	2.45053671425114	1.96083526881630
H	-2.27140740328888	1.75056357067657	1.95316213527020
H	-0.53526158964716	1.90154493163238	1.65657940699111
H	-1.63007597370281	3.24035092953331	1.22969604007371
C	-2.59596376389659	3.71653135270675	3.75926784140856
H	-2.81988901970238	4.55155723013663	3.08721548376692
H	-2.55098023732830	4.09899697459045	4.78160878248805
H	-3.41580489138835	2.99489500143499	3.70196659738250
C	2.47076647894620	2.22230566958720	4.11995311610271
H	2.35696757626944	2.69746369557687	5.09118269819126
C	3.08171303965040	5.49181664724297	1.43742730005722
H	3.23313764897980	4.42511024284853	1.25378141151521
H	3.96793100174963	6.02404215862123	1.07660377648217
H	2.21657396806785	5.83189101347982	0.85996955292755
C	-4.04034450472660	4.27080236788537	7.28766372490370
H	-3.93059714949950	3.53729119168400	8.08274872070184
C	-0.96285662207933	1.93494693287652	4.37995515031976
H	-1.82296041061472	1.26092548806919	4.45262120874711
H	-0.77715682796234	2.35945705422366	5.37142015753446
H	-0.09419445380309	1.34293133318911	4.08050710733784
C	-0.95762953007594	4.10609823330075	7.86775310472975

H	-1.49433060560381	3.15742732256444	7.98860773270886
H	-0.13847207372835	4.12587913706351	8.59556678840578
H	-0.52212294582004	4.13054912232307	6.86240386392546
C	-5.29729569812814	5.15493480682989	5.44162944501343
H	-6.17193184005545	5.10475029535835	4.80076728139214
C	-2.35299847149517	5.38318130735074	9.54435538170626
H	-3.03091971682970	6.23010214184586	9.69446547311118
H	-1.48719744857327	5.51604908139288	10.20294194401616
H	-2.87351217073114	4.47108136899215	9.85759832801912
C	1.31405426441520	11.06830847499564	2.86434920168218
H	0.56084843643764	11.35654524392978	2.12269953624084
H	1.97604415854086	10.32329690513958	2.41081741262035
H	1.91061140247630	11.96106176036377	3.08565858145121
C	-5.05832886242453	8.50837817012908	4.74848318275080
H	-5.24330578702535	9.24316583792424	3.95600584870233
H	-4.34725865436729	8.94283542160207	5.45610495054225
H	-6.00562132869028	8.32981180114262	5.27161842261860
C	1.67984583041159	7.29982202395481	9.08661392057246
H	1.27085587301216	6.49161113425092	9.70440774123343
H	1.11152420599773	8.21216409565636	9.29587328963215
H	2.71873128449195	7.46783958243581	9.39437556605224
C	-5.44829140641655	6.74208265265231	3.01441408799470
H	-6.49601200034933	6.70380265750794	3.33517359263630
H	-5.17423473775697	5.75261548823268	2.63245209544009
H	-5.38732128097720	7.45691643322052	2.18658979745574
C	2.74877456027254	1.02101560856165	1.62271629724805
H	2.86141714915281	0.55848297265436	0.64667641755912
C	-5.14501636154700	4.21245373467879	6.45218169688337
H	-5.89386368379712	3.43678683511833	6.58989592767441
C	2.38520619580865	5.64837091924351	7.30533615343559
H	3.46170490509507	5.79422930514402	7.45187135725376
H	2.21818010147503	5.32719486540640	6.27144929813034
H	2.06363766353770	4.83935749927860	7.96989347849108
C	3.21204707851447	1.05459144669106	3.98907724326068
H	3.68221211465647	0.61518691197606	4.86382842740741
C	-0.14184925401495	11.66314775477962	4.81458988040473
H	0.55822322743362	12.41966296595475	5.18983701139956
H	-0.73549326229473	11.29457281820130	5.65479977140925
H	-0.82254200098317	12.15011461521269	4.10615196681002
C	3.35274661556308	0.45250674001335	2.74054277777110
H	3.93542555920111	-0.45844565746575	2.63896796227395

3-Si

Si	0.20716494713045	5.83897296736550	4.07071498256716
Si	-0.27301213214725	7.96438873522510	1.76412008468559
N	-2.10776571444244	8.31028390212296	5.17429080779223
N	1.63457024768622	5.23437815638285	2.92219471494472
N	-0.36297859771299	4.44018645842552	2.89424383212737
N	-0.25557619769806	9.61422158670666	4.87872936249833
N	-0.33981298315435	7.40718287444268	3.43881830999254
C	3.10020552315735	5.27484294998216	3.14207433754676
C	0.89587149839321	4.17027483731699	2.54666265010560
C	-2.25054736003583	9.49733791038019	5.90415264157460
H	-3.12687849945796	9.68719640645541	6.50744353853814
C	1.39816351917353	2.90371936359431	1.95107264673856
C	-3.07160838688705	7.27082154421887	5.36351321363558
C	1.05981013443200	10.15071630336794	4.71322952743558
C	1.24602157763564	11.30545730480932	3.93142936095462
C	2.14026285336568	9.55526376520735	5.40743890830564
C	-1.16237045627656	10.27283613754248	5.72260699021181
H	-0.93806393741057	11.23899785279554	6.15199889214288
C	-1.64334830798132	9.22222035409004	1.45995616666198
H	-2.61404094723141	8.71894531594799	1.41629570416002
H	-1.48057777526768	9.71334180042760	0.49289017069248
H	-1.70132248635445	10.00232621275240	2.22523869027786
C	-4.25621158447556	7.27312994720870	4.60519280436382
C	-1.57422588231532	3.60298344110180	2.79211687704112
C	-2.85392799003319	6.31234669333135	6.37699678790278

C	-4.55559566793477	8.36614970346347	3.59931344098345
H	-3.62748116150381	8.92005590716631	3.43016783425096
C	-1.67801213788895	6.40927392082221	7.33239508331157
H	-0.89143979374300	6.99057793304269	6.83925885554804
C	3.46311268432483	6.74947084012788	3.33897304964229
H	3.42081362414068	7.29304446346855	2.39382908763290
H	4.47887294844906	6.83643968952334	3.73770218411108
H	2.77250512143327	7.23290309289800	4.03560915183453
C	3.41735218886064	10.08015813833129	5.20237138998660
H	4.26919842051714	9.63159690285713	5.70208654421483
C	2.54347082505597	11.80105332381226	3.77127098782739
H	2.70530849107611	12.68547983646359	3.16104606516623
B	-0.84391543853875	8.34874313434290	4.44440551086294
C	3.47309954025838	4.48995418182542	4.40978000385559
H	2.93449263409998	4.89113662242730	5.27471814147799
H	4.55024837481508	4.56796113530064	4.59721576596325
H	3.22312362882137	3.42979802577701	4.30498733096007
C	0.08764241862536	12.06611897286202	3.31164214063618
H	-0.82264420709373	11.47858968323254	3.46280649502535
C	1.37802157498215	8.73217242722870	1.27123244133735
H	1.83406402143709	9.34462373127682	2.05523378983796
H	1.22968943477884	9.36171837876642	0.38508584736461
H	2.08533249619121	7.94545446037264	0.99102395563980
C	1.59398055679189	2.82064973567773	0.57058742455241
H	1.38258580630977	3.68416356966906	-0.05435946040514
C	3.62331492560873	11.18211917140110	4.37934984735267
H	4.62803913678959	11.56976785603551	4.23382517035831
C	1.92170045538389	8.43248893862402	6.41311066229932
H	1.26704062144920	7.67795666531402	5.95044312934922
C	-0.54369364072096	6.59423379055286	0.50429701910647
H	0.25246116962381	5.84620159278153	0.54445642440340
H	-0.51888600388586	7.06065355431676	-0.48955775813003
H	-1.50531486200148	6.08642656846963	0.61342205733261
C	-1.68326908502245	2.89051186501530	1.43808411424392
H	-2.68474410019575	2.45558821492385	1.35285920595370
H	-0.95635470420848	2.08190249379000	1.33296691502879
H	-1.54398035469111	3.59641661100102	0.61253593837380
C	-2.76295056709577	4.55238512844520	2.95381527810379
H	-2.78643458802202	5.29011736011757	2.14739974695317
H	-2.70473786199173	5.08598137187928	3.90607500627060
H	-3.70017360790871	3.98798544483089	2.93818725930694
C	1.66749201614354	1.79472423330148	2.75438947585321
H	1.51924907336157	1.85758136188839	3.82859871853671
C	3.89703888092658	4.74240028707175	1.94395294943033
H	3.84157447424086	3.65567626416081	1.85171213568197
H	4.94921855598758	5.01698894527406	2.07661014899127
H	3.54092279857048	5.19405265466919	1.01148412705006
C	-3.81697491605880	5.31722894602143	6.55900721569331
H	-3.66746329450919	4.56144364088990	7.32457415162352
C	-1.61264151339252	2.57492807837121	3.93326898574117
H	-2.56389700470434	2.03126252259419	3.91179956359885
H	-1.52379048030048	3.08168809541013	4.89991804922940
H	-0.80200509735781	1.84639503808515	3.84099570471957
C	-1.07586432387601	5.05412606004578	7.71138434267768
H	-1.76362493731242	4.45357241643289	8.31725018797547
H	-0.16962653387742	5.21322441373159	8.30703348765154
H	-0.79539401523291	4.48899216866965	6.81753416040828
C	-5.19896017709018	6.26904549062838	4.83791495377401
H	-6.12046560258529	6.25538729949952	4.26212424656260
C	-2.10764675252833	7.16685775124024	8.60113882523633
H	-2.48059055754604	8.16737453014161	8.36286840291299
H	-1.25823758289705	7.27304326999979	9.28535646375268
H	-2.90215832170628	6.61993394834849	9.12337054483178
C	0.26029690003105	12.29092014393609	1.80475322048930
H	-0.63193143634341	12.77822558831343	1.39560889223926
H	0.40525764513305	11.34551370278214	1.27725215481180
H	1.11863083436274	12.93755117416381	1.59207730857993
C	-5.58820529550387	9.34957987197515	4.17380680488692

H	-5.76749241743790	10.16942559683802	3.46888345772468
H	-5.24331508235149	9.77884138536576	5.11962581023685
H	-6.54299271744729	8.84342430099313	4.35966011313649
C	1.21343692600122	8.95518295839151	7.67676235509995
H	1.08462114828350	8.13528083090969	8.39283301660253
H	0.22765083363488	9.37117433984221	7.45991453183300
H	1.82310749456550	9.73212273070645	8.15394468717862
C	-5.03923041091112	7.81717464510726	2.25194257552019
H	-6.02035323994384	7.33794527270925	2.34088290765550
H	-4.33963629295876	7.07991932652574	1.84476241680500
H	-5.13772101726913	8.63422317133537	1.52838945331742
C	2.05208423516300	1.63912188514168	0.00064900868199
H	2.19894240087438	1.58071653511545	-1.07398768858401
C	-4.97637940529693	5.28944121040978	5.79441826570114
H	-5.71453654698469	4.50819713373058	5.95614903502134
C	3.20300994402826	7.71675575601769	6.85063633568872
H	3.83302800773998	8.36984353556525	7.46634950354167
H	3.80032726924735	7.35930857855120	6.00856817391357
H	2.93459187481317	6.84985158721709	7.46379840933433
C	2.13134747169867	0.61419786603365	2.18127713297592
H	2.34280820060499	-0.24394963225560	2.81279152268311
C	-0.10538256062773	13.41396569186303	4.02656630766237
H	0.76412217690331	14.06294491489665	3.87077093448186
H	-0.23588852782606	13.28297187733165	5.10551103975422
H	-0.98837855685874	13.92955271261722	3.63236614270671
C	2.32268270213621	0.53350475125447	0.80462059632460
H	2.68218837481533	-0.38925411929755	0.35827365123898

TS2

Si	-0.10369373837646	0.21375420450703	-1.31566773041825
N	0.75357225190032	-0.67266095971458	-2.67231168559257
C	2.16113139684798	-0.83342629688483	-3.08897299238258
C	2.84298472424912	0.53630725581696	-3.19725689341444
H	2.36197248716711	1.15133908456715	-3.96365641096947
H	2.78540948006141	1.06632584371963	-2.24170500081104
H	3.89813000963870	0.40865836000901	-3.46381888040573
C	2.83817210327928	-1.65409555119214	-1.98192694805218
H	2.67148795546080	-1.19403752970177	-1.00266222387152
H	3.91725285060859	-1.70802891231230	-2.15802464145280
H	2.43884222628201	-2.67216631173826	-1.95581158688193
C	2.28241271524534	-1.59563633397712	-4.41118779557968
H	1.94532508409675	-1.00270081034806	-5.26418577988096
H	3.33567820387812	-1.84937987323451	-4.56850376237861
H	1.70506466597436	-2.52637856598764	-4.38060951543407
C	-0.40424835255230	-0.68154507291348	-3.38019412758795
C	-0.53313403619925	-0.43055555625885	-4.83199354989252
C	-0.18901769690049	0.83643039811404	-5.31238794099369
H	0.13580325103073	1.60138244126011	-4.61104641136617
C	-0.29550891705326	1.11748491101560	-6.66864796229309
H	-0.03690480243318	2.10607387638375	-7.03739409865951
C	-0.73650705897757	0.13400895163875	-7.55294349295243
H	-0.81482329142752	0.35278971835717	-8.61401545618996
C	-1.07457945090295	-1.12985239764672	-7.07538559879458
H	-1.41213317093682	-1.89948411901438	-7.76349416823579
C	-0.97497585746707	-1.41413212820150	-5.71667183642489
H	-1.21735732242865	-2.40492548316708	-5.34269406139692
N	-1.37107716922539	-0.54831113853491	-2.43607972819974
C	-2.82181362835710	-0.31002916964950	-2.61403072143526
C	-3.36176799118838	0.04658986784192	-1.22612830084552
H	-3.10653938788003	-0.73251212364941	-0.50252144406727
H	-2.94525368080347	0.99745737323125	-0.87705162801079
H	-4.45127284741338	0.14181722513971	-1.26502948261707
C	-3.48180406131182	-1.61245613732729	-3.08506591116130
H	-3.28751782127397	-2.41970815677528	-2.37178323531937
H	-3.11115848568029	-1.91017156669894	-4.07014845330132
H	-4.56506843785094	-1.46786160583795	-3.15936745590366
C	-3.13886346111229	0.82893790150985	-3.59149955417875
H	-2.56990587444134	1.72615028089717	-3.33042698365304

H	-2.91422479390818	0.55768786459640	-4.62584077151875
H	-4.20692568818936	1.06421966750209	-3.52870084048299
N	0.13141485007790	0.59168729464514	0.32431419198138
B	0.31619909429868	-0.09273544704864	1.58597485437927
N	1.20190110069868	0.21795593863733	2.70356415872421
C	2.09710831947605	1.30096182614355	2.96929104364592
C	1.79961118928719	2.20280940006694	4.01229342110633
C	0.54150748313719	2.10312178066637	4.86194428255947
H	-0.13832580842189	1.39013268258913	4.38190729978846
C	0.87290444239599	1.57559048190750	6.26829954048201
H	1.38009804685312	0.60769903524648	6.23665296327105
H	-0.04454040912014	1.46239798422006	6.85717403608563
H	1.52790803718091	2.28027257761856	6.79407808393594
C	-0.19617569982605	3.44490788637031	4.99168937608597
H	-1.15952461600073	3.28732001961934	5.48945475926478
H	0.37280889857286	4.15719100127715	5.59890938145847
H	-0.38557358815777	3.90057867873372	4.01659516348536
C	2.71923091173693	3.21755700947025	4.29392373675699
H	2.50584213780437	3.92113926993402	5.09371231164620
C	3.89398396317399	3.34317330815110	3.57017817161286
H	4.59438079688003	4.14140508540220	3.80033507242053
C	4.17662117011792	2.44406751732480	2.54864932672729
H	5.10144101190545	2.55294507981531	1.99259173679829
C	3.29694304011387	1.40765021411437	2.23405644404420
C	3.64057611382816	0.39596410356144	1.15291022286433
H	2.72713707817851	0.24306757166281	0.56426590477948
C	4.04540415036582	-0.95380274081261	1.76979083442564
H	3.25102160705910	-1.37196557292158	2.39369747069767
H	4.94191812317270	-0.82956171183404	2.38875987117972
H	4.27686310949522	-1.67762336929826	0.97907845291510
C	4.73516709512339	0.86644094291016	0.19272629712400
H	5.70788928984668	0.93504280472815	0.69335987680949
H	4.84138133160204	0.14399997388289	-0.62271796918054
H	4.50526115566493	1.84189819574693	-0.24879568308156
C	1.05334259817137	-0.78557001347463	3.67534617954718
H	1.66173666243330	-0.79734406142875	4.56768950126505
C	0.13819659166906	-1.68796236095779	3.26738283792066
H	-0.20783448283430	-2.58087380314837	3.76949895486308
N	-0.34079050202950	-1.32382472538227	2.00142882148296
C	-1.53653671985747	-1.93205751697096	1.51446375210481
C	-2.76300996307554	-1.56168127432534	2.09720588969754
C	-2.86192526038447	-0.44835815078887	3.12342328135257
H	-1.88455734633590	0.04306628656747	3.18551646478962
C	-3.19864419097776	-1.00081296532955	4.51589973909648
H	-2.45555450846535	-1.73550949984938	4.84115469987000
H	-4.18162128737319	-1.48674253815391	4.51465383456044
H	-3.22335988566461	-0.18872229220352	5.25146631793659
C	-3.88672505693318	0.61425393475874	2.70226690824541
H	-3.68979760358682	0.97055643742712	1.68621246981169
H	-4.90913994178306	0.22127964089792	2.73253601779723
H	-3.83797194875097	1.47197182843619	3.38231267071750
C	-3.91875959463957	-2.23920227310314	1.70069755785137
H	-4.87432304320528	-1.97493754693194	2.14613442161160
C	-3.86403455907933	-3.23663765336870	0.73855429721315
H	-4.77088492139913	-3.75526810346426	0.43904504092067
C	-2.65088422989681	-3.56306159076701	0.14288229195130
H	-2.62908160469588	-4.33370268845622	-0.62057051275472
C	-1.46779139887768	-2.92149193366162	0.51532313917468
C	-0.13723520061507	-3.31438156859189	-0.10374646749039
H	0.47132567301934	-2.40255179427621	-0.19141237755050
C	0.62836045910870	-4.28197946159863	0.81376390903052
H	0.81188267530131	-3.84303310740853	1.79810397289851
H	1.59637043537952	-4.53967215759140	0.36822897778215
H	0.05551898173414	-5.20728281991317	0.94771327086889
C	-0.27631287837493	-3.92051325652669	-1.50170318818685
H	-0.84081413499711	-3.26341535986694	-2.17116466945219
H	0.71570269277858	-4.08039443112414	-1.93523920702629
H	-0.77067205064718	-4.89789300968586	-1.46903154464828

Si	-0.11723638141347	2.41242955403751	-0.29040432528207
C	-0.83678541660518	2.97757905449043	1.38519722032713
H	-0.23750819399607	2.61248206079632	2.22363914367245
H	-0.88281269903284	4.07210717677809	1.44264911878864
H	-1.85717700003359	2.59515811762900	1.51807010211544
C	-1.23755828928536	3.48463878083296	-1.43648852818366
H	-1.20198342927174	4.53239724455255	-1.10245939785154
H	-2.28658654802843	3.16796048658706	-1.40981494459908
H	-0.89555625396163	3.43126797053028	-2.47639965930441
C	1.58535972066296	3.21836459363424	-0.46152533225405
H	1.50227756167531	4.27573949417721	-0.17657362069536
H	1.95891243719038	3.17857850019036	-1.49051829017376
H	2.32693322903439	2.75639754815394	0.19677468840191

4

Si	6.06210340875857	24.97729422691806	2.71512180376315
Si	4.86351006755919	25.97093124908073	0.95006197899603
N	5.23414342793193	24.59715227885297	6.64449858770941
N	6.56265797920785	23.23716278881969	2.21475791875757
N	5.32457882380541	26.85087688623377	6.28181855396468
N	5.62015623377772	25.36800084727732	4.20212023617378
N	7.85771242975662	24.95170862567627	2.21831058357612
C	5.08093102286918	23.19023957701804	6.55192724304368
C	6.21782582240224	22.36477337517583	6.55670611023482
C	7.78661397657459	23.66087444317227	1.87323766375795
C	3.78350739368683	22.64596159039990	6.46135036600264
C	5.33349286852299	28.16592214834014	5.74811176238615
C	5.08799204587497	25.27046208046717	7.86422132930688
H	4.94503040095220	24.73865377241829	8.79555680199765
C	8.84407144677198	22.87702087313568	1.18668727992190
C	5.87318480551798	21.95705753497045	1.95690046469054
C	5.15298494264644	26.60510281465671	7.65033468289646
H	5.07096382847941	27.40764508087336	8.37028610915577
C	3.65025302755651	21.26054733287353	6.35914094846142
H	2.66079740430822	20.81861841268663	6.28060971188795
C	4.76865020446764	20.43333806523527	6.34776749500365
H	4.64611345418013	19.35652634270469	6.26459506344816
C	4.20500636072548	28.62252816956686	5.02818924264189
C	6.04000264421164	20.98228758467797	6.44722338868749
H	6.90787204251250	20.32680759454749	6.45097112084820
C	8.86813125710729	22.83439778389999	-0.20823710218134
H	8.11431712452486	23.37255492502601	-0.77668850190516
C	6.45181830809767	28.99575434950174	5.94802646256911
C	7.61040208983635	22.94199708603163	6.71470427067222
H	7.51447731491811	24.03306030426022	6.71625725191096
C	2.97775940498571	27.73756635956202	4.87244854735592
H	3.33658674021335	26.72919040288945	4.63072505377111
C	2.56504127379944	23.55069032133483	6.41902994133723
H	2.84490140674816	24.50374085680261	6.88204443005463
C	8.98956727954557	25.89254921513355	2.28568384947869
C	9.82438271488292	22.21183582128375	1.92407576478024
H	9.79983243278595	22.24104353120626	3.00954057810074
C	4.62170893827916	21.98093395969219	2.83836222959323
H	4.89092883515322	22.05521141470311	3.89595405451616
H	4.04318187097826	21.06441831043562	2.68726845880287
H	3.98332136242913	22.83521819145193	2.58695409606059
C	10.82364250311529	21.50435900539797	1.26448890431215
H	11.58457665606220	20.98633866568803	1.84079251845592
C	5.32846919481056	30.75064532246550	4.71051518627520
H	5.32477081005768	31.75883098683157	4.30467993542073
B	5.41864034379481	25.57036681791677	5.55967684531500
C	5.45771457073309	21.83762730244900	0.48520486165333
H	4.81395370503657	22.67487924552724	0.19893672436733
H	4.89593789926593	20.90907345107469	0.33649105999082
H	6.32850438713675	21.81754391692256	-0.17704546105477
C	9.86963804757426	22.12495453390873	-0.86169155112652
H	9.88795013068115	22.09623260797076	-1.94722810871166
C	4.23023990882621	29.91870719106798	4.51291332555371

H	3.38018718365729	30.29231702876765	3.95064484342759
C	2.17515405921701	23.84475842255495	4.96183435268324
H	1.89293520334605	22.91839680576090	4.44579684412563
H	3.01150843682028	24.29882681296376	4.42022571999569
H	1.32153358757239	24.53248415740506	4.92506797905390
C	6.74351240624317	20.75909031105525	2.35610346602748
H	7.59706677532971	20.63232862150345	1.68563382526272
H	6.13604358210314	19.84953482273220	2.30619481417403
H	7.10879148629577	20.87197579043383	3.38196348430725
C	10.84831142503396	21.45940587975083	-0.12730657369892
H	11.63062134555495	20.90700541751469	-0.63969714515036
C	6.42460686317930	30.29293039966523	5.42594595308507
H	7.27641966808165	30.95068670318469	5.57908503993709
C	8.37398560599682	27.29612979392724	2.28664652421907
H	9.15749078082095	28.04551707868487	2.43437287891193
H	7.63550374462141	27.40414878224090	3.08958773152719
H	7.88177141517054	27.49744032612027	1.33003884824935
C	5.61112084811311	25.58515427372118	-0.74540784133969
H	5.04217499405757	26.09260646083763	-1.53453438971955
H	5.61148643559333	24.51357701612451	-0.96936741000582
H	6.64645457724609	25.94221449831754	-0.79948558222608
C	7.66408682803486	28.52442501646205	6.73014626300144
H	7.55933415863959	27.44417046878402	6.88233382947792
C	8.51608977365183	22.55701119499096	5.54071764758097
H	9.49356605101980	23.04400244974726	5.63514181935834
H	8.06058132316565	22.87244318738950	4.59607566199103
H	8.68123610865136	21.47361116586462	5.49749265280962
C	9.94403615955948	25.77451926401861	1.09193261518549
H	9.39628278690076	25.84631571415897	0.14620519051506
H	10.50729890821153	24.83871694686843	1.09996431940723
H	10.66087613947684	26.60101442103476	1.13747789722820
C	1.37078301444916	22.99090701133433	7.19807899030455
H	0.57596219526247	23.74325146331977	7.24694674239329
H	1.65269318511433	22.72368002433697	8.22221784781342
H	0.94786842907627	22.10140191867788	6.71778381981880
C	2.19268643870161	27.65740659241304	6.19337852008842
H	2.80574029375143	27.25846324883918	7.00562441398479
H	1.31995602212785	27.00463241001388	6.07193811050923
H	1.83584646839615	28.65357773916384	6.48226841996704
C	8.22867200731909	22.51854811054386	8.05433785832227
H	8.36098747165085	21.43115726178973	8.10090866129577
H	7.58862518451517	22.81726624574859	8.89129048258660
H	9.21092548300932	22.98596093134006	8.18872904673243
C	9.74919327996330	25.65745593511802	3.59909204214204
H	10.55095970311382	26.39596456444155	3.70912402586029
H	10.19736889212441	24.65858615475780	3.61194666841065
H	9.07050513049178	25.74589281013570	4.45425707344146
C	3.07127299848512	25.37523516517874	1.02062738354633
H	2.64903424774652	25.54937194849508	2.01721255512491
H	2.98526857828393	24.30517392426384	0.80228986031969
H	2.45796275228491	25.92080585569073	0.29282436067936
C	8.97767857849508	28.76598448417580	5.97518417438697
H	8.94472457462222	28.32745337349795	4.97406024487362
H	9.81276420701461	28.31262650905563	6.52141752497047
H	9.19302529189455	29.83521370058252	5.87140112703539
C	4.88172249523973	27.84740668018000	1.18258546799383
H	4.03817995859818	28.30072778160909	0.64732037277469
H	5.80263664439328	28.28806825314796	0.78761301336820
H	4.80621816058165	28.12628205472376	2.23918332043451
C	7.71784235580877	29.19846894538210	8.11008961162071
H	7.84474797575521	30.28250723056546	8.00366215783213
H	8.56178247077370	28.81279302049521	8.69356014207421
H	6.79920181218721	29.02244949370856	8.67776926842001
C	2.02975406877840	28.17020903478176	3.75141140649773
H	1.53233360357351	29.11948689249208	3.98362160221283
H	1.24524809378670	27.41494504739214	3.63072831403237
H	2.54360669682431	28.27806337380171	2.79156403604568

Ge(IV)

Ge	9.92928809662217	7.17405189427138	4.77726045704699
Si	10.44927619706112	4.01098043759681	4.92864726731139
N	12.61344787725385	8.63286196024654	4.81281972963193
N	11.05416544825313	10.17342059150701	4.19402637739491
N	8.20232011705520	7.19119908062689	3.75387704512740
N	8.27975638545182	7.52579305836686	5.91034503448849
N	10.75389157273429	5.67685359536148	4.99011515212677
C	13.69419473115322	6.98997226742870	6.28439119934373
C	12.31674345919912	10.77494601209067	4.23508132571488
H	12.44607901103697	11.82561648559670	4.01911805206368
C	13.24087084455299	9.85897220621686	4.60362830270294
H	14.30817369600464	9.97709812520452	4.73068429510799
C	9.95642696679788	10.93572499972574	3.67610752636989
C	13.42581652946923	7.46303889371470	4.99108388061261
C	13.12018953939748	7.67561109264017	7.50861584744929
H	12.20526353550269	8.19283342966725	7.19565615350043
C	7.47536016051178	7.39603683995456	4.85066696634516
C	8.96949782846013	11.42759540395449	4.55518319938842
C	14.52291241221950	5.87496647367873	6.41316568529662
H	14.74395476397409	5.48040239528096	7.40022061728475
C	9.94201826756288	11.24310930856522	2.30179192006005
C	15.06408884160176	5.25161173771936	5.29489236701311
H	15.70175087536873	4.38003013443710	5.41584096154293
C	8.90151900306622	12.03947362893569	1.81495111717559
H	8.87097496789757	12.29017185675372	0.75837102671438
C	14.78686172305914	5.73814925914949	4.02524787212845
H	15.20780155328741	5.24058402156168	3.15529312665998
C	7.99576083501667	7.26319858374061	7.33841993491216
C	14.09551313720792	8.73910954509833	8.03812184787103
H	15.03946253224724	8.27162810487602	8.34264877728195
H	13.66940556520534	9.25172758657023	8.90834965630010
H	14.31887825229352	9.48985506322096	7.27350914050600
C	13.96529341284000	6.85329131885816	3.84871703944855
C	9.06227559404703	11.18712013729255	6.05346167102246
H	9.28792672452752	10.12302530083601	6.19852440305365
C	5.99210259644716	7.47888276375478	4.89286925644387
C	7.82375674999542	7.24418058538883	2.32932251596790
C	12.74262153330623	6.69225205407353	8.62088782639019
H	12.11874670082929	5.87870698590717	8.23501792646391
H	12.18441184626666	7.21492104428538	9.40531391057823
H	13.62888701563197	6.25144120138478	9.09025974407097
C	7.93700339877112	12.19482194051543	4.01213936825904
H	7.15459720589474	12.57994611514372	4.65703881845681
C	13.65992143558483	7.34461126698903	2.44560463275662
H	13.01304710325044	8.22452667747272	2.52619282429603
C	11.01486728724458	10.75970233005959	1.34021915804672
H	11.61440467016956	10.00015456304175	1.85427107828081
C	7.90192872926895	12.50070206919963	2.65601194276560
H	7.09325411297892	13.10845036658697	2.25933140890862
C	10.42688052344300	10.11605890404934	0.07628942648761
H	9.70100169480395	9.33711549028023	0.31911279963857
H	11.23070072156083	9.66379909521040	-0.51506609322201
H	9.92996918961224	10.85802705202346	-0.55806684720067
B	11.19885011197131	8.78356979384164	4.56508106508342
C	7.76974140291400	11.50314240235412	6.80687729423972
H	7.87342049536690	11.19377758239669	7.85195362438007
H	6.90302179869767	10.98610036830128	6.38405236263118
H	7.56082212399338	12.57928492652324	6.80593145104912
C	5.37092310454728	8.72780275205997	4.85912556540052
H	5.97767643930897	9.62503758631647	4.76995938938702
C	10.20664824080631	12.00493290649612	6.67939881556386
H	10.03642844544781	13.07580273787299	6.51669510827846
H	11.18057726018828	11.74246612029346	6.26183110702819
H	10.24291324080045	11.82633781282945	7.76015314191642
C	5.22165457961468	6.31924240313442	4.99198492187753
H	5.70685178792927	5.34697101496273	4.99541941917874
C	9.10201011973138	6.88028362689231	1.56555475715387

H	9.91207465400875	7.58188794431823	1.79982129785764
H	8.92176770701000	6.91232565904158	0.48669869219225
H	9.43692631098707	5.87271858465893	1.83561266771351
C	7.35745903721639	8.65262743870546	1.94167337168689
H	6.39783875622177	8.88977968439935	2.41003722500402
H	7.22773371266464	8.72057254595734	0.85554383645417
H	8.09076452846896	9.40225501814497	2.25119771843919
C	7.83141322256365	5.75312676954773	7.56414763576474
H	6.94389852691471	5.37580822824816	7.04656938552737
H	7.71853004129675	5.54173259382360	8.63338724903762
H	8.70880073068650	5.21917164879394	7.18948122133058
C	10.93689065180148	3.19961913553715	6.57057400302870
H	11.98455447696251	3.43187281040963	6.79978382614443
H	10.83552454694468	2.10814738609452	6.52452430750661
H	10.32810906711476	3.55924376615031	7.40851772172597
C	9.23239000628837	7.74464535833757	8.10477563304107
H	10.12691178133014	7.22917616091050	7.74067455846857
H	9.12270737886256	7.52124010361161	9.17052495148128
H	9.37659550559150	8.82465991106260	7.99031534896561
C	11.53870103192105	3.20456030234511	3.60559952326424
H	11.25428017480632	3.53868246080994	2.60040246234689
H	11.46301525812119	2.11021770669871	3.63216889130990
H	12.58927074305481	3.47995492553422	3.76086362993839
C	6.75775210458830	8.00070084900514	7.86298584690651
H	6.80398766399258	9.06656997647104	7.62790984798820
H	6.72283091010401	7.88947809627772	8.95220633448584
H	5.83004999920685	7.59353237835268	7.45470551642148
C	6.73368537172646	6.22037222011547	1.98061977940262
H	7.01113311089066	5.22032468505227	2.32735974366757
H	6.61165791297960	6.18830351735468	0.89255071607652
H	5.76988766264067	6.48880602463553	2.41935634417802
C	3.83663213971430	6.41343417518852	5.07607170498130
H	3.24030950693310	5.50932099842260	5.15740791458584
C	11.94588446998419	11.91481948148216	0.93528951437632
H	11.38542515041855	12.67827450404945	0.38312815187414
H	12.74688715906461	11.54487231107210	0.28548834345894
H	12.40426889641430	12.39616870852234	1.80327151171012
C	3.98544181534629	8.81668462209179	4.93577382257625
H	3.50572468450260	9.79076947335072	4.90751667552494
C	14.93385361495351	7.77626051552179	1.70762697349568
H	15.61340449306707	6.92889412840051	1.56269430402652
H	15.47187248595411	8.55006158971220	2.26594880563004
H	14.68171923307895	8.17729781673708	0.71918314179979
C	12.89009297911879	6.28251277020499	1.64881337181183
H	12.59317894020063	6.68103739432550	0.67125931103449
H	11.99162345603890	5.97337820972021	2.19123302397718
H	13.50420619877182	5.39048371608063	1.47985661349793
C	8.67266023586488	3.47340858875748	4.52453941446638
H	7.94173196754942	3.90290292558864	5.21886907973951
H	8.57599464070606	2.38095407296553	4.56646032211225
H	8.40010265560816	3.79120414627119	3.51101821437459
C	3.21676930530153	7.66076566149824	5.05161508545795
H	2.13489126011911	7.73142692924033	5.11758271557428

Ge(II)

Ge	0.20894838776337	5.81654157653616	4.22362010411963
Si	-0.22521168914952	8.01252979249037	1.81087661291041
N	-2.11248141992584	8.35512978790016	5.22006875310296
N	1.67529032658328	5.12428253929792	2.93492812949244
N	-0.36350328209334	4.35056808534386	2.90953015608044
N	-0.28735943090547	9.69362587047854	4.90675683068504
N	-0.34836871897348	7.48247215925181	3.47932155394837
C	3.13914928636482	5.11726097707981	3.15176619532084
C	0.89568537039619	4.08924291004017	2.57746904378531
C	-2.26789353243107	9.53700850474759	5.95542045523880
H	-3.14254315803288	9.71289939682663	6.56545484274435
C	1.37343021086546	2.83851934310908	1.92434900358787
C	-3.06549376517072	7.30295204192340	5.38754318187835

C	1.01807434349626	10.24530919166748	4.72257812089994
C	1.17746515838680	11.41174185814817	3.95283325761488
C	2.12169587523510	9.64442587224732	5.37532148073100
C	-1.19537145894142	10.33291724958044	5.76373646591521
H	-0.98458615764602	11.30160749976979	6.19446643284537
C	-1.60003210123104	9.24993596086707	1.43794853190413
H	-2.56342246703755	8.73387358091837	1.37916156360249
H	-1.41650813127453	9.72166449651108	0.46484388032495
H	-1.68924959347616	10.04630359737158	2.18355705262072
C	-4.23045759371207	7.28705675527230	4.59902653797109
C	-1.57421498964492	3.52024465774653	2.78969138829499
C	-2.85764700453082	6.34458112748180	6.40336317701121
C	-4.51613654444297	8.37308017791552	3.58194038503500
H	-3.58588868037107	8.92632965588974	3.42170755358794
C	-1.69357689049498	6.44580304001105	7.37311469751287
H	-0.90050371480720	7.02538827704534	6.88707416970719
C	3.55279184642837	6.58684813447291	3.28667998989879
H	3.52200495824552	7.08817817824452	2.31692163955264
H	4.57299030711279	6.65875215669544	3.67754179526995
H	2.88093505848947	7.12475037145011	3.96280266399245
C	3.38881947541377	10.18435606085704	5.15017264379507
H	4.25659487513688	9.73396065390981	5.61977831917297
C	2.46588566143349	11.92494263948173	3.77528168588616
H	2.60560448355542	12.82019515019879	3.17551945271509
B	-0.85889306594764	8.41936338915787	4.47106991215206
C	3.47762236001924	4.37251828707915	4.45359567794427
H	2.95959280910700	4.83137885451771	5.30264052098083
H	4.55670326564608	4.40887165281063	4.64257454249072
H	3.17800368994594	3.32152631420952	4.39076547450501
C	0.00161701643400	12.15608292159782	3.34699564652115
H	-0.89726478359658	11.55122380242273	3.49985437315011
C	1.43168283343040	8.80244429784553	1.36580180591599
H	1.86144208489839	9.40262219013463	2.17382830100000
H	1.30394217108110	9.44929238076443	0.48904218438479
H	2.15560594460509	8.02928109771735	1.09144134872756
C	1.54837826088799	2.80734990294946	0.53929615790645
H	1.33042299552495	3.69428975126631	-0.04951375864562
C	3.56514325313185	11.30654863868924	4.34742910391344
H	4.56240555967302	11.70743270504198	4.18763987036624
C	1.93875212804466	8.49829158458633	6.36134170444718
H	1.29508565552259	7.74201777624304	5.88867501981642
C	-0.44678395299148	6.62081582133934	0.56472030711641
H	0.35425747056177	5.88067047940530	0.63984427759795
H	-0.40293005828484	7.06693544577487	-0.43766830685260
H	-1.40677424684481	6.10662599497922	0.66363305834842
C	-1.69259887846222	2.82259913752561	1.42763482670721
H	-2.69981040795737	2.40224127883536	1.33594014175673
H	-0.97592803977057	2.00622968167345	1.31367201566369
H	-1.54246159526139	3.53675199513494	0.61093722813561
C	-2.75608589632526	4.47962733537329	2.95726658621046
H	-2.76349203154817	5.22833381579446	2.16012558019486
H	-2.70066179589935	5.00180324999550	3.91717486250006
H	-3.70042998448065	3.92746382092709	2.92751525978720
C	1.65034912835804	1.69959801693319	2.68136274742677
H	1.51898533136008	1.72255248764548	3.75934512597783
C	3.93519176480524	4.51023592354540	1.98709253297330
H	3.84920908590833	3.42273465725502	1.93936019411045
H	4.99315021809655	4.76000051131551	2.12413224915351
H	3.60571622163882	4.93102490239469	1.03115549324683
C	-3.81410657243919	5.33960297027634	6.56731965006920
H	-3.67220875717195	4.58497643829625	7.33543747086085
C	-1.62365896940216	2.48203895368578	3.92144251418328
H	-2.57520734902551	1.93923142660967	3.88996986903736
H	-1.54194592146276	2.97952597637138	4.89427080050608
H	-0.81233403585483	1.75414340027241	3.82854701665369
C	-1.10334482516744	5.08954813408316	7.76960476661599
H	-1.79203667871886	4.51302149921717	8.39717031323617
H	-0.18674019457968	5.24614683765677	8.34943378170745

H	-0.84784385618321	4.49560180150468	6.88697408855252
C	-5.16661952876633	6.27287165357313	4.81275335884873
H	-6.07346621275531	6.24609478282983	4.21465979852497
C	-2.13011983465995	7.21282799833815	8.63357162534195
H	-2.49183203020981	8.21502813156671	8.38687043655312
H	-1.28646497119172	7.31521140766316	9.32558507075663
H	-2.93407118729779	6.67433964610781	9.14991282521791
C	0.16557702222577	12.38702150893090	1.83964340950134
H	-0.74392054603978	12.84038415881419	1.42952287398348
H	0.34700880923969	11.44706099989213	1.31295455809962
H	0.99938406888835	13.06513322120994	1.62724265111965
C	-5.55649491845804	9.35970273470424	4.13674016326179
H	-5.72792745529494	10.17443128179271	3.42398736258922
H	-5.22306505994465	9.79574021890347	5.08356230700487
H	-6.51286590645726	8.85345642654918	4.31427937950334
C	1.23137196102000	8.97610756354736	7.64343299886349
H	1.12527809889365	8.13694161526165	8.34080100713293
H	0.23632422086556	9.37939132840132	7.44591023965810
H	1.83080372725750	9.75271716304917	8.13374551927701
C	-4.98058296306159	7.81527596309647	2.23165110597560
H	-5.96419881446432	7.33896628367759	2.30816047726676
H	-4.27575356986448	7.07419487303798	1.84053839473420
H	-5.06600214749358	8.62744276435238	1.50110565686146
C	1.99483844103687	1.64707572545074	-0.08136649569736
H	2.12622833251284	1.62876493618864	-1.15947373506479
C	-4.95662510029839	5.29939530691946	5.77849976281189
H	-5.69025005770177	4.51109248825967	5.92573224787975
C	3.23898172403747	7.79992914203511	6.77027700480354
H	3.86185210200442	8.45427221536441	7.39176773464682
H	3.83508724906715	7.47151162901836	5.91556330638631
H	2.99535212619487	6.91614965710472	7.36963901249447
C	2.10293186442165	0.54032394477410	2.05771734990274
H	2.32138719930147	-0.34150185429811	2.65318955841496
C	-0.21228949698254	13.49755939472396	4.06709905524305
H	0.64681722849558	14.16097348244428	3.91429622856512
H	-0.34074988909886	13.35986119634384	5.14546334307163
H	-1.10330846271748	14.00110096107413	3.67532584880082
C	2.27482832732600	0.51144708648833	0.67637470201257
H	2.62641299805057	-0.39419390682741	0.19042376984043

Ge(IV)′

Ge	5.84499223432865	25.00082677121429	2.59821264072392
Si	4.67448322972483	25.88151279916403	0.70668399860187
N	5.40335086304395	24.61536405356978	6.55052943390140
N	6.57412537482058	23.14582059962716	2.23676604136713
N	5.40767629952618	26.89016447624100	6.30542898734085
N	5.30357920308386	25.58803184839090	4.11516658555028
N	7.78727817547888	24.95316207650201	2.11927222764362
C	5.13706852081311	23.22724075782084	6.42830229090397
C	6.19132546771128	22.30316171402218	6.53882607366221
C	7.76819319584452	23.63885871614444	1.89313966815806
C	3.80862096181261	22.79714317881632	6.23835638945532
C	5.34245390395441	28.23255594707861	5.84931780981526
C	5.41531366099656	25.22429073721638	7.81443085716004
H	5.40445706773592	24.64108473917364	8.72576329747030
C	8.88922998869901	22.86898661662321	1.29004508028632
C	5.95210594178276	21.84408826937457	1.92158676356660
C	5.43396645086561	26.56865312079448	7.66723808616865
H	5.44421675042420	27.32926607319369	8.43577551862702
C	3.56514269804224	21.42762529812847	6.11386829550441
H	2.54831400994765	21.07609704024888	5.95648121769552
C	4.60285349344155	20.50606076920380	6.18663870838255
H	4.39570456152874	19.44420388606412	6.08187064008325
C	4.14419212185482	28.70265224497323	5.26251126733146
C	5.90421738447729	20.94237309672294	6.40322728728906
H	6.70820041081296	20.21354686529003	6.47495987807136
C	9.03705155300709	22.85312932704025	-0.09840750060239
H	8.32760186285780	23.39237252952174	-0.72063008628010

C	6.45856081185828	29.07713651976476	5.99490177325050
C	7.61203230807455	22.76117036017079	6.80726122701031
H	7.58115711512585	23.84222873669156	6.97716008136712
C	2.91764458167700	27.80736711626212	5.18422668386607
H	3.26108032841066	26.81627821336684	4.86462422408301
C	2.65602259072207	23.78232417446895	6.17460459799426
H	3.04806467144494	24.77035564033629	6.43938586757189
C	8.89996157428938	25.90647852368670	2.26247425819529
C	9.81097223775911	22.19852261787591	2.09466750217179
H	9.69386528526977	22.20746227292460	3.17395407416707
C	4.59843906685340	21.85983650258891	2.63697588023678
H	4.73046400667476	21.99723126811804	3.71399535472061
H	4.07215459026573	20.91489841691750	2.46923792631696
H	3.96545529230501	22.67250983763798	2.26084215236284
C	10.87517632850534	21.51832346297816	1.51143654677717
H	11.59195871354605	21.00066838491395	2.14223782296639
C	5.19614781781223	30.87043301450204	4.96334705328075
H	5.13709989226065	31.89887453683286	4.61690019563965
B	5.40128372659556	25.64987221310904	5.50540794133052
C	5.72884001524558	21.67507093796432	0.41331676366862
H	5.11243451996165	22.49136341189749	0.02257545612914
H	5.20691871564353	20.73165719154801	0.21901655436623
H	6.67780542718479	21.65686101506425	-0.13000780146733
C	10.09754037266739	22.16521089394981	-0.67701332305401
H	10.20732515413777	22.15525621899982	-1.75751974474736
C	4.10078903558384	30.02397614125618	4.81741336559223
H	3.19570405604669	30.40854699296282	4.35799420793411
C	2.08628300673926	23.87644349477574	4.75254114252401
H	1.68537882773079	22.90920141837409	4.42460155574665
H	2.86645793754972	24.18940614749355	4.05123076519516
H	1.27377495902401	24.61230245188299	4.71636609625892
C	6.78546551070437	20.67711044324995	2.46862888811675
H	7.73047803175208	20.56436830933767	1.93190440108658
H	6.21743192595133	19.74755132127519	2.35558177017892
H	6.99447690731558	20.82558255477253	3.53280421290210
C	11.01996190211278	21.49892355358096	0.12664646449712
H	11.85103071353572	20.96609287344079	-0.32630962712468
C	6.35945314029461	30.40049128019248	5.55335557679596
H	7.21020351153834	31.06768075744697	5.66665980659184
C	8.27004659611753	27.30057639615650	2.15716307431830
H	9.03080789863123	28.06913922821806	2.32363207041161
H	7.47600885358424	27.43274657737765	2.90228987834721
H	7.84306478077301	27.45116137964310	1.15946877463049
C	5.63597188448607	25.52944137729551	-0.87494316688036
H	5.11812530308552	25.96643465983797	-1.73756047768364
H	5.74631927895187	24.45550502419481	-1.05698084539842
H	6.63825750961079	25.96933529250728	-0.82316559564410
C	7.75173712549244	28.59438875194661	6.62631295553502
H	7.68335680474592	27.50579634590976	6.73284808188644
C	8.50731646286834	22.51057969940327	5.58911985799781
H	9.51636220454976	22.90236882276523	5.76550202182484
H	8.09121988603499	23.00498983276810	4.70493481825404
H	8.59015708370028	21.43773045322632	5.37531146264130
C	9.96827382239184	25.76838664365189	1.17014438813602
H	9.51420310685165	25.80188176572153	0.17409424273682
H	10.53803389572627	24.84127467737644	1.26337108838917
H	10.66809324679233	26.60629541051371	1.25752469068829
C	1.55666464149782	23.43816220452813	7.18787386149344
H	0.78816956159557	24.21928898773413	7.18501339887535
H	1.96561686444444	23.36307429472768	8.20115733154818
H	1.06618523200078	22.48810472702500	6.94726682501713
C	2.26392099432083	27.66476279844862	6.56986571355002
H	2.96189143049583	27.25381102712394	7.30471432674547
H	1.39749203248665	26.99474635061165	6.51044595531393
H	1.91622244199620	28.64125960628857	6.92885655583466
C	8.19408326600605	22.10316609030757	8.06454706973166
H	8.29556490790928	21.01906256421579	7.93994383599787
H	7.55498845534950	22.28480542412126	8.93512860367945

H	9.18903926307654	22.50988097454070	8.27798374526133
C	9.53491876956136	25.72304157815802	3.64866118883813
H	10.31644975725274	26.47332072903385	3.81156532726776
H	9.98703072745389	24.72908539270255	3.73317367138249
H	8.77840797897472	25.82956630958347	4.43428475740758
C	2.94788277001366	25.13469049038066	0.63717049181706
H	2.42470867672406	25.28350422341515	1.58874476314158
H	2.97560921055652	24.05973655027573	0.42922824422875
H	2.36091285958913	25.61873475371515	-0.15305004692379
C	8.97384544534503	28.90335255008013	5.75170401313649
H	8.83870434562177	28.52008671250683	4.73678578848566
H	9.86920091651902	28.43695412731110	6.17852729056928
H	9.16047963834279	29.98103235534716	5.68638796570213
C	4.55594941795585	27.73673118077953	0.99834751403930
H	3.86374829879185	28.19256704315173	0.27965501039845
H	5.53075782808232	28.22274681584464	0.89017307516382
H	4.19261793374148	27.94597760809328	2.00994394646911
C	7.93990563287305	29.20170755633793	8.02521029570072
H	8.03765751261085	30.29199234405990	7.96039172900696
H	8.84730400652555	28.80701327349140	8.49676241012040
H	7.08960633336791	28.98002720097645	8.67711927120381
C	1.86756210212691	28.27379641428295	4.17308165665469
H	1.38078059616786	29.20354888852819	4.49054169419798
H	1.08581309702357	27.51153709879069	4.08359553996607
H	2.29634131241460	28.43514864018229	3.17861070640659

Sn(IV)

Sn	10.03259505414698	7.13857976181989	4.74923815965499
Si	10.37004105577883	3.83983272211511	4.78966210598020
N	12.78725194458978	8.77494906759693	4.82341763184514
N	11.21972568064506	10.28746851645663	4.16256574101901
N	8.06379245229197	7.15562842039476	3.76484601527932
N	8.18902547352654	7.48245871849242	5.95454848988981
N	10.89614302389520	5.43997646510374	5.03168263806732
C	13.78264923990763	7.13519902019818	6.35531868452987
C	12.47303306189490	10.90717621142977	4.21780328987280
H	12.59296325690928	11.95548863253749	3.98396538677096
C	13.40452744657835	10.00645197219579	4.61202996697875
H	14.46774067312001	10.14002562096087	4.75698996457087
C	10.10186817734317	11.00475826896603	3.62561070375237
C	13.57359698266287	7.59558756703986	5.04577806814220
C	13.18617824448618	7.85320977039507	7.55083133468592
H	12.28997408693328	8.38364179342039	7.20486482574966
C	7.38574816943096	7.33034313765146	4.89952677332378
C	9.09256131060936	11.46964578864398	4.49219904546273
C	14.57062579884454	5.99655460495529	6.52664740019632
H	14.74911967860691	5.61183343136772	7.52589016328203
C	10.07716889685756	11.27385977941662	2.24528386723015
C	15.11769536712024	5.33323124140478	5.43510831779790
H	15.71951346407115	4.44188068763331	5.58990564414440
C	9.01269546555113	12.02638382005397	1.74097171485523
H	8.97392936161983	12.25181470293331	0.67889384978413
C	14.88650199409110	5.79879373003102	4.14861765113899
H	15.30540071602511	5.26381735605254	3.30037563839336
C	7.91868581212229	7.24315773974738	7.38732061725506
C	14.16635595776254	8.90667160353597	8.09119580902294
H	15.09130262139025	8.42551407433042	8.43050230404674
H	13.72534767206251	9.44162898819455	8.94015012286675
H	14.42682776447221	9.63990906373927	7.32139963971741
C	14.11283026036521	6.93990934559995	3.92828012173515
C	9.18490783148232	11.24255613577777	5.99130134146250
H	9.51062371444523	10.20594038375924	6.14511452751501
C	5.89851336651331	7.33608977248135	4.97920289157991
C	7.62527308127501	7.27331842822184	2.36256177715598
C	12.75651577253139	6.89508803574389	8.66668302713725
H	12.13665686191881	6.08166022388214	8.27377822427421
H	12.17796708473318	7.44020910048260	9.42041829752287
H	13.61990456486380	6.45296952895430	9.17573862438787

C	8.03795170636300	12.19390508485690	3.93418915356239
H	7.23921848055331	12.56063209302012	4.57008447811952
C	13.84634212347131	7.40378716290165	2.50813962012382
H	13.25945445053513	8.32732974814537	2.55343438861712
C	11.16101139741793	10.78926982994384	1.29790963785632
H	11.79654836875538	10.08119831586808	1.84084030478429
C	7.99945042215684	12.47554834987814	2.57273218578782
H	7.17340426091260	13.05017077237703	2.16275920503056
C	10.58473897556251	10.05399988591749	0.07994763833287
H	9.91388083372516	9.24436572773629	0.37905992375266
H	11.39963988063478	9.62358823085416	-0.51236829567789
H	10.02615426020334	10.73382023693955	-0.57256147264960
B	11.38351971071468	8.91071814115607	4.54721236572907
C	7.85572561688064	11.42735525334951	6.72223712120817
H	7.96441225809089	11.11487926983559	7.76590514306065
H	7.05506037639830	10.83329490556917	6.27141207734487
H	7.54258768712578	12.47773355978220	6.72823539258303
C	5.19835014920397	8.54199780795119	4.95085193087220
H	5.74229293490151	9.47840420081701	4.86197651752115
C	10.24305431351530	12.16486495506018	6.62230202392704
H	9.96752268960662	13.21494159690944	6.46873068928406
H	11.23515578082419	12.00243689809551	6.19478646240788
H	10.30444986864385	11.98197337847032	7.70113128530562
C	5.20457504258960	6.13023923651134	5.09275137520826
H	5.75232880382243	5.19172596288974	5.09128309308976
C	8.81234267242428	6.76732032663012	1.53180621621576
H	9.71466772985972	7.35660796022894	1.74005389635651
H	8.59445961027461	6.85225053764265	0.46273084894042
H	9.02753388969611	5.71839440861296	1.76464400117755
C	7.34332571971170	8.74183043884465	2.02117479185300
H	6.47088291869068	9.10858422896059	2.57072080831944
H	7.14201354479645	8.85313589656733	0.94938670792198
H	8.20203532892566	9.36936280827509	2.27826610820515
C	7.85186136340699	5.73263238668763	7.65899063200751
H	6.98331476361951	5.28617731262167	7.16459760380434
H	7.76405079472933	5.54633899611799	8.73537610778889
H	8.75732362932127	5.23997827337436	7.28891733439418
C	10.83737381730409	2.79967225553566	6.30241163005879
H	11.90960903351830	2.89773322546116	6.51188859011726
H	10.61603513068740	1.73580650696070	6.15100689555871
H	10.29413619174785	3.13536329330495	7.19436252528046
C	9.12699493192631	7.82473390218178	8.13244014458701
H	10.05443861077081	7.35472384909218	7.78545335242471
H	9.03740683014739	7.63492142091400	9.20660554237638
H	9.20254747077076	8.90579343141088	7.97427570794412
C	11.27315844441335	3.08527379770230	3.30248968723363
H	10.97027759273974	3.58148555391057	2.37168930755786
H	11.07179923131944	2.01187281561675	3.19609168122521
H	12.35624348201060	3.22280065999106	3.41049318425683
C	6.64486772737231	7.92208764724914	7.90995797823451
H	6.62355071185068	8.98134334969961	7.63949045980815
H	6.63333610218668	7.84591049056963	9.00267937701319
H	5.73716911179841	7.44752404287857	7.53133776413719
C	6.39812684227747	6.41148555218546	2.02896153023584
H	6.54108064642637	5.37941507647202	2.36504201774684
H	6.25751272371302	6.40420885030090	0.94274894348570
H	5.48628405309242	6.80366316685858	2.48419909784188
C	3.81759335929154	6.13509065349372	5.19086753310393
H	3.28133623466361	5.19491725254897	5.28208004797096
C	12.04268215125706	11.95894364540584	0.83261957288432
H	11.45153034581090	12.67870218652216	0.25440203224064
H	12.85259482453376	11.59202351159699	0.19234016800834
H	12.48819277784156	12.49110341819384	1.67808846711472
C	3.81024880692867	8.54194596043954	5.04121432486814
H	3.26860641551808	9.48322703831726	5.01735559278147
C	15.14775366876786	7.72728762075239	1.76286959930099
H	15.77158788503067	6.83438147317785	1.64558857586726
H	15.73299267462159	8.48128134822951	2.30025827455889

H	14.92402710479040	8.11334550696069	0.76189513498647
C	13.01310409523425	6.36546695140695	1.74318103916195
H	12.74038342431481	6.75020021171226	0.75335482640774
H	12.09775902297982	6.11891618633965	2.29213785184965
H	13.57432261134818	5.43444297916780	1.60331850603482
C	8.51840150892284	3.54729883580769	4.47423225272010
H	7.91029880916309	3.83677019967884	5.33902030862450
H	8.32373947652133	2.48682135819085	4.26791927495921
H	8.17062115717067	4.12709706446791	3.61089348070525
C	3.11804140427831	7.33981860373655	5.16624440532761
H	2.03443275339275	7.34125169243681	5.24146561315306

Sn(II)

Sn	0.24128137737090	5.70331338347254	4.46425717506940
Si	0.09372427709620	8.09565649449043	1.94278969649131
N	-1.98565154066000	8.34553422719015	5.37680173327580
N	1.75326294060155	4.73256133027525	3.05798629900093
N	-0.38338642458360	4.17433409649445	2.93331094619791
N	-0.30679714769692	9.84471930302775	4.96649213066264
N	-0.23395902251344	7.59316141739398	3.58351029822142
C	3.21189119402881	4.58749256440297	3.20951697313966
C	0.86567785124281	3.82267690872274	2.64264155475361
C	-2.19735644489244	9.50388689630706	6.13462447441458
H	-3.05080730698975	9.60378396905773	6.78958718996965
C	1.23103210030302	2.57810102454512	1.90483070622157
C	-2.95203252466597	7.29588227101067	5.46244489990977
C	0.89638681108535	10.57377049916492	4.71935615526804
C	0.83083806764773	11.79150492312461	4.01639168727823
C	2.11575612976429	10.10965576227856	5.26187645301934
C	-1.21267937341654	10.38987780647996	5.88925576672851
H	-1.05098360364690	11.36276244336228	6.33065641306343
C	-1.31942735463974	9.17182484937592	1.30494766117397
H	-2.21440071974789	8.56061197821229	1.14539293352908
H	-1.04878048608690	9.62701674109647	0.34427297649483
H	-1.58111527598226	9.97978719752163	1.99599037309820
C	-4.05450147897303	7.30272302853430	4.58436670129514
C	-1.64599254694795	3.45040370994028	2.71762175728535
C	-2.84431498562606	6.33492331910574	6.49073722796918
C	-4.22956070417109	8.39357637290845	3.54630149006772
H	-3.24770239447630	8.84745505896028	3.37724075424615
C	-1.74988981338763	6.40059369431917	7.54205794275321
H	-0.91035061059954	6.96275349454331	7.11625312719391
C	3.73904036846755	6.01373227640574	3.39944556306130
H	3.61536222888999	6.59118868237619	2.47818342958064
H	4.80095868808964	6.00216799283682	3.66580876496522
H	3.19122675929681	6.52271208660727	4.19994114478939
C	3.26317013864454	10.87542924785446	5.05176681005018
H	4.21511595559287	10.54097944436522	5.44923295378558
C	2.00544337896706	12.53073120176256	3.84872489856048
H	1.97200111477446	13.47237535382100	3.30714723884023
B	-0.77793315620532	8.51517261262798	4.55754557680972
C	3.53535390681919	3.75879872049946	4.46340221855389
H	3.08198462456600	4.21450876593211	5.35126356528156
H	4.61924590064083	3.70517392378571	4.61781639558323
H	3.15358669654672	2.73741257500163	4.36567027229063
C	-0.46132779258895	12.32778267831082	3.42637570246294
H	-1.23333345887713	11.56094086540848	3.55116656085459
C	1.70635352335856	9.05770080351194	1.75136153576401
H	1.66199086080627	10.04630768491483	2.21679675375145
H	1.93284094236574	9.19095973999107	0.68556241301794
H	2.54404616057362	8.52047936298763	2.20866043069809
C	1.39358096110516	2.62935652790118	0.51879827249897
H	1.24916764236314	3.57294326635266	-0.00121290425449
C	3.21264387278622	12.07716377268165	4.35386605876474
H	4.11875929217001	12.65923923113502	4.20953446442381
C	2.17911171326271	8.83217400347675	6.08491725246451
H	1.62537813482336	8.06358042680436	5.52711109056403
C	0.21338831649880	6.64259542743048	0.75343226946132

H	1.10097643322697	6.03253830956553	0.94654758889377
H	0.28886576574350	7.04617399210962	-0.26430508059746
H	-0.66431144240262	5.99057982109581	0.79890762690549
C	-1.76969771864718	2.79685031766555	1.33275335020313
H	-2.81080149252173	2.49174202673036	1.18166428724703
H	-1.14051025038116	1.91042367982460	1.23169995835824
H	-1.50207108624788	3.50748019838882	0.54342464070609
C	-2.74105222786276	4.51250335058094	2.84862567236616
H	-2.62882005492694	5.27673270349606	2.07243016096903
H	-2.68961489073488	5.00696552629485	3.82435803493139
H	-3.73356734159360	4.06045722480504	2.75574852960162
C	1.41049192004604	1.36992001863991	2.57773695254961
H	1.28524864676359	1.32772336707307	3.65619082880232
C	3.92740165624782	3.98153415243324	1.99139168216721
H	3.75262377347625	2.90841525861335	1.89330883519848
H	5.00535877680785	4.13964181346314	2.10680943464104
H	3.60531367114986	4.47394405398168	1.06797624481282
C	-3.83581149707464	5.35385573255303	6.58516151674317
H	-3.77056654771684	4.59993803186230	7.36411306511600
C	-1.84633036881154	2.39385385327947	3.81568282569944
H	-2.83170871324708	1.92408109033182	3.71597435773950
H	-1.78541201710729	2.86054085595228	4.80585978648889
H	-1.08524232769552	1.61044960853797	3.74921411878659
C	-1.23214180358805	5.02343071794014	7.97126636718364
H	-1.97385547519532	4.47963599570329	8.56660701295585
H	-0.33967906401951	5.14588205576941	8.59466439213593
H	-0.96109008407180	4.40734325903992	7.10823975003025
C	-5.02956759295666	6.31509041940595	4.73302900330781
H	-5.88863025629097	6.30500185277737	4.06834361967467
C	-2.24613359309496	7.16731843949380	8.78086563847560
H	-2.56037238046339	8.18313843122298	8.52864677822908
H	-1.44743181407553	7.23312429388665	9.52872648344261
H	-3.09783864171136	6.64734268399701	9.23565811006197
C	-0.31999160269276	12.62215321881011	1.92523382983499
H	-1.29721982747061	12.87373628355900	1.49777597607448
H	0.07789076910363	11.75894001098699	1.38406541109904
H	0.34862089709616	13.47170470743377	1.74841065289123
C	-5.16786543972426	9.48741506089875	4.08267974188994
H	-5.26635929981041	10.29812832772765	3.35159806134684
H	-4.79078627680706	9.91270670297978	5.01736467658452
H	-6.16570654168532	9.07364410440610	4.27243899411535
C	1.49517701773098	8.99824436118064	7.45325543553434
H	1.55468012685419	8.05736569413585	8.01342690135154
H	0.44183545096307	9.27372739597338	7.36099715217706
H	2.00530029609105	9.77445055339473	8.03632915405769
C	-4.74800623267381	7.87266420903298	2.20203571316085
H	-5.78192508012697	7.51764300919301	2.27535409395055
H	-4.13183811012053	7.05064876309985	1.82484875723245
H	-4.73348146121076	8.68006806631895	1.46183792015432
C	1.73214983441238	1.48155437491618	-0.18647077241583
H	1.85484375726695	1.52720287443654	-1.26486608136517
C	-4.91865865690089	5.34218804981635	5.71673295711508
H	-5.68323312898352	4.57565683681261	5.81197963427115
C	3.60090763050147	8.30870660831694	6.30311308908923
H	4.16001552707407	8.95075612935575	6.99393392524335
H	4.16716781007654	8.23667117466063	5.36967121830325
H	3.55481467138573	7.30927148350824	6.74956230191283
C	1.75421477366958	0.22152653389724	1.86920638821094
H	1.89598339018585	-0.71624849436849	2.39886009755578
C	-0.92618675764784	13.59177215253595	4.16593368070068
H	-0.19246559192474	14.39811450865680	4.05101990352733
H	-1.05786684337875	13.40833425831041	5.23665158009432
H	-1.88110756558281	13.94270176186957	3.75842156451559
C	1.91486812123292	0.27490339103737	0.48728726408866
H	2.18137861889922	-0.62195668951284	-0.06462302654722

Sn(IV)′

Sn	5.57498238476371	24.91322486365364	2.56878838760108

Si	4.26875358557946	25.55374609830642	0.42653163061447
N	5.27825950171051	24.71570470571341	6.51724450336873
N	6.65024392643814	22.98260679266260	2.23954820078173
N	5.40942198855165	27.00019749269594	6.44089235455905
N	5.12411677551814	25.89935846109774	4.16388259391864
N	7.70542826956612	24.91856088955928	2.00026635542985
C	5.04253459875980	23.33391458179621	6.31446713069422
C	6.10181948620790	22.42041606687796	6.47888487407041
C	7.77727936685334	23.59470611470445	1.86090114728067
C	3.73867300993788	22.88587637714148	6.01920397354247
C	5.46060276845129	28.37711323554371	6.10274891846014
C	5.38598350047464	25.22546897594614	7.82122030425760
H	5.37393857131746	24.57696858063953	8.68686664405682
C	8.96844471852967	22.89422203519290	1.29771942758177
C	6.14586897618578	21.64063953861006	1.88217057475731
C	5.47246499124622	26.57346812472736	7.77264023110104
H	5.54452664465556	27.27376460904235	8.59358260215152
C	3.51616538918001	21.51138928174754	5.91006049453527
H	2.51414483503581	21.14743354733702	5.69550091909083
C	4.55121571921454	20.60001700230879	6.07965704938958
H	4.35780166857978	19.53398263511198	5.99271836411802
C	4.30695649417093	29.00394152488490	5.57851940062308
C	5.83467693157024	21.05476662629945	6.35569071635491
H	6.64145636543204	20.33679068072121	6.48393411046196
C	9.17399366872708	22.90312239923544	-0.08395321162179
H	8.46072432811284	23.41039901777177	-0.72824919076796
C	6.64766002903408	29.10441032347884	6.31011194619659
C	7.50853132121943	22.89111508361195	6.79751624393911
H	7.48937765813337	23.98497825556306	6.83975835794758
C	3.01942406120431	28.21882916856780	5.39564339926786
H	3.29707451215747	27.23939391029191	4.98857791518413
C	2.57573786559864	23.84979763973582	5.87502000455217
H	2.97360705453488	24.86671237589798	5.94810633245901
C	8.78030971801062	25.91590686527515	2.16307888310043
C	9.89260608217724	22.26100416782045	2.12937605054947
H	9.73209444516288	22.24466752802648	3.20303029350552
C	4.74606587754349	21.56933670018670	2.50205081008747
H	4.79290882608635	21.76169686747417	3.57799459443858
H	4.30559186600912	20.57954678710255	2.34599709170206
H	4.07721570472482	22.30909349172892	2.04278839081813
C	11.01659760301271	21.64933782138583	1.58231655647185
H	11.73537232466143	21.16411189009232	2.23628195453987
C	5.52968195599205	31.10037918028016	5.50960268399584
H	5.55254602470131	32.16316218701174	5.28332132011302
B	5.29443635980884	25.82548520015970	5.55168643704659
C	6.03387466704029	21.45965027330629	0.36267757062170
H	5.45577613106401	22.27971163402578	-0.07774967196834
H	5.51966737539929	20.51882116559594	0.13681471357937
H	7.01784221548990	21.42947983028035	-0.11288712677850
C	10.29019053753704	22.27882151796966	-0.62812189719251
H	10.44129114048425	22.28682541565907	-1.70369920672834
C	4.36955222854192	30.36578050892933	5.28308873010979
H	3.49709184426152	30.86991709359263	4.87957937165554
C	1.89318356007335	23.72102186429894	4.50805090800449
H	1.45317512943746	22.72679592135343	4.36653539272772
H	2.61242035155522	23.89701215501344	3.70037884645477
H	1.09055643938957	24.46182373562208	4.41435364598902
C	7.00889839263698	20.52601750017017	2.49005281140265
H	7.99786959212138	20.48613376994142	2.02757491305503
H	6.51886669344567	19.55943959433309	2.33056748697700
H	7.12586516020036	20.68115985536253	3.56742009292508
C	11.21666861950679	21.65459611785671	0.20454182576895
H	12.09285210305452	21.17333820584645	-0.22033584496790
C	6.65581572851087	30.47223826555005	6.01914658784801
H	7.56244027801767	31.04935086913023	6.18307026016937
C	8.07784230637459	27.28096544911114	2.15201302833422
H	8.80931557275263	28.08364479591227	2.28691225858789
H	7.33904082641881	27.34867901999082	2.95985895177311

H	7.56563945810984	27.44017644754442	1.19530680458964
C	5.47349741821487	25.50937429840694	-1.01886278468224
H	4.95596019004755	25.77527435534284	-1.94910967857461
H	5.91011530857803	24.51284843388179	-1.14618411309283
H	6.29312615914123	26.21970208353969	-0.86695027871658
C	7.91569159434214	28.44550823882804	6.82231568139084
H	7.73801212685071	27.36512597052592	6.86089225844221
C	8.48862845856426	22.49012413107565	5.68895575208597
H	9.48202447732596	22.91039215176920	5.88647118544781
H	8.13831580691287	22.86309389206616	4.72120653953570
H	8.59016837750139	21.40035403303062	5.61805892312704
C	9.82109625013539	25.89458651303155	1.03471830149390
H	9.33240597860825	25.93976716239194	0.05534202630795
H	10.45168497735224	25.00346770296452	1.06849510230188
H	10.46950557382372	26.77150239499131	1.13608099041424
C	1.56490771764158	23.66224081155144	7.01534619363834
H	0.76389770213011	24.40632236095914	6.93860036327603
H	2.05056678378524	23.78048620501696	7.98988315607197
H	1.10746410807708	22.66642038701368	6.98136349571813
C	2.32180174162569	27.99493420849149	6.74766717892556
H	2.97614318950537	27.47527715430724	7.45404942243719
H	1.41752260750269	27.38923438737318	6.61138496210120
H	2.02676163290628	28.95454141027270	7.19004342463452
C	7.97887923505045	22.37294628681744	8.16278615734827
H	8.04394114644350	21.27867614970557	8.16939565436057
H	7.28858012162581	22.67514829096028	8.95718535257749
H	8.97162621014210	22.77176301146913	8.40020639463451
C	9.46633617743874	25.70382443298474	3.52038361716450
H	10.20581118768590	26.49162726522513	3.70114791915426
H	9.98155522235751	24.73791610927228	3.54711428042702
H	8.72716800198684	25.72925583582337	4.32944479149874
C	2.83244151408995	24.37327276341075	0.14211200842677
H	2.17332494168442	24.33320381376049	1.01608526970280
H	3.17429037691289	23.35605280191992	-0.07500765571466
H	2.23957549019667	24.72003494655996	-0.71386527709744
C	9.09832710960577	28.69011658615674	5.87486759729998
H	8.84787627192087	28.39197580383257	4.85276952870870
H	9.96951641295578	28.10927965998214	6.19956379110487
H	9.38911811903755	29.74653396156195	5.85989444116363
C	3.63338779713018	27.29350522195552	0.74222290483821
H	3.07206208873188	27.65820366790792	-0.12716308001643
H	4.45592065904587	27.98924769428401	0.93948131866394
H	2.96921637877173	27.30666093324784	1.61296890280035
C	8.26169036572652	28.92226252908003	8.24059416377243
H	8.47333970724343	29.99808828100123	8.24725105415117
H	9.14998428925081	28.40021215796586	8.61461483632625
H	7.43725092301447	28.73791854168234	8.93631476981042
C	2.04448643180306	28.85472001541826	4.40223940507920
H	1.60635296564901	29.78239370237083	4.78960309468114
H	1.21854972594448	28.16111949390950	4.20967071407247
H	2.53143884201089	29.07869504547439	3.44677171751043

6. References for supporting information

s1. Y. Wang, A. Crumpton, M. Ellwanger, C. McManus, S. Aldridge, *Angew. Chem. Int. Ed.* **2024**, *63*, e202402795.

s2. (a) F. Neese, *WIREs Computational Molecular Science* **2012**, *2*, 73-78; (b) F. Neese, F. Wennmohs, U. Becker, C. Riplinger, *J. Chem. Phys.* **2020**, *152*, 224108; (c) F. Neese, *WIREs Computational Molecular Science* **2018**, *8*, e1327.

s3. (a) J. W. Furness, A. D. Kaplan, J. Ning, J. P. Perdew, J. Sun, *J. Phys. Chem. Lett.* **2020**, *11*, 8208-8215; (b) J. W. Furness, A. D. Kaplan, J. Ning, J. P. Perdew, J. Sun, *J. Phys. Chem. Lett.* **2020**, *11*, 9248-9248.

s4. S. Grimme, A. Hansen, S. Ehlert, J.-M. Mewes, *J. Chem. Phys.* **2021**, *154*, 064103.

s5. (a) E. Caldeweyher, C. Bannwarth, S. Grimme, *J. Chem. Phys.* **2017**, *147*, 034112; (b) E. Caldeweyher, S. Ehlert, A. Hansen, H. Neugebauer, S. Spicher, C. Bannwarth, S. Grimme, *J. Chem. Phys.* **2019**, *150*, 154122.

s6. M. Garcia-Ratés, F. Neese, *J. Comput. Chem.* **2020**, *41*, 922–939.
